# Solvent-Dependent Carbon-to-Metal Hydrogen Atom Transfer Reactivity of a Square Planar Rhodium(II) Alkynyl Complex

**DOI:** 10.1021/acs.organomet.6c00053

**Published:** 2026-04-01

**Authors:** Thomas M. Hood, Sophie H. Dewick, Anjali John, Jeremiah P. Tidey, Julie V. Macpherson, Ragnar Bjornsson, Emma Richards, Tobias Krämer, Adrian B. Chaplin

**Affiliations:** † Department of Chemistry, 2707 University of Warwick, Coventry CV4 7AL, U.K.; ‡ Department of Physics, University of Warwick, Coventry CV4 7AL, U.K.; § Laboratoire de Chimie et Biologie des Métaux, Université Grenoble Alpes, CNRS, CEA, IRIG, F-38054 Grenoble, Cedex, France; ∥ School of Chemistry, Cardiff University, Translational Research Hub, Cardiff CF24 4HQ, U.K.; ⊥ School of Chemistry, Trinity College Dublin, The University of Dublin, Dublin 2, Ireland

## Abstract

We report on the synthesis and characterization of the square planar rhodium­(II) alkynyl complex [Rh­(PNP-*t*Bu)­(C≡C*t*Bu)]^+^ and its transformation into the vinylidene derivative [Rh­(PNP-*t*Bu)­(C=CH*t*Bu)]^+^ by reaction with 9,10-dihydroanthracene. Computational analysis supports a mechanism involving carbon-to-metal hydrogen atom transfer followed by 1,3-hydride migration, and intermediate formation of the associated rhodium­(III) alkynyl hydride [Rh­(PNP-*t*Bu)­H­(C≡C*t*Bu)]^+^ has been substantiated experimentally. Study of [Rh­(PNP-*t*Bu)­(C≡C*t*Bu)]^+^ in the solid state by EPR spectroscopy, supplemented by multireference CAS­(9,6)/NEVPT2 calculations, enabled assignment as a metal-centered radical to be corroborated, while analysis of frozen glass solutions revealed the presence of square pyramidal solvent adducts of tetrahydrofuran, 2-methyltetrahydrofuran, 1,2-difluorobenzene, fluorobenzene, and α,α,α-trifluorotoluene. Consistent with a carbon-to-metal hydrogen atom transfer mechanism, the extent of solvent coordination measured by EPR spectroscopy inversely correlates with the rate at which the metalloradical reacts with 9,10-dihydroanthracene.

## Graphical abstract

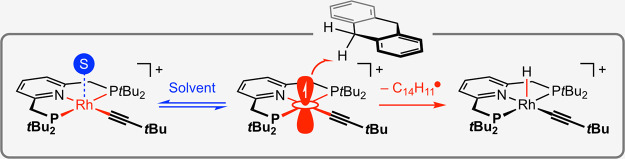

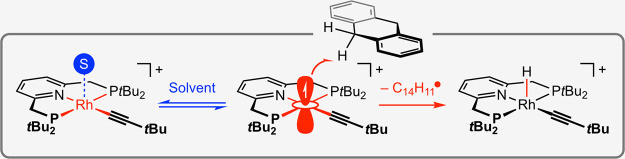

## Introduction

The selective activation of aliphatic C–H bonds is a synthetically challenging task with broad applications in organic chemistry.^1^ While organometallic approaches have enabled considerable progress to be made, these developments are largely derived from the study of closed-shell precious metal complexes that operate via two-electron processes.^2^ Seminal work by Wayland using rhodium­(II) porphyrin complexes established that metal-centered radicals can also react with C–H bonds by one-electron processes to enact homolytic cleavage (Figure 1A).^3,4^ A biradical mechanism that involves synchronous formation of Rh^III^–H and Rh^III^–C bonds was proposed and has since been invoked in the cyclometalation of a triphenylphosphine-ligated rhodium­(II) metalloradical and allylic C­(sp^3^)–H bond activation reactions of M^II^(cyclooctadiene) complexes (M = Rh, Ir).^5,6^ Independent carbon-to-metal hydrogen atom transfer is a more entropically favorable and synthetically attractive manifold for radical C–H bond oxidative addition and was first demonstrated by Bullock and co-workers using transient, photochemically generated, osmium­(I) metalloradicals (Figure 1A).^7^ More recently, we have observed this open-shell mechanism in the onward reactivity of platinum­(I) bis­(phosphine) complexes (Figure 1A).^8^

**Figure 1:**
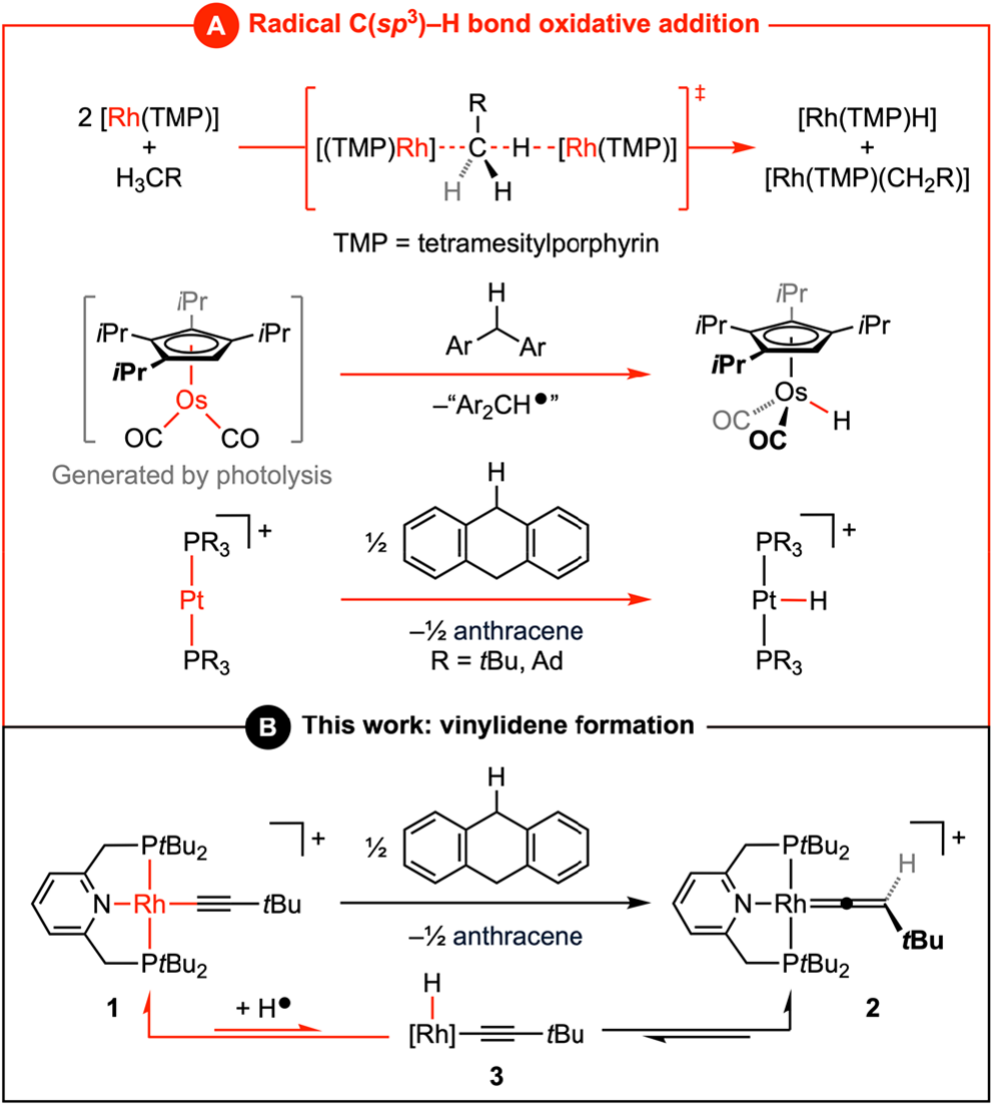
Activation of C­(sp^3^)–H bonds by precious-metal-based metalloradicals.

For a carbon-to-metal hydrogen atom transfer reaction to occur, formation of a strong M–H bond is required to offset the energetic cost of homolytic C–H bond cleavage. For instance, while [Pt­(P*t*Bu_3_)_2_]^+^ reacts with 9,10-dihydroanthracene at room temperature to afford [Pt­(P*t*Bu_3_)_2_H]^+^, consistent with the considerably smaller M^II^–H bond dissociation energy calculated for the product (*D*_e_ = 64.4 vs 78.5 kcal·mol^–1^), the palladium­(I) analogue does not.^8^ Highlighting the decisive thermodynamic role of M–C bond formation in the biradical mechanism and challenging the viability of rhodium­(II)-based carbon-to-metal hydrogen atom transfer reactions, a Rh^III^–H bond dissociation energy of only 61.1 kcal·mol^–1^ has been determined for Wayland’s porphyrin-based system.^3^

Inspired by Wayland’s work and having shown that operationally unsaturated rhodium­(I) pincer complexes induce rapid alkyne-to-vinylidene tautomerisation,^9^ we identified square planar rhodium­(II) alkynyl complexes as candidates for radical C–H bond oxidative addition reactivity, reasoning that net 1,3-hydride migration from the metal to β-carbon of the alkynyl would provide a supplementary thermodynamic driving force.^10^ Redox induced transformations of alkynyl complexes that yield vinylidene derivatives have been reported in the literature, but are limited to coordinatively unsaturated examples where this outcome is rationalized by significant spin localization on the β-carbon and competes with dimerization by C_β_–C_β_ bond formation.^11^ We herein report on the experimental and computational evaluation of our hypothesis using rhodium­(II) alkynyl complex [Rh­(PNP-*t*Bu)­(C≡C*t*Bu)]­[BAr^F^_4_] **1** (Ar^F^ = 3,5-(CF_3_)_2_C_6_H_3_), where formation of the rhodium vinylidene derivative [Rh­(PNP-*t*Bu)­(C=CH*t*Bu)]­[BAr^F^_4_] **2** is proposed to proceed by carbon-to-metal hydrogen atom transfer to **1** with intermediate formation of the rhodium­(III) alkynyl hydride [Rh­(PNP-*t*Bu)­H­(C≡C*t*Bu)]­[BAr^F^_4_] **3** (Figure 1B).

## Results and Discussion

### Synthesis and Characterization of **1**

Phosphine-based pincer ligands have previously been shown to support the isolation of mononuclear rhodium­(II) complexes by one-electron oxidation.^12–14^ To access the rhodium­(I) alkynyl required in this instance, [Rh­(PNP-*t*Bu)­(C≡C*t*Bu)] **4**, a synthetic route that leverages the capacity of dearomatized dinitrogen pincer complex [Rh­(PNP*-*t*Bu)­(N_2_)] **5** to activate C–H bonds by metal–ligand cooperativity was devised (Figure 2A).^15,16^ Treatment of **5** with excess HC≡C*t*Bu in cyclohexane was subsequently found to afford **4** selectively at RT and the product was thereafter isolated in 82% yield on a preparative scale. Complex **4** is characterized by time averaged *C*_2*v*_ symmetry in C_6_D_12_ solution, displaying a doublet ^31^P resonance at δ 64.3 (^1^ *J*_RhP_ = 155 Hz) and structurally diagnostic doublet of triplet ^13^C resonance at δ 96.1 for the alkynyl ligand (^1^ *J*_RhC_ = 48 Hz; ^2^ *J*_PC_ = 19 Hz). Analysis of the isolated complex by cyclic voltammetry in 1,2-difluorobenzene (DFB),^17^ using [*n*Bu_4_N]­[BAr^F^_4_] as the electrolyte, indicated that **4** undergoes reversible one-electron oxidation with *E*_1/2_ = −0.64 V vs Fc^+^/Fc (Fc = ferrocene, Figure 2B). Under these conditions [Rh­(PNP-*t*Bu)­(CH_3_)] was found to exhibit very similar redox characteristics (*E*_1/2_ = −0.63 V vs Fc^+^/Fc) while [Rh­(PNP-*t*Bu)­Cl] shows a more positive redox potential (*E*_1/2_ = −0.37 V vs Fc^+^/Fc).^12,18^ Encouraged by these data, **4** was reacted with one equivalent of Fc­[BAr^F^_4_] in DFB which enabled **1** to be isolated in 87% yield as deep purple crystals.^19^

**Figure 2:**
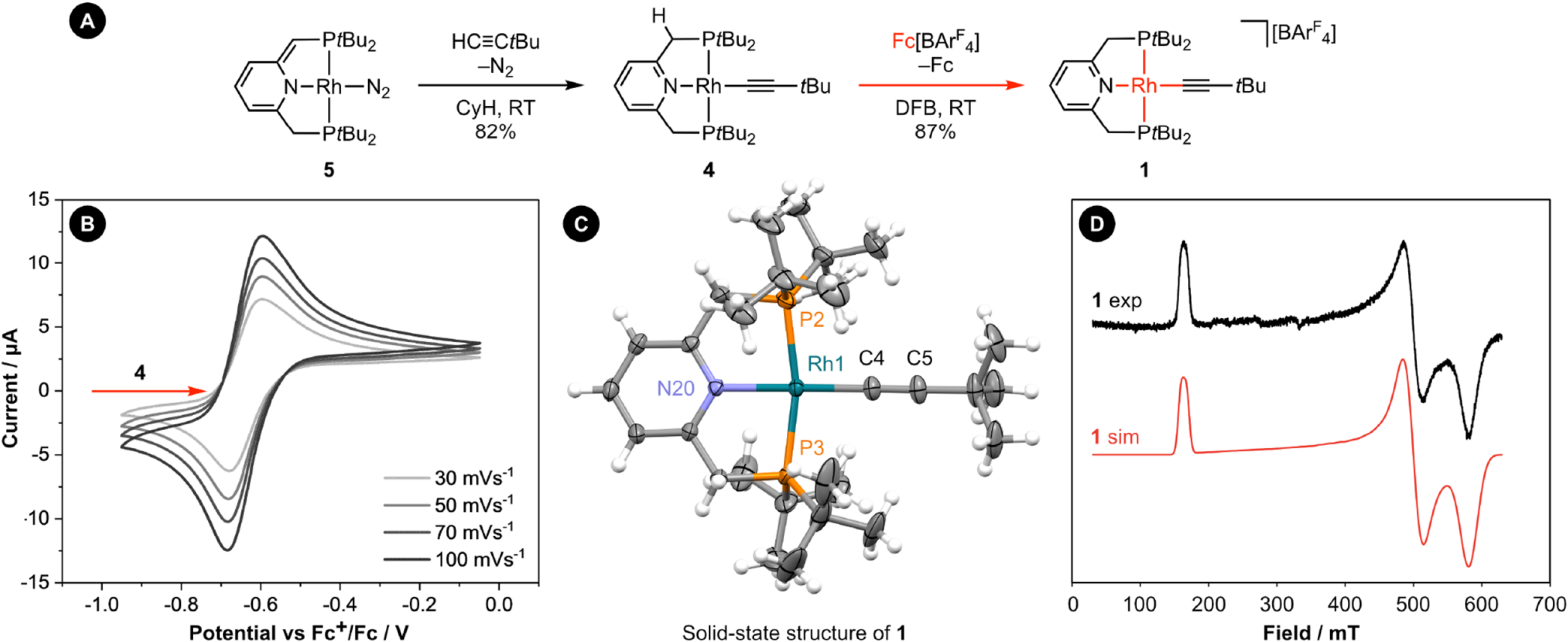
(A) Synthesis of rhodium­(II) alkynyl complex **1**. (B) Cyclic voltammograms for the oxidation of **4** (*E*_1/2_ = −0.64 V, *i*_P_^red^/*i*_P_^ox^ = 0.99) in DFB at RT (2 mM **4**; 0.2 M [*n*Bu_4_N]­[BAr^F^_4_] electrolyte; glassy carbon working electrode, coiled Pt wire counter electrode, and Ag wire quasi-reference electrode). (C) Solid-state structure of **1** with anion omitted for clarity and thermal ellipsoids at 50%; selected metrics: **1**, Rh1–P2, 2.3139(6) Å; Rh1–P3, 2.3046(6) Å; Rh1–N20, 2.0960(15) Å; Rh1–C4, 1.975(2) Å; C4–C5, 1.205(3) Å; P2–Rh1–P3, 166.72(2)°; N20–Rh1–C4, 177.54(8)°; **4** (not shown), Rh1–P2, 2.2489(9) Å; Rh1–P3, 2.2524(10) Å; Rh1–N20, 2.065(3) Å; Rh1–C4, 1.975(4) Å; C4–C5, 1.225(6) Å; P2–Rh1–P3, 168.72(4)°; N20–Rh1–C4, 178.57(12)°. (D) Experimental and simulated CW X-band EPR spectra of **1** recorded in the solid state at 115 K (1:10 w/w dispersion of powdered sample in [*n*Bu_4_N]­[BAr^F^_4_]).

Formation of **1** was corroborated in the solid state by single crystal X-ray diffraction, with the complex notable for adoption of a square planar coordination geometry (Figure 2C). There are only minor structural perturbations relative to **4**, with the most pronounced being a + 0.059(7) Å elongation of the Rh–P bonds in **1**. There is no evidence for bond length alternation in the pyridine ring nor any statistically significant differences in the Rh–C nor C≡C bond lengths. The presence of a single paramagnetic species of *C*_2v_ symmetry is evident in the ^1^H NMR spectrum of **1** in DFB at RT. All the paramagnetically shifted proton environments can be accounted for between δ −10 and +40 and an effective magnetic moment of 1.74 μ_B_ was determined using Evans’ method, consistent with the expected d^7^-metal configuration.^20^ No ^31^P resonance could be located between δ −1000 and +1000 but the complex presents a strong peak at +579.2637 (calcd 579.2625) *m*/*z* for the intact cation in the positive ion ESI-MS spectrum.

Assignment of **1** as a metal-centered radical was confirmed by studying a dilute powdered sample by EPR spectroscopy at 115 K (Figure 2D). Under these conditions, **1** displays rhombic symmetry with a high degree of *g*-anisotropy (*g*_1_ = 4.051, *g*_2_ = 1.328, *g*_3_ = 1.140) that is characteristic of authentically square planar rhodium­(II) complexes.^13,21^ No hyperfine coupling to rhodium was resolved. Multireference calculations performed at the NEVPT2 level of theory with a CAS­(9,6) active space, in combination with a scalar relativistic X2C Hamiltonian and Weigend’s x2c-TZVPall-electron basis set,^22^ corroborate this assignment and enable the observed *g*-parameters to be qualitatively reproduced (*g*_1_ = 4.547, *g*_2_ = 1.361, *g*_3_ = 1.167).^23^ The electronic structure of **1** is dominated by a (d_*xy*_)^2^(d_*yz*_)^2^(d_*xz*_)^2^(d_*z*2_)^1^ ground state configuration and the spin density is correspondingly concentrated in the rhodium d_*z*2_ orbital (89%, Figure 3A). The first excited state has a (d_*xy*_)^2^(d_*yz*_)^2^(d_*z*2_)^2^(d_*xz*_)^1^ electronic configuration and is calculated to lie only 987.8 cm^–1^ higher in energy, consistent with the large *g*-anisotropy.^24^

**Figure 3:**
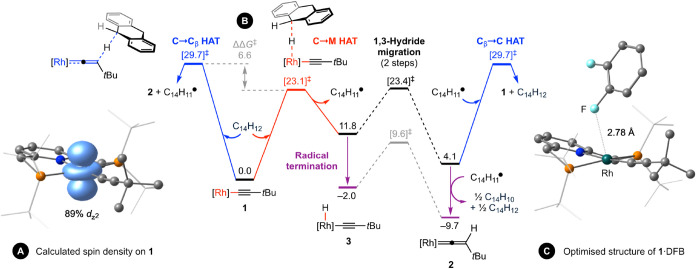
(A) Spin density for the ground state of **1** from CAS­(9,6)/NEVPT2 calculations. (B) Computed reaction profiles for hydrogen atom transfer reactions of **1** (Δ*G*_298K_ in kcal·mol^–1^) and (C) optimized structure of **1**·DFB at the M06-D3/SDD/6–311++G­(2d,2p)//M06L-D3/SDD/6–31G** level of theory corrected for DFB solvent. Calculations carried out excluding the counterion. [Rh] = Rh­(PNP-*t*Bu)^+^

### Reaction of **1** with 9,10-Dihydroanthracene in DFB

Having confirmed **1** as a metalloradical, we set about assessing its capacity to undergo carbon-to-metal hydrogen atom transfer using 9,10-dihydroanthracene as the donor (*D*_e_ ∼ 77 kcal·mol^–1^).^25^ Building upon preceding work,^8^ **1** was treated with an excess of dihydroanthracene in DFB and the ensuing reactivity gauged *in situ* by NMR spectroscopy. Consistent with our hypothesis, slow but selective conversion into **2** was established at 60 °C through appearance of a doublet ^31^P resonance at δ 65.6 (^1^ *J*_RhP_ = 138 Hz) which increases in intensity alongside the characteristic ^1^H resonances of the vinylidene complex and half an equivalent of anthracene.^9,26^ After 1 day, ∼23% of **1** had been consumed with an additional 13 days required for complete conversion into **2**.

To probe the intricacies of this transformation we turned to computational methods, in particular the use of unrestricted DFT calculations to quantify the relative energetics of pathways that commence with carbon-to-metal and carbon-to-β-carbon hydrogen atom transfer (Figure 3B).^27^ Analysis at the M06-D3/SDD/6–311++G­(2d,2p)//M06L-D3/SDD/6–31G** level of theory corrected for DFB solvent indicates that the former, metal centered, mechanism is kinetically preferred with an absolute barrier of Δ*G*_298K_^‡^ = 23.1 kcal·mol^–1^ that is distinctly lower than the latter (ΔΔ*G*_298K_^‡^ = −6.6 kcal·mol^–1^).^28^ Corresponding formation of **3** is endergonic by 11.8 kcal·mol^–1^ in line with a calculated Rh^III^–H bond dissociation energy of only *D*_e_ = 64.3 kcal·mol^–1^.^29^ Subsequent 1,3-hydride migration provides an additional thermodynamic driving force (ΔΔ*G*_298K_ = −7.7 kcal·mol^–1^) but is not sufficient to render formation of **2** from **1** exergonic alone. This transformation is, however, kinetically competent with reformation of **1** by radical rebound (ΔΔ*G*_298K_^‡^ < 1 kcal·mol^–1^) and consequently helps bias loss of the hydroanthracenyl radical into solution. From the computed thermodynamics, termination of this radical into anthracene (Δ*G*_298K_ = −13.8 kcal·mol^–1^) is ultimately required for the formation of **2** to be exergonic and presumably occurs via carbon-to-metal hydrogen atom transfer to a second equivalent of **1**.^26^

In experimental support of facile interconversion between **2** and **3**, dissolution of **2** in CD_3_CN at room temperature resulted in equilibrium formation of solvent stabilized rhodium­(III) hydride adduct [Rh­(PNP-*t*Bu)­H­(C≡C*t*Bu)­(NCMe)]­[BAr^F^_4_] (**3**·ACN*; δ*_31P_ 71.5, ^1^ *J*_RhP_ = 101 Hz; δ_1H_ – 16.66, ^1^ *J*_RhH_ = 19.8 Hz; ^2^ *J*_PH_ = 10.9 Hz). Van’t Hoff analysis of the equilibrium between 0 and 70 °C using ^1^H NMR spectroscopy enabled the thermodynamic parameters to be determined (*K* = [**3**·ACN]/[**2**]­[CD_3_CN]; Δ*H* = −7.81 ± 0.09 kcal·mol^–1^, Δ*S =* −30.5 ± 0.3 cal·mol^–1^K^–1^, Δ*G*_298K_ = +1.3 ± 0.2 kcal·mol^–1^), which reflect the need for acetonitrile to be present in vast excess to shift the equilibrium to the hydride and corroborate the spontaneous formation of **2** from **3** in the absence of a strongly coordinating solvent. The thermodynamics are well reproduced computationally (Δ*G*_298 K_ = +4.8 kcal·mol^–1^).

### Solvent Adducts of **1**

To gain further insight into the behavior of **1** in solution, the CW X-band EPR spectrum of this metalloradical was recorded in a frozen glass of 4:1 v/v DFB/cyclohexane at 115 K (Figure 4A) revealing the presence of two electronically distinct complexes in a 1:1.25 ratio.^30^ By comparison to the solid-state data, the minor species is assigned to **1**, with *g*_2_ and *g*_3_ almost coincident and only distinguished through spectral fitting (*g*_1_ = 3.982, *g*_2_ = 1.286, *g*_3_ = 1.274; coupling of 307 MHz to ^103^Rh along *g*_1_). The major species displays reduced *g*-anisotropy (*g*_1_ = 2.441, *g*_2_ = 2.301, *g*_3_ = 1.970; coupling of 113 MHz to ^103^Rh spectroscopically resolved along *g*_3_) and is assigned to a square pyramidal solvent adduct **1**·DFB. This assignment is supported by literature precedents^6,31^ and computationally, with the optimized structure of **1**·DFB exhibiting an electrostatic Rh···F interaction of 2.78 Å (Figure 3C) and enabling the observed change in *g*-parameters to be satisfactorily reproduced (*g*_1_ = 3.461, *g*_2_ = 2.314, *g*_3_ = 1.816).^22,23^ The reduction in *g*-anisotropy relative to **1** is attributed to destabilization of the d_z2_ orbital by interaction with the solvent and this suggestion is supported by multireference calculations, which indicate that there is increased separation between the ground and first excited states (2984.8 cm^–1^).^24^

**Figure 4:**
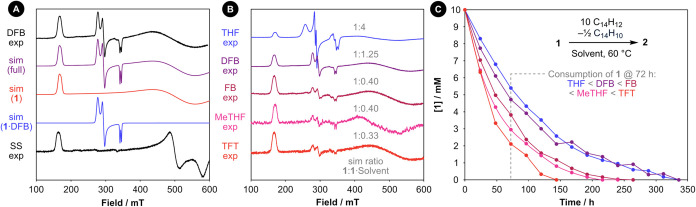
(A) Experimental and simulated CW X-band EPR spectra of **1** recorded in 4:1 v/v DFB/cyclohexane frozen glass at 115 K, with the corresponding solid-state spectrum for comparison. (B) Experimental CW X-band EPR spectra of **1** recorded in various 4:1 v/v solvent/cyclohexane frozen glasses at 115 K. (C) Time course data for the reaction of **1** with 9,10-dihydroanthracene in various solvents.

After verifying the solution stability at room temperature (24 h), additional solvent adducts of **1** were investigated by EPR spectroscopy at 115 K in frozen glasses prepared from 4:1 v/v mixtures of tetrahydrofuran (THF), 2-methyltetrahydrofuran (MeTHF), fluorobenzene (FB), and α,α,α-trifluorotoluene (TFT) with cyclohexane (Figure 4B). Solvent coordination is evident in all cases and the extent increases in the order TFT < MeTHF ≈ FB < DFB < THF, with **1**·THF accounting for 80% of the sample in THF/cyclohexane (**1**:**1**·THF = 1:4).^32^

Although solvent coordination to **1** could not be detected in solution by NMR spectroscopy, based on the preceding mechanistic analysis, we reasoned that it should nevertheless retard the reactivity of the metalloradical. The reaction between **1** and excess 9,10-dihydroanthracene was consequently re-examined in the additional solvents, under otherwise equivalent conditions. Consistent with pre-equilibrium kinetics and rate determining carbon-to-metal hydrogen atom transfer to **1**, the differences in solvent binding strength observed by EPR spectroscopy are mirrored in the kinetics of the reaction. The rate at which **1** is consumed decreases in the order TFT > MeTHF > FB > DFB > THF (Figure 4C).

## Conclusions

Rhodium­(II) alkynyl complex [Rh­(PNP-*t*Bu)­(C≡C*t*Bu)]­[BAr^F^_4_] **1** is an authentically square planar metalloradical that activates the C­(sp^3^)–H bonds of 9,10-dihydroanthracene, yielding vinylidene derivative [Rh­(PNP-*t*Bu)­(C=CH*t*Bu)]­[BAr^F^_4_] **2** and anthracene. Computational analysis substantiates a mechanism commencing with carbon-to-metal hydrogen atom transfer and intermediate formation of the rhodium­(III) alkynyl hydride [Rh­(PNP-*t*Bu)­H­(C≡C*t*Bu)]­[BAr^F^_4_] **3**, with subsequent 1,3-hydride migration favoring the overall transformation thermodynamically and helping to bias escape of the hydroanthracenyl radical from the metal coordination sphere. This mechanistic proposal is supported experimentally through equilibrium formation of [Rh­(PNP-*t*Bu)­H­(C≡C*t*Bu)­(NCMe)]­[BAr^F^_4_] **3**·ACN from **2** and observation of a significant solvent dependence on the rate of reaction between **1** and 9,10-dihydroanthracene that is attributed to equilibrium solvent coordination by detailed analysis of the metalloradical in frozen glasses of varying composition by EPR spectroscopy. These findings reaffirm the capacity of metalloradicals to active C–H bonds by a hydrogen atom transfer mechanism and, more broadly, underscore the potential of platinum group metals to participate in synthetically useful open-shell reactivity.

## Experimental Section

### General Methods

All manipulations were performed under an atmosphere of argon (1 atm) using Schlenk and glovebox techniques unless otherwise stated. Room temperature (RT) is approximately 293 K. Glassware was oven-dried at 150 °C overnight and flame-dried under vacuum prior to use. Molecular sieves were activated by heating at 300 °C *in vacuo* overnight. Anhydrous tetrahydrofuran (THF) and 2-methyltetrahydrofuran (MeTHF) were vacuum distilled from sodium/benzophenone and stored over 3 Å molecular sieves. Anhydrous hexane was purchased from Sigma-Aldrich, degassed by sparging with argon and stored over 3 Å molecular sieves. The fluorinated solvents C_6_H_5_F (FB), 1,2-C_6_H_4_F_2_ (DFB) and C_6_H_5_CF_3_ (TFT) were predried over Al_2_O_3_, distilled from calcium hydride and twice dried over 3 Å molecular sieves.^17^ Cyclohexane, C_6_D_12_ and SiMe_4_ were dried over Na/K alloy overnight, vacuum distilled, freeze–pump–thaw degassed and stored over a potassium mirror. CD_2_Cl_2_, CD_3_CN and 3,3-dimethylbutyne were freeze–pump–thaw degassed and dried over 3 Å molecular sieves. PNP-*t*Bu,^33^ [Rh­(COD)_2_]­BF_4_,^34^ [Rh­(PNP-*t*Bu)­Cl],^35^ [Rh­(PNP-*t*Bu)­(CH_3_)],^18^ [*n*Bu_4_N]­[BAr^F^_4_][Bibr ref36] and Fc­[BAr^F^_4_][Bibr ref19] were prepared according to published procedures. Potassium *tert*-butoxide and 9,10-dihydroanthracene were purchased from commercial suppliers and stored under argon.

NMR spectra were recorded on Bruker spectrometers under argon at 298 K unless otherwise stated. Chemical shifts are quoted in ppm and coupling constants in Hz. Virtual coupling constants are reported as the separation between the first and third lines.^37^ NMR spectra in proteo solvents were recorded using an internal capillary of C_6_D_6_. EPR measurements were recorded on a Bruker EMX spectrometer utilizing an ER 072 magnet/ER 081 power supply combination (maximum field 0.65 T), an ER4102ST resonator, operating at 100 kHz field modulation, 0.3 mT modulation depth and 10 mW microwave power. Simulations of all EPR spectra were performed using garlic or pepper functions within the Easyspin toolbox.^38^

Cyclic voltammetry (CV) experiments were carried out in an inert atmosphere glovebox using an Autolab PGSTAT101 potentiostat (Metrohm, Switzerland) and a 3-electrode setup comprising a glassy carbon (CH instruments, 3.0 mm diameter disk) working electrode, a coiled platinum wire counter electrode, and a silver wire quasi-reference electrode. All measurements were carried out using 2 mM of the complex investigated, DFB as the solvent, and 0.2 M [*n*Bu_4_N]­[BAr^F^_4_] as the supporting electrolyte. Ferrocene was added at the end of each experiment as an internal reference, and its half wave potentials (*E*_1/2_) were recorded. *E*_1/2_ were calculated using the formula (*E*_p_^ox^ + *E*_p_^red^)/2, where *E*_p_^ox^ and *E*_p_^red^ are the oxidation (anodic) and reduction (cathodic) peak potential values, and all potentials were calibrated against the *E*_1/2_ of the Fc^+^/Fc redox couple. Three scans are recorded for each measurement. Data presented in the figures is the third scan and arrows are used to indicate the direction of the first scan. The peak current ratio *i*_p_^red^/*i*_p_^ox^ was determined by fitting the 100 mVs^–1^ data using Nova 2.1.7 software (Metrohm, Switzerland). Linear relationships between the voltammogram peak current and square root of the potential sweep scan rate were observed in all cases, indicating a diffusion-controlled process.

Crystallographic data were collected on a Rigaku Oxford Diffraction SuperNova diffractometer equipped with a AtlasS2 CCD or HyPix-6000HE hybrid photon counting detector using Mo Kα or Cu Kα radiation and an Oxford Cryosystems N-HeliX cryostat (100 or 150 K). Data were collected and reduced using CrysAlisPro.^39^ The structures were solved using SHELXT and refined using SHELXL, through the Olex2 interface.^40,41^ All non-hydrogen atoms were refined anisotropically. Hydrogen atoms were placed in calculated positions and refined using the riding model. Full details for all structures reported are documented in the CIF, which have been deposited with the Cambridge Crystallographic Data Centre under CCDC 2500870–2500872.

High resolution (HR) ESI-MS were recorded on Bruker Maxis Plus instrument.

### Preparation and Characterization of [Rh­(PNP*-*t*Bu)­(N_2_)] **5**

This complex was first reported by Heinekey and Goldberg,^18^ and was prepared via a refined procedure involving deprotonation of the synthetically convenient precursor [Rh­(PNP-*t*Bu)­(N_2_)]­BF_4_ with KO*t*Bu in a hexane suspension.^42^ In our hands, this procedure reliably provides **5** in high purity. Single crystals suitable for analysis by X-ray diffraction were also obtained, allowing structural elucidation of **5** in the solid state for the first time.^43^ All manipulations were performed under an atmosphere of dinitrogen (1 atm) unless otherwise stated.

#### Step 1: Preparation of [Rh­(PNP-*t*Bu)­(N_2_)]­BF_4_

A solution of [Rh­(COD)_2_]­BF_4_ (51.3 mg, 126 μmol) and PNP-*t*Bu (50.2 mg, 127 μmol) in DFB (8 mL) was sonicated for 5 min, freeze–pump–thaw degassed, placed under dinitrogen (1 atm), and then stirred at RT for 3.5 h. The resulting golden yellow solution was blown down under a stream of dinitrogen and then washed with hexane (2 × 5 mL). Recrystallization from DFB/hexane at RT afforded the product as a microcrystalline yellow solid. Yield: 63.1 mg (102.9 μmol, 82%). NMR data are consistent with that reported in the literature for complexes with different counterions.^9,18,44^

^**1**^ **H NMR** (400 MHz, DFB/Ar): δ 7.43 (t, ^3^ *J*_HH_ = 7.8, 1H, *p*-py), 7.29 (d, ^3^ *J*_HH_ = 7.8, 2H, *m*-py), 3.39 (vt, *J*_PH_ = 7.9, 4H, CH_2_), 1.20 (vt, *J*_PH_ = 14.4, 36H, *t*Bu).

^**31**^ **P­{** ^**1**^ **H} NMR** (162 MHz, DFB/Ar): δ 70.5 (d, ^1^ *J*_RhP_ = 126, 2P).

**HR ESI-MS** (positive ion, 4 kV): 526.1982 ([*M*]^+^, calcd 526.1982) *m*/*z*.

#### Step 2: Preparation of [Rh­(PNP*-*t*Bu)­(N_2_)] **5**

A suspension of [Rh­(PNP-*t*Bu)­(N_2_)]­BF_4_ (55.4 mg, 90.3 μmol) and KO*t*Bu (10.7 mg, 95.4 μmol) in hexane (15 mL) was stirred at RT for 24 h. The flask was sonicated for 2 min and the precipitate allowed to settle out. The neon red suspension was filtered, and the resulting red solution was blown down under a stream of N_2_ and dried *in vacuo* to afford **5** as a microcrystalline red solid. Yield: 33.1 mg (62.9 μmol, 70%). NMR data are consistent with the literature.^18^ Single crystals suitable for X-ray diffraction were obtained by crystallization from hexane at RT.

^**1**^ **H NMR** (400 MHz, C_6_D_12_/N_2_): δ 6.03–6.10 (m, 1H, *p*-py), 5.89 (d, ^3^ *J*_HH_ = 8.8, 1H, *m*-py), 5.16 (d, ^3^ *J*_HH_ = 6.3, 1H, *m*-py), 3.27 (d, ^2^ *J*_PH_ = 4.1, 1H, PCH), 2.82 (d, ^2^ *J*_PH_ = 8.5, 2H, CH_2_), 1.37 (d, ^3^ *J*_PH_ = 12.3, 18H, *t*Bu), 1.36 (d, ^3^ *J*_PH_ = 12.2, 18H, *t*Bu).

^**31**^ **P­{** ^**1**^ **H} NMR** (162 MHz, C_6_D_12_/N_2_): δ 66.6 (dd, ^2^ *J*_PP_ = 269, ^1^ *J*_RhP_ = 132, 1P), 63.1 (dd, ^2^ *J*_PP_ = 269, ^1^ *J*_RhP_ = 132, 1P).

### NMR Scale Reaction of [Rh­(PNP*-*t*Bu)­(N_2_)] 5 with HC≡C*t*Bu

A solution of **5** (5.3 mg, 10.0 μmol) and HC≡C*t*Bu (12.5 μL, 100 μmol) in C_6_D_12_ (0.5 mL) was prepared within a J. Young valve NMR tube and the ensuing reaction monitored at RT by NMR spectroscopy, with constant mixing when not in the spectrometer. A color change from red to deep red and quantitative conversion into [Rh­(PNP-*t*Bu)­(C≡C*t*Bu)] **4** (δ_31P_ 64.3) was observed within 22 h.

### Preparation and Characterization of [Rh­(PNP-*t*Bu)­(C≡C*t*Bu)] **4**

A solution of [Rh­(PNP*-*t*Bu)­(N_2_)] (70.8 mg, 135 μmol) and HC≡C*t*Bu (200 μL, 1.63 mmol) in cyclohexane (12 mL) was stirred at RT for 6 days, giving a deep red/brown solution. Volatiles were removed *in vacuo* to afford the product as a dark brown powder. Yield: 63.9 mg (110 μmol, 82%). Single crystals suitable for X-ray diffraction were obtained by crystallization from SiMe_4_ at −30 °C.

^**1**^ **H NMR** (500 MHz, C_6_D_12_): δ 7.25 (t, ^3^ *J*_HH_ = 7.6, 1H, *p*-py), 6.80 (d, ^3^ *J*_HH_ = 7.6, 2H, *m*-py), 3.02 (vt, *J*_PH_ = 6.4, 4H, CH_2_), 1.41 (vt, *J*_PH_ = 12.8, 36H, P*t*Bu), 1.11 (s, 9H, C≡C*t*Bu).

^**13**^ **C­{** ^**1**^ **H} NMR** (126 MHz, C_6_D_12_): δ 163.7 (app t, *J*_PC_ = 7, *o*-py), 132.1 (dt, ^2^ *J*_RhC_ = 15, ^3^ *J*_PC_ = 3, RhC≡C), 129.9 (s, *p*-py), 119.6 (app t, *J*_PC_ = 5, *m*-py), 96.1 (dt, ^1^ *J*_RhC_ = 48, ^2^ *J*_PC_ = 19, RhC), 38.2 (vt, *J*_PC_ = 8, CH_2_), 35.3 (vtd, *J*_PC_= 12, ^2^ *J*_RhC_ = 2, P*t*Bu­{C}), 33.7 (s, C≡C *t* Bu­{CH_3_}), 30.3 (br, C≡C *t* Bu­{C}), 30.1 (vt, *J*_PC_ = 8, P*t*Bu­{CH_3_}).

^**31**^ **P­{** ^**1**^ **H} NMR** (162 MHz, C_6_D_12_): δ 64.3 (d, ^1^ *J*_RhP_ = 155, 2P).

**HR ESI-MS** (positive ion, 4 kV): 602.2523 ([M+Na]^+^, calcd 602.2522) *m*/*z*.

### Preparation and Characterization of [Rh­(PNP-*t*Bu)­(C≡C*t*Bu)]­[BAr^F^_4_] **1**

A solution of [Rh­(PNP-*t*Bu)­(C≡C*t*Bu)] (75.0 mg, 129 μmol) and Fc­[BAr^F^_4_] (136 mg, 130 μmol) in DFB (4 mL) was stirred at RT for 2 h, giving a deep purple solution. Volatiles were removed *in vacuo* and the residue was washed with pentane (8 × 2 mL). Recrystallization from DFB/pentane at RT afforded the product as dark purple crystals, which were suitable for analysis by X-ray diffraction. Yield: 161.4 mg (112 μmol, 87%).

^**1**^ **H NMR** (400 MHz, 20 mM in DFB): δ 38.4 (vbr, FWHM = 800 Hz, 4H, CH_2_), 19.6 (vbr, FWHM = 610 Hz, 36H, P*t*Bu), 8.08–8.14 (m, 8H, Ar^F^), 7.42 (br, 4H, Ar^F^), 1.43 (vbr, FWHM = 100 Hz, 2H, *m*-py), −5.35 (vbr, FWHM = 30 Hz, 9H, C≡C*t*Bu), −8.85 (vbr, FWHM = 70 Hz, 1H, *p*-py).

^**31**^ **P­{** ^**1**^ **H} NMR** (162 MHz, 20 mM in DFB): no resonances observed between δ −1000 and +1000.

**μ**_**eff**_ (Evans’ method): 1.74 μ_B_

**HR ESI-MS** (positive ion, 4 kV): 579.2637 ([M]^+^, calcd 579.2625) *m*/*z*.

### Characterization of [Rh­(PNP-*t*Bu)­(C≡C*t*Bu)]­[BAr^F^_4_] 1 by EPR Spectroscopy

#### Solid State

In an argon glovebox, **1** (3.6 mg, 2.50 μmol) and [*n*Bu_4_N]­[BAr^F^_4_] (36.0 mg, 32.6 μmol) were ground together in a pestle and mortar. The resulting solid mixture was loaded into a J. Young valve quartz EPR tube, placed under high vacuum and then flame-sealed.

#### Frozen Glasses

After confirming solution stability of **1** in the given solvent (10 mM) for 24 h at RT by ^1^H NMR spectroscopy, a fresh solution of **1** (3.6 mg, 2.50 μmol) in a mixture of the solvent (200 μL) and cyclohexane (50 μL) was prepared within a J. Young valve EPR tube, frozen and then analyzed by EPR spectroscopy at 115 K. Only minor perturbations are observed for **1** in the different mixtures. The similarity of the parameters of **1**·Solvent points to common structure (Table 1).

**Table 1:** Spin Hamiltonian Parameters for **1** and Corresponding Solvent Adducts^a^

Medium	Species	*g*_1_	*g*_2_	*g*_3_	A_1_/MHz	A_2_/MHz	A_3_/MHz	**1**:**1**·Solvent
Solid state	**1**	4.051	1.328	1.140				n/a
THF:CyH	**1**	3.941	1.451	1.281	300			1:4
	**1**·THF	2.646	2.362	1.940	111	132	141	
DFB:CyH	**1**	3.982	1.286	1.274	307			1:1.25
	**1**·DFB	2.441	2.301	1.970	102	103	113	
FB:CyH	**1**	4.001	1.321	1.221	310			1:0.40
	**1**·FB	2.438	2.291	1.970	119	128	118	
MeTHF: CyH	**1**	3.971	1.510	1.275	300			1:0.40
	**1**·MeTHF	2.438	2.298	1.970	109	103	113	
TFT:CyH	**1**	4.010	1.320	1.210	308			1:0.33
	**1**·TFT	2.440	2.301	1.971	102	97	113	

a Values of *g* reported to ± 0.001 and values of A reported to ± 15 MHz (**1**) and ± 5 MHz (**1**·Solvent) for frozen glass samples, when resolved. The **g**-frame and **A**-frame are assumed to be coincident.

### Reaction with 9,10-Dihydroanthracene

#### General Procedure

A solution of **1** (7.2 mg, 4.99 μmol) and 9,10-dihydroanthracene (9.0 mg, 49.9 μmol) in a given solvent (0.5 mL) was prepared in a J. Young valve NMR tube, heated at 60 °C and the ensuing reaction monitored every 24 h by NMR spectroscopy at RT. Over time a color change from purple to malachite green was observed upon conversion of **1** into [Rh­(PNP-*t*Bu)­(C=CH*t*Bu)]­[BAr^F^_4_] **2** (δ_31P_ 65.6 in DFB) and half an equivalent of anthracene.

#### Isolation of [Rh­(PNP-*t*Bu)­(C=CH*t*Bu)]­[BAr^F^_4_] **2**

A solution of **1** (14.4 mg, 9.98 μmol) and 9,10-dihydroanthracene (18.0 mg, 99.9 μmol) in DFB (0.5 mL) was prepared in a J. Young valve NMR tube and heated for 14 days at 60 °C. Volatiles were removed under vacuum and the resulting oily green solid was washed with toluene to give the product as a dark green microcrystalline solid. Yield: 11.3 mg (7.83 μmol, 78%). NMR data are consistent with the literature.^9^

^**1**^ **H NMR** (400 MHz, CD_2_Cl_2_): δ 7.74 (t, ^3^ *J*_HH_ = 8.0, 1H, *p*-py), 7.69–7.74 (m, 8H, Ar^F^), 7.55 (br, 4H, Ar^F^), 7.42 (d, ^3^ *J*_HH_ = 8.0, 2H, *m*-py), 3.72 (br, 4H, CH_2_), 1.40 (vt, *J*_PH_ = 13.5, 36H, P*t*Bu), 1.4 (obscured, 1H, C=CH *t*=Bu), 1.15 (s, 9H, C=CH *t* Bu).

^**31**^ **P­{** ^**1**^ **H} NMR** (162 MHz, CD_2_Cl_2_): δ 65.7 (d, ^1^ *J*_RhP_ = 139, 2P).

^**31**^ **P­{** ^**1**^ **H} NMR** (162 MHz, DFB): δ 65.6 (d, ^1^ *J*_RhP_ = 138, 2P).

^**31**^ **P­{** ^**1**^ **H} NMR** (162 MHz, CD_3_CN): δ 66.7 (d, ^1^ *J*_RhP_ = 139, 2P).

### Reaction of [Rh­(PNP-*t*Bu)­(C=CH*t*Bu)]­[BAr^F^_4_] 2 with CD_3_CN

A solution of **2** (14.4 mg, 9.97 μmol) in CD_3_CN (0.5 mL) was prepared in a J. Young valve NMR tube resulting in equilibrium formation of [Rh­(PNP-*t*Bu)­H­(C≡C*t*Bu)­(NCMe)]­[BAr^F^_4_] **3**·ACN, which was characterized *in situ* by multinuclear NMR spectroscopy. Van’t Hoff analysis of the equilibrium between 273 and 343 K using ^1^H NMR spectroscopy enabled the thermodynamic parameters to be determined (*K* = [**3**·ACN]/[**2**]­[CD_3_CN], where [CD_3_CN] = 19.1 M; Δ*H* = −7.81 ± 0.09 kcal·mol^–1^, Δ*S =* −30.5 ± 0.3 cal·mol^–1^K^–1^, Δ*G*_298K_ = +1.3 ± 0.2 kcal·mol^–1^).

#### Characterization Data for **3**·ACN

^**1**^

**H NMR** (500 MHz, CD_3_CN, selected data): δ 7.73 (t, ^3^ *J*_HH_ = 7.9, 1H, *p*-py), 7.40 (d, ^3^ *J*_HH_ = 7.9, 2H, *m*-py), 3.78 (d, ^2^ *J*_HH_ = 16.8, 2H, CH_2_), 3.66 (d, ^2^ *J*_HH_ = 16.8, 2H, CH_2_), 1.48 (vt, *J*_PH_ = 14.1, 18H, P*t*Bu), 1.29 (vt, *J*_PH_ = 13.4, 18H, P*t*Bu), 1.10 (s, 9H, C≡C*t*Bu), −16.66 (dt, ^1^ *J*_RhH_ = 19.8, ^2^ *J*_PH_ = 10.9, 1H, Rh–H).

^**13**^ **C­{** ^**1**^ **H} NMR** (126 MHz, CD_3_CN, selected data) δ 163.8 (vt, *J*_PC_ = 7, *o*-py), 139.7 (s, *p*-py), 122.2 (vt, *J*_PC_ = 9, *m*-py), 116.23 (dt, ^2^ *J*_RhC_ = 10, ^3^ *J*_PC_ = 3, RhC≡C), 72.7 (dt, ^1^ *J*_RhC_ = 42, ^2^ *J*_PC_ = 16, RhC), 37.7 (vt, *J*_PC_ = 15, CH_2_), 36.7 (vtd, *J*_PC_ = 16, ^2^ *J*_RhC_ = 2, P*t*Bu­{C}), 33.1 (s, C≡C*t*Bu­{CH_3_}), 30 (obscured, C≡C*t*Bu­{C}), 29.8 (vt, *J*_PC_ = 6, P*t*Bu­{CH_3_}).

^**31**^ **P­{** ^**1**^ **H} NMR** (162 MHz, CD_3_CN): δ 71.5 (dd, ^1^ *J*_RhP_ = 101, 2P).

### Computational Details

All electronic structure calculations pertaining to mechanistic aspects presented in this paper were carried out using the Gaussian 16 (Revision B.01)^45^ program suite at the DFT level of theory. Geometries of all compounds were fully optimized without imposing symmetry constraints (*C*_1_ symmetry), employing the Minnsesota M06–L local meta-generalized gradient approximation (meta-GGA) functional.^27a,27b^ The Stuttgart-Dresden (SDD)^27c^ relativistic effective core potential in combination with the associated basis sets were used to describe the Rh and P centers, augmented with an additional f-type (Rh, ζ = 1.350) or d-type (P, ζ = 0.387) polarization function.^27d,27e^ The 6–31G­(d,p) basis sets^27f,27g^ developed by Pople and co-workers were used on all lighter atoms (C, N, and H). Optimized stationary points were characterized by analysis of their analytical second derivatives, with minima having only positive eigenvalues and transition states having exactly one imaginary eigenvalue. The nature of transition states was confirmed via intrinsic reaction coordinate (IRC) calculations in both forward and reverse direction of the imaginary mode displacement vector.^27h^ Subsequent geometry optimizations of the IRC end points were used to identify the nearest minima linked by a transition state. The frequency calculations also provided thermal and entropic corrections to the total energy in gas phase at *T* = 298.15 K and *p* = 1 atm within the rigid-rotor/harmonic oscillator (RRHO) approximation. Dispersion effects were accounted for by applying Grimme’s van der Waals correction with D3 parametrization^27i^ during geometry optimizations of all stationary points. Effects due to the presence of a solvent were treated implicitly with a polarizable dielectric model, using the IEFPCM formalism in conjunction with Truhlar’s SMD model.^27j^ For cyclohexane solvent (ε = 2.0), default parameters were used. In the absence of defined parameters for 1,2-difluorobenzene (DFB) solvent, default SMD parameters were selected for fluorobenzene and the dielectric constant adjusted to that of DFB (ε = 13.8).^27k^ Corrections for standard state (−*RT *ln­(24.5) = −1.9 kcal·mol^–1^) and excess solvent (−*RT *ln* Q* = −4.1 kcal·mol^–1^ based on [CH_3_CN]/[Rh] ∼1000) was applied to the Gibbs Free Energy of the reaction **2** + CH_3_CN → **3**·ACN). Bond dissociation energies (*D*_e_) were calculated as the gas-phase enthalpy change at 298 K using X–H → X^•^ + H^•^, *D*_e_ = *H*_298K_(X^•^) + *H*_298K_(H^•^) – H_298K_(X–H). Bond dissociation free energies were calculated following reported literature procedures and assuming a Δ*G°* of 52 kcal mol^–1^ to convert 1/2 H_2_ (g) to H_1M_^•^ in DFB.^46^ Additional single point SCF calculations employed the M06-D3 functional^27l^ in conjunction with the SDD effective core potential and associated basis set on Rh, and the 6–311++G­(2d,2p) basis set on remaining atoms. All structures were visualized using the Chemcraft software.^47^

Calculations relating to EPR *g*-factors of **1** and **1**·DFB were performed with ORCA 6.0.1.^22a^ In addition to structures generated as described above, unconstrained geometry optimizations and analytical frequency calculations were carried out with the PBE0 hybrid GGA functional,^48^ in conjunction with the RIJCOSX approximation.^49^ The def2-TZVP basis set was used on all atoms (including the corresponding def2-ECP for Rh),^50^ in conjunction with the AutoAux feature to generate matching auxiliary basis sets.^51^ Dispersion effects were accounted for by Grimme’s atom-pairwise D4 correction.^52^ Subsequent single point calculations were performed to evaluate g-values at the CASSCF and NEVPT2 levels of theory.^22b–22f^ The active CAS­(9,6) space contains 9 electrons in 6 orbitals, comprised of five orbitals of 4d character located on Rh and one σ-bonding ligand orbital involving all ligand donor atoms. Starting orbitals for the targeted active space were generated with the Atomic Valence Active Space (AVAS) procedure.^22g^ Scalar relativistic effects were included via the exact two-component x2C Hamiltonian,^22h^ in conjunction with the all-electron Karlsruhe x2c-TZVP basis set on all atoms.^22i^ The effect of spin–orbit coupling was explicitly included. The AutoAux feature was used to generate matching auxiliary basis sets. The **g**-tensors were calculated using the quasi-degenerate perturbation theory approach implemented in ORCA.^22j^

## Supplementary Material





## Notes

NMR, EPR, and ESI-MS spectra of complexes and selected reactions; additional electrochemical data and analysis; and computational data and analysis (PDF)
Optimized geometries of species examined computationally (XYZ)
