# Supplementary material for: Solvent-Dependent Carbon-to-Metal Hydrogen Atom Transfer Reactivity of a Square Planar Rhodium(II) Alkynyl Complex
Source: Organometallics. 2026 Apr 1;45(7):892–901. doi: 10.1021/acs.organomet.6c00053 (PMC13081109; doi:10.1021/acs.organomet.6c00053)
Supplement: Supplementary file 1 [file om6c00053_si_001.pdf]

Supporting information for:

Solvent dependent carbon-to-metal hydrogen atom transfer reactivity of a square planar rhodium(II) alkynyl complex

Thomas M. Hood,[a] Sophie H. Dewick,[a] Anjali John,[a] Jeremiah P. Tidey,[b] Julie V. Macpherson,[a] Ragnar Bjornsson,[c] Emma Richards,[d] Tobias Krämer,[e] and Adrian B. Chaplin[a]

[a] *Department of Chemistry, University of Warwick, Coventry CV4 7AL, UK.* [b] *Department of Physics, University of Warwick, Coventry CV4 7AL, UK.* [c] *Laboratoire de Chimie et Biologie des Métaux, Université Grenoble Alpes, CNRS, CEA, IRIG, F-38054 Grenoble, Cedex, France.*
[d] *School of Chemistry, Cardiff University, Translational Research Hub, Cardiff CF24 4HQ, UK.*
[e] *School of Chemistry, Trinity College Dublin, The University of Dublin, Dublin 2, Ireland.*

Table of contents

1. Characterisation of [Rh(PNP-*t*Bu)Cl] and [Rh(PNP-*t*Bu)(CH_3)] 2
2. Preparation and characterisation of [Rh(PNP*-*t*Bu)(N_2)] 5 4
3. NMR scale reaction of [Rh(PNP*-*t*Bu)(N_2)] 5 with HC≡C*t*Bu 6
4. Preparation and characterisation of [Rh(PNP-*t*Bu)(C≡C*t*Bu)] 4 7
5. Preparation and characterisation of [Rh(PNP-*t*Bu)(C≡C*t*Bu)][BAr$^F_4$] 1 9
6. Characterisation of [Rh(PNP-*t*Bu)(C≡C*t*Bu)][BAr$^F_4$] 1 by EPR spectroscopy 11
6.1. Solid state 11
6.2. DFB glass 12
6.3. FB glass 13
6.4. TFT glass 14
6.5. THF glass 15
6.6. MeTHF glass 16
7. Reaction of [Rh(PNP-*t*Bu)(C≡C*t*Bu)][BAr$^F_4$] 1 with 9,10-dihydroanthracene 17
7.1. Reaction in DFB 17
7.2. Reaction in FB 20
7.3. Reaction in TFT 21
7.4. Reaction in THF 22
7.5. Reaction in MeTHF 23
7.6. Isolation of [Rh(PNP-tBu)(C=CHtBu)][BAr$^F_4$] ***2*** 24
8. Reaction of [Rh(PNP-*t*Bu)(C=CH*t*Bu)][BAr$^F_4$] 2 with CD_3CN 25
9. Computational details 29
9.1. Molecular orbital diagram of ***1*** 29
9.2. Reaction profiles 30
9.3. Structures and energetics of ***1***·*Solvent and* ***3***·*ACN* 35
9.4. Multireference CAS(9,6)/NEVPT2 calculations 36

1. Characterisation of [Rh(PNP-*t*Bu)Cl] and [Rh(PNP-*t*Bu)(CH_3)]

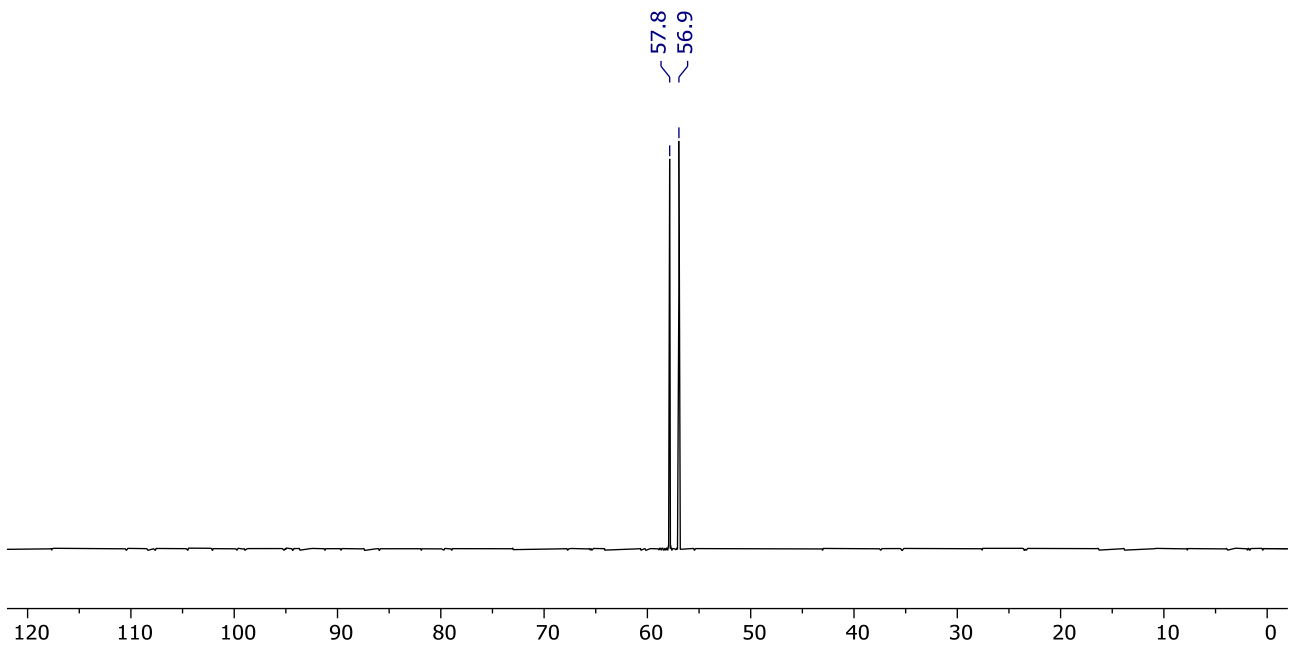

Figure S1. $^{31}P\{^{1}H\}$ NMR spectrum of [Rh(PNP-*t*Bu)Cl] (162 MHz, C_6D_6).

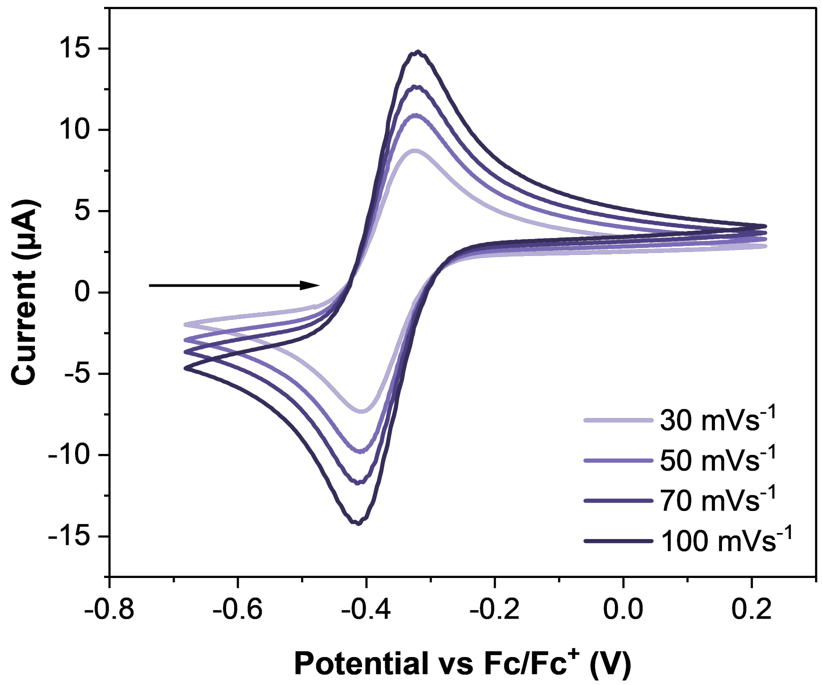

Figure S2. Cyclic voltammogram for the oxidation of [Rh(PNP-*t*Bu)Cl] ($E_{1/2}$ = –0.37 V vs Fc^+/Fc, i_p^{red}/i_p^{ox} = 0.91) in DFB at RT (2 mM complex; 0.2 M [*n*Bu_4N][$BAr^F{}_4$] electrolyte; glassy carbon working electrode, coiled Pt wire counter electrode, and Ag wire quasi-reference electrode).

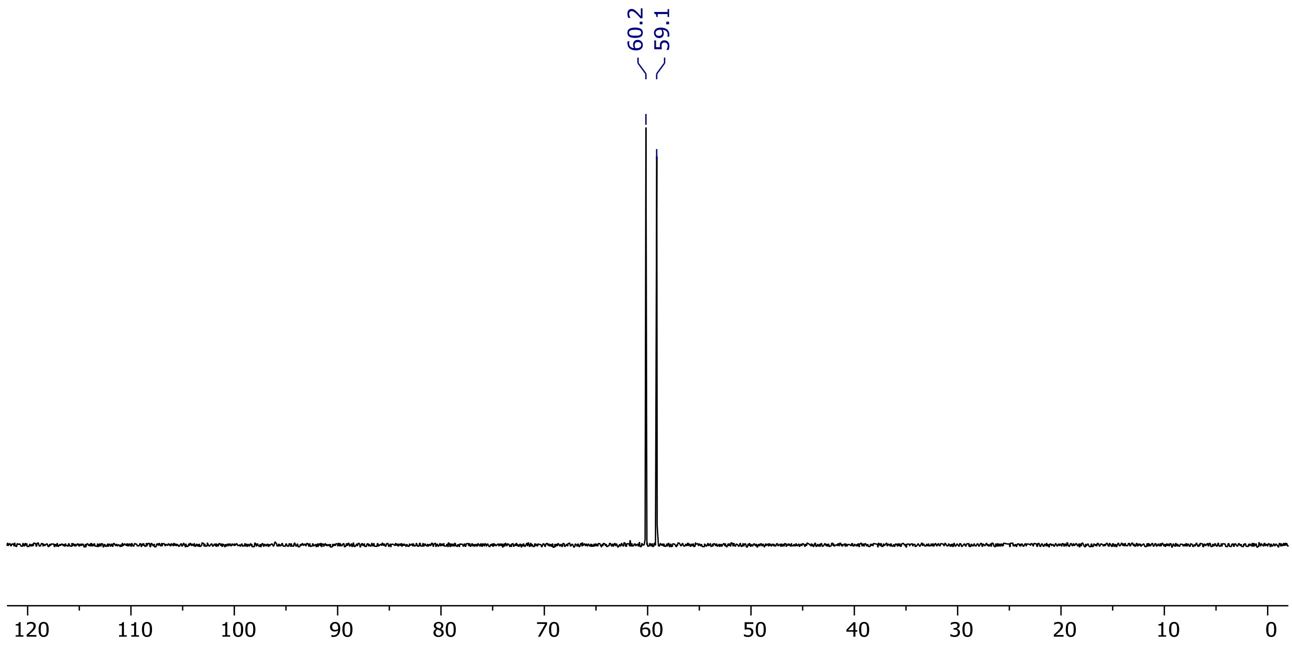

Figure S3. $^{31}P\{^{1}H\}$ NMR spectrum of [Rh(PNP-*t*Bu)(CH_3)] (162 MHz, THF).

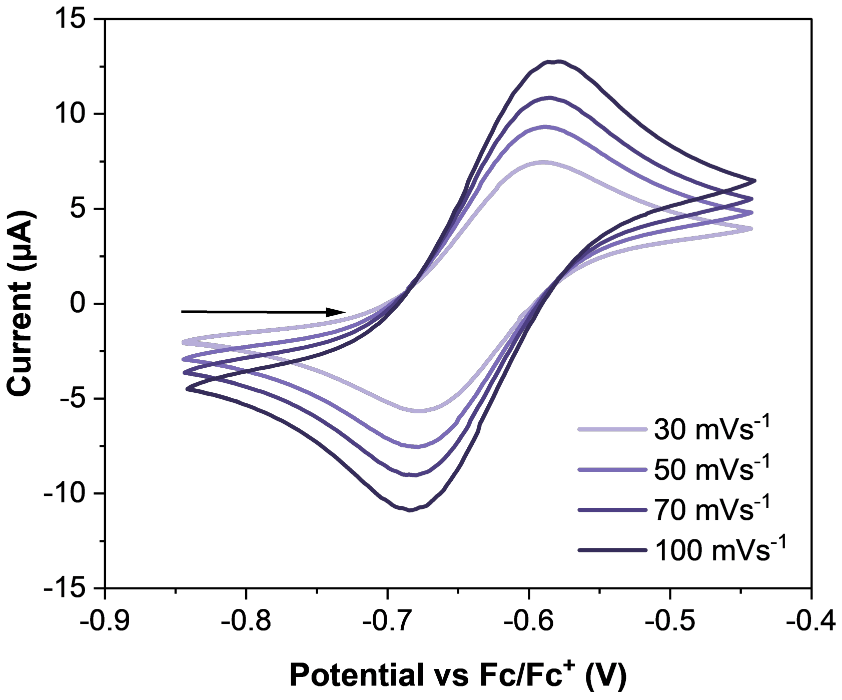

Figure S4. Cyclic voltammogram for the oxidation of [Rh(PNP-*t*Bu)(CH_3)] ($E_{1/2}$ = –0.63 V vs Fc^{+}/Fc, i_p^{red}/i_p^{ox} = 0.93) in DFB at RT (2 mM complex; 0.2 M [*n*Bu_4N][$BAr^F{}_4$] electrolyte; glassy carbon working electrode, coiled Pt wire counter electrode, and Ag wire quasi-reference electrode).

2. Preparation and characterisation of [Rh(PNP*-*t*Bu)(N_2)] 5

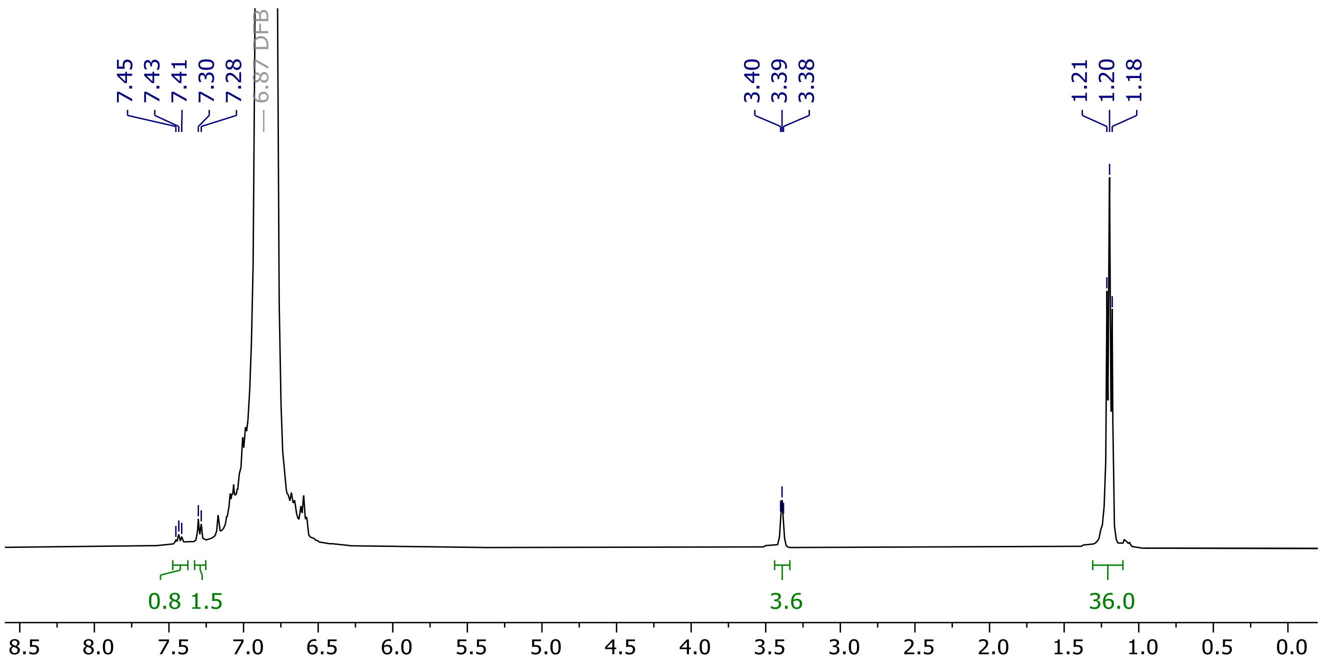

Figure S5. ^{1}H NMR spectrum of [Rh(PNP-*t*Bu)(N_2)]BF_4 (400 MHz, DFB/Ar).

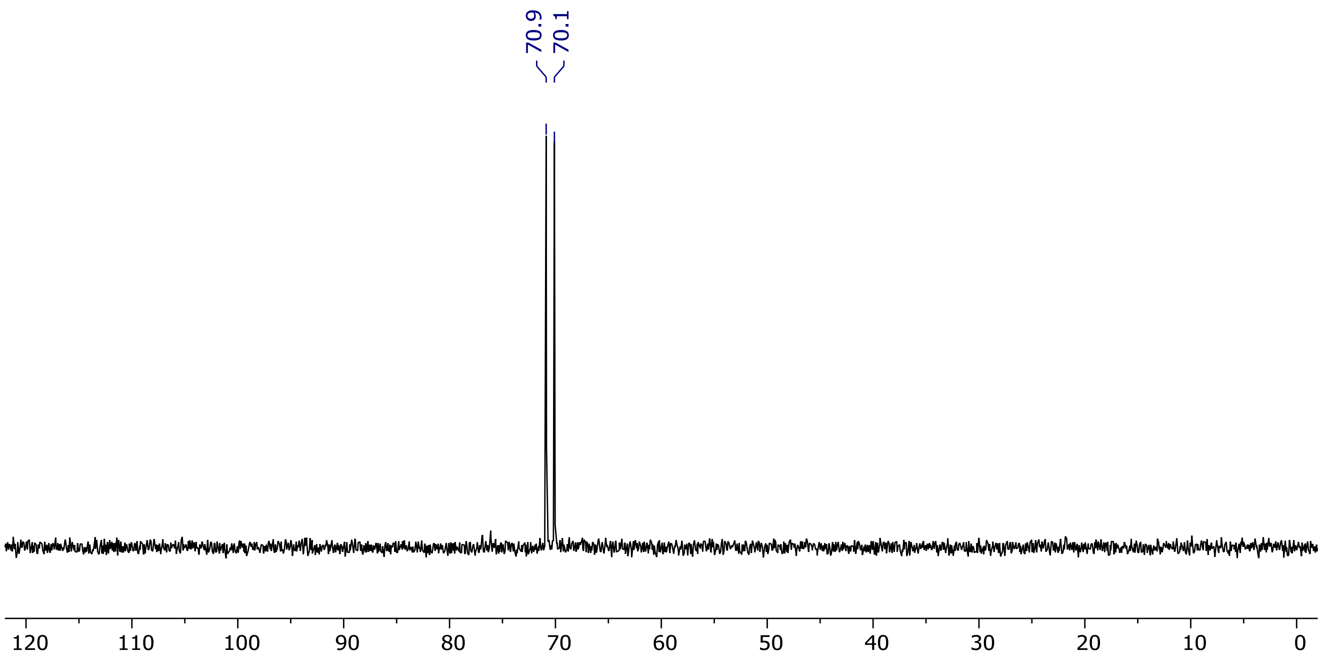

Figure S6. ^{31}P{^{1}H} NMR spectrum of [Rh(PNP-*t*Bu)(N_2)]BF_4 (162 MHz, DFB/Ar).

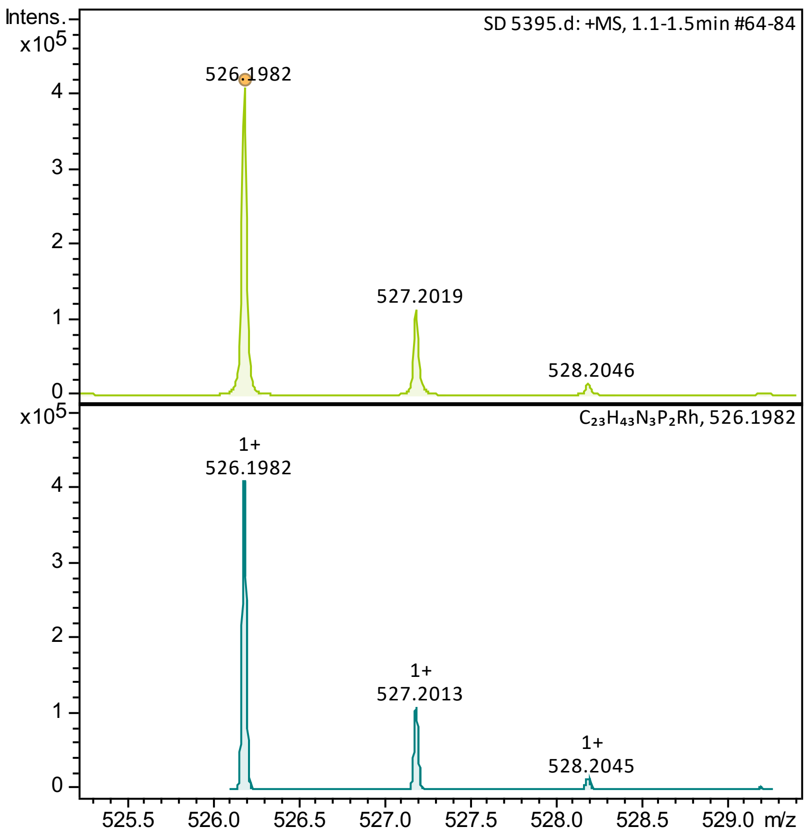

Figure S7. HR ESI-MS of [Rh(PNP-*t*Bu)(N_2)]BF_4.

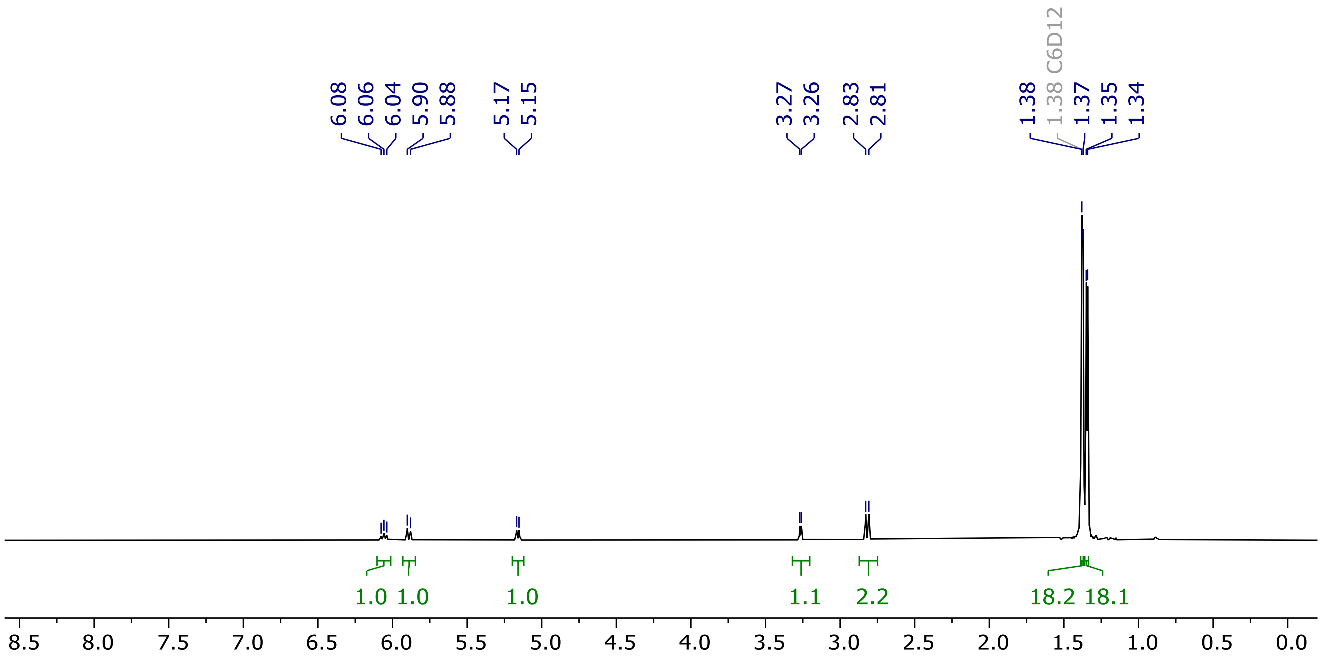

Figure S8. ^{1}H NMR spectrum of **5** (400 MHz, C_6D_{12}/N_2).

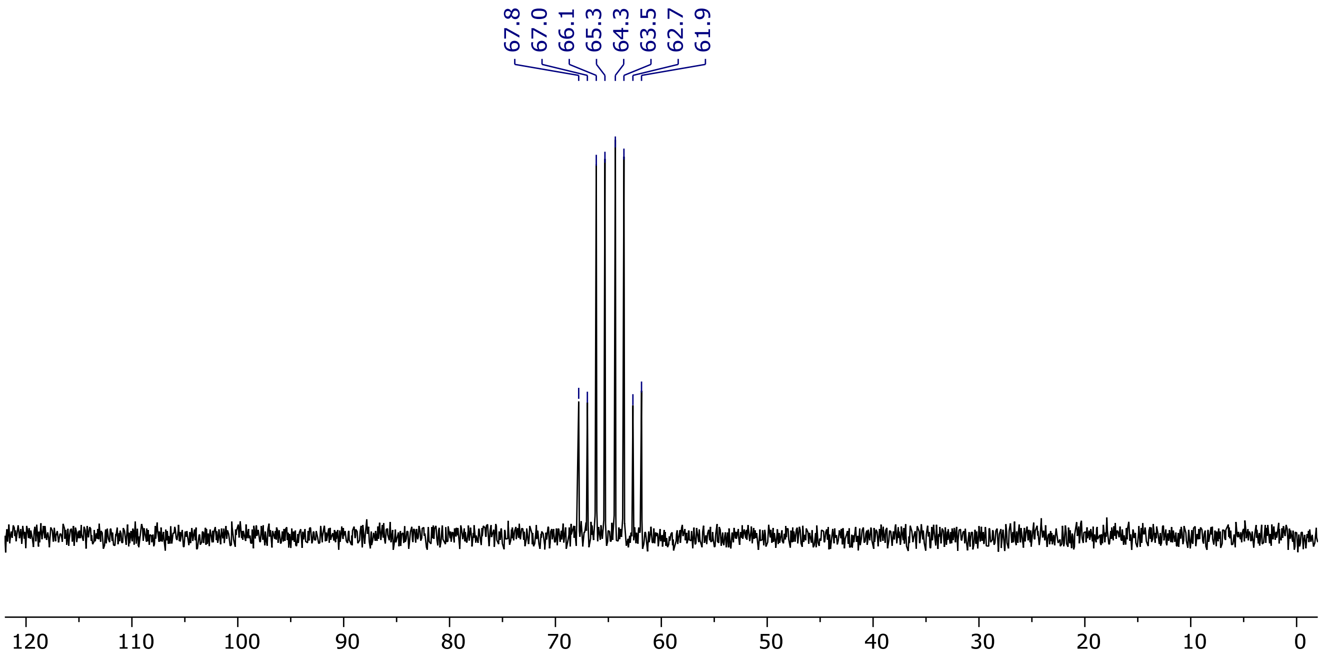

Figure S9. $^{31}P\{^{1}H\}$ NMR spectrum of **5** (162 MHz, C_6D_{12}/N_2).

3. NMR scale reaction of [Rh(PNP*-*t*Bu)(N_2)] 5 with HC≡C*t*Bu

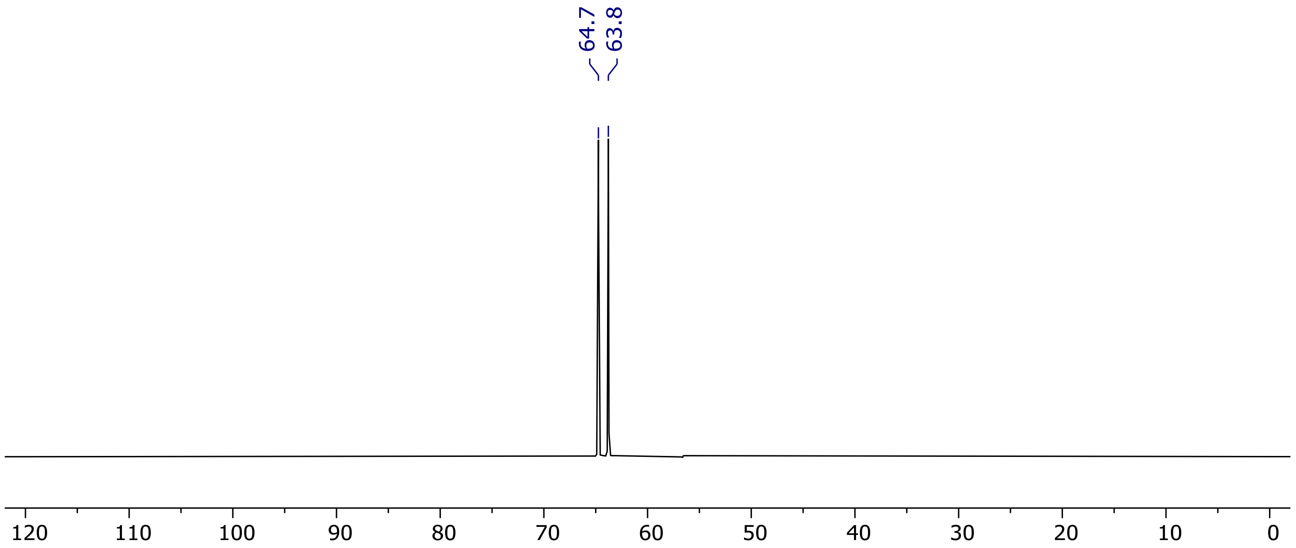

Figure S10. $^{31}P\{^{1}H\}$ NMR spectrum of the reaction between **5** and HC≡C*t*Bu recorded after 22 h at RT (162 MHz, C_6D_{12}).

4. Preparation and characterisation of [Rh(PNP-*t*Bu)(C≡C*t*Bu)] 4

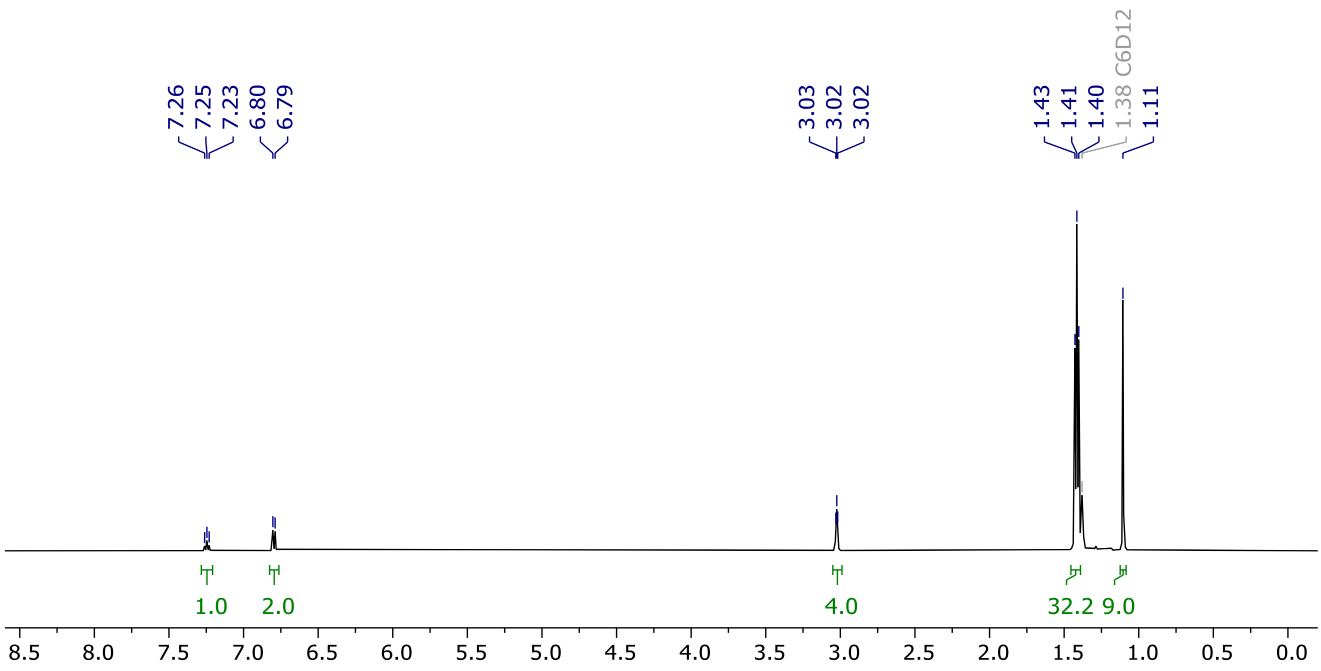

Figure S11. ^{1}H NMR spectrum of **4** (500 MHz, C_6D_{12}).

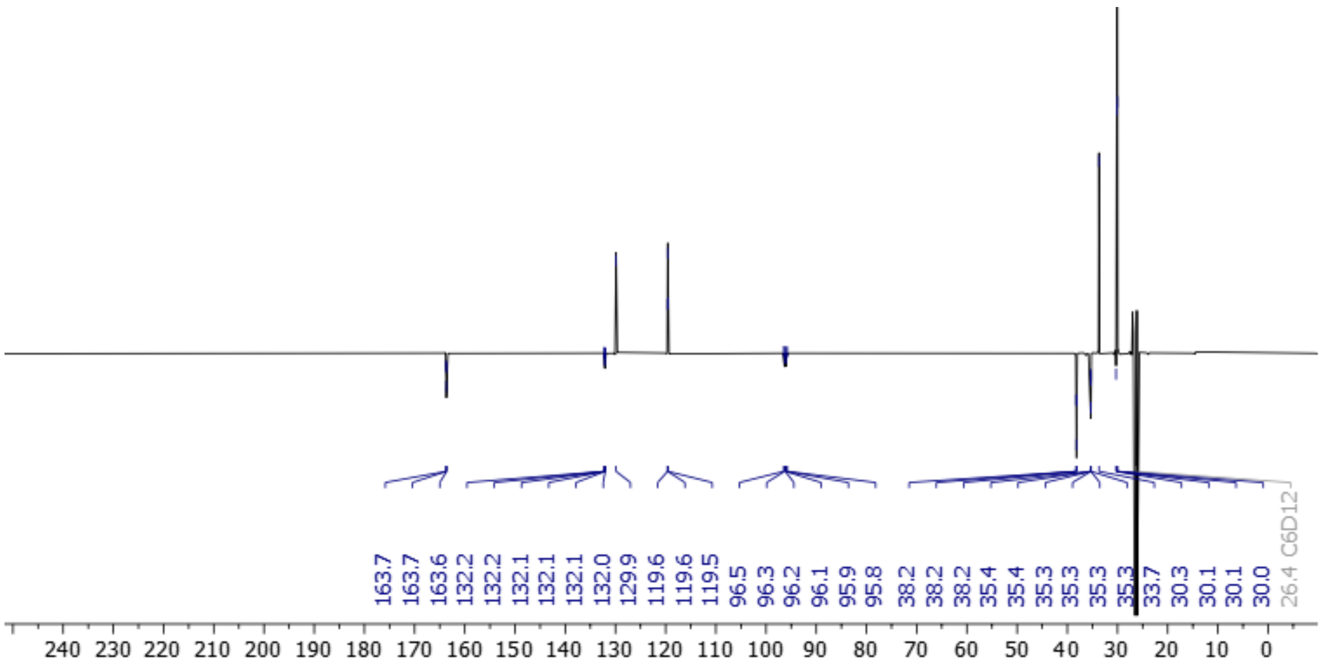

Figure S12. $^{13}C\{^{1}H\}$ NMR spectrum of **4** (126 MHz, C_6D_{12}).

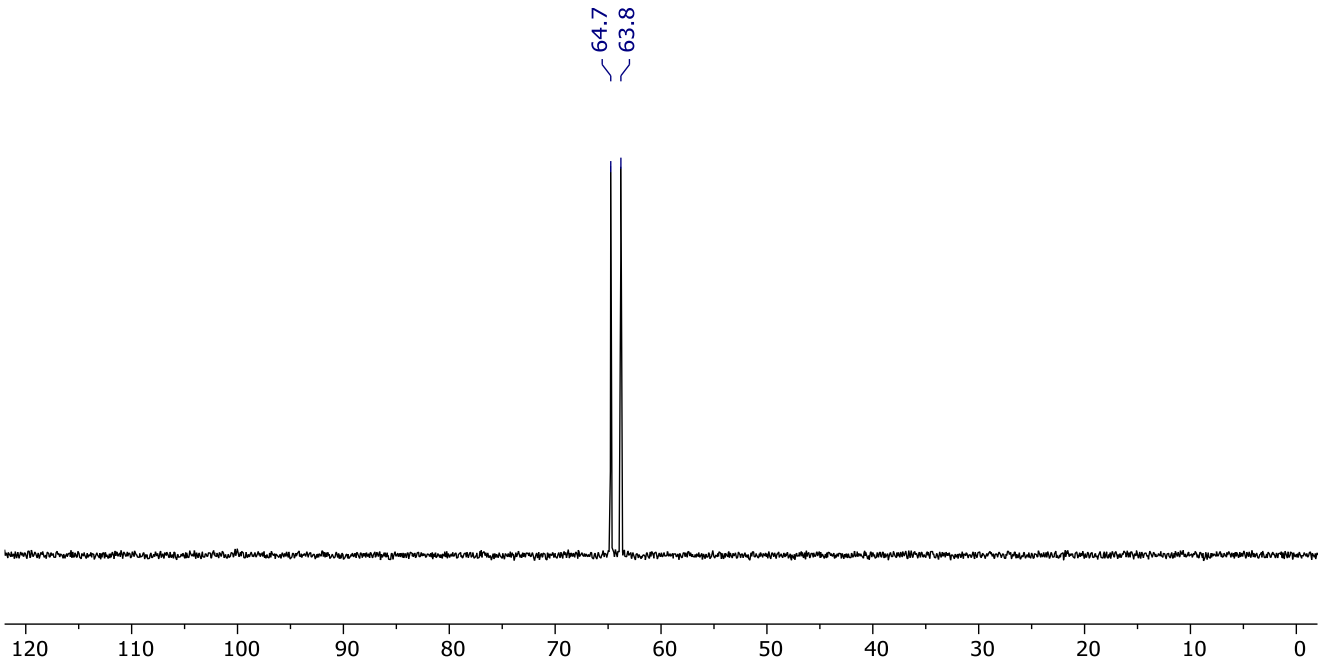

Figure S13. $^{31}P\{^{1}H\}$ NMR spectrum of **4** (162 MHz, C_6D_{12}).

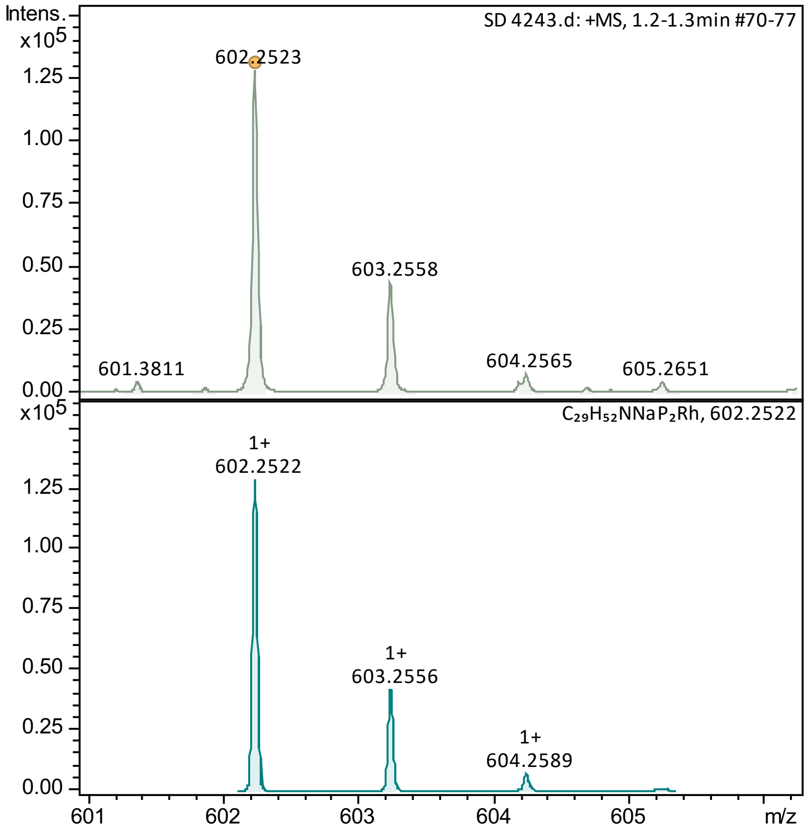

Figure S14. HR ESI-MS of **4**.

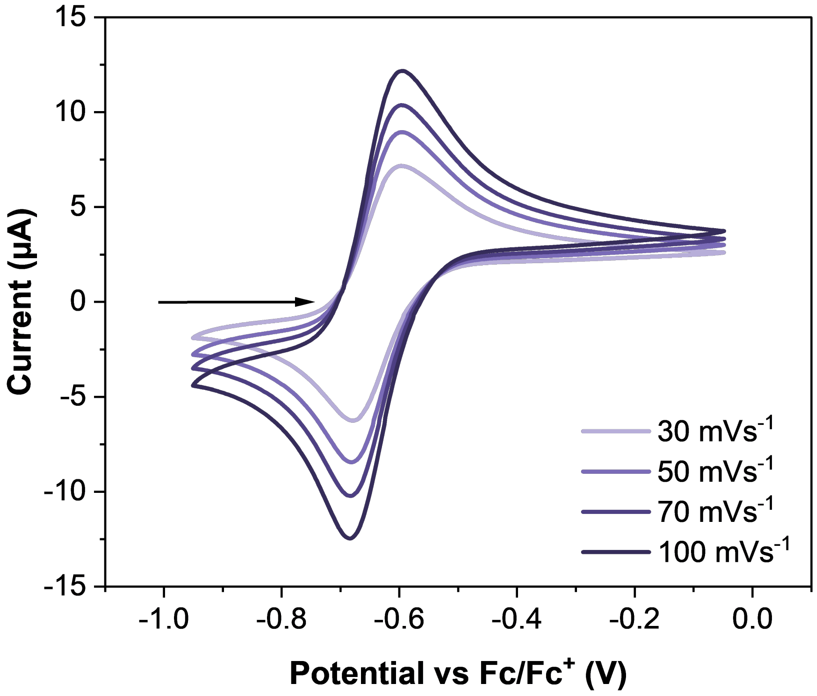

Figure S15. Cyclic voltammograms for the oxidation of **4** ($E_{1/2}$ = –0.64 V vs Fc^+/Fc, i_p^{red}/i_p^{ox} = 0.99) in DFB at RT (2 mM complex; 0.2 M [nBu$_4$N][BAr$^F{}_4$] electrolyte; glassy carbon working electrode, coiled Pt wire counter electrode, and Ag wire quasi-reference electrode).

5. Preparation and characterisation of [Rh(PNP-*t*Bu)(C≡C*t*Bu)][BAr$^F{}_4$] 1

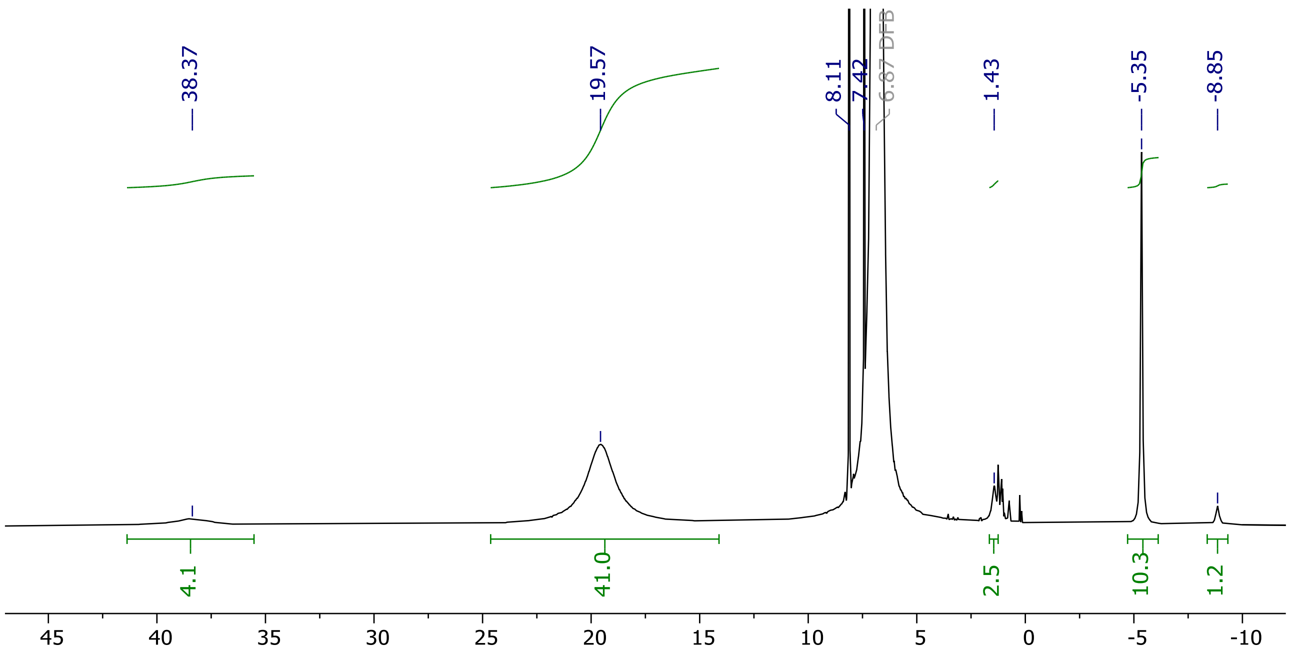

Figure S16. ^{1}H NMR spectrum of **1** (400 MHz, 20 mM in DFB).

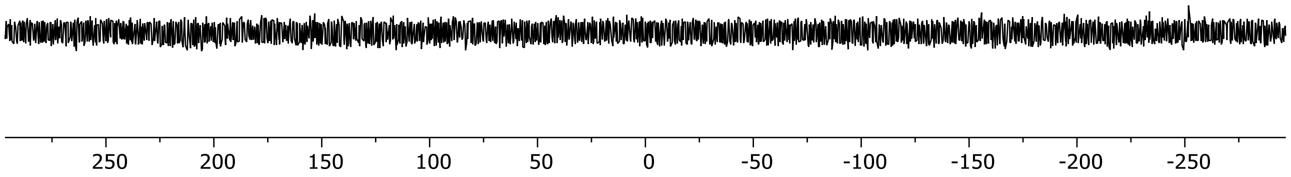

Figure S17. $^{31}P\{^1H\}$ NMR spectrum of **1** between *δ* −300 and +300 (162 MHz, 20 mM in DFB).

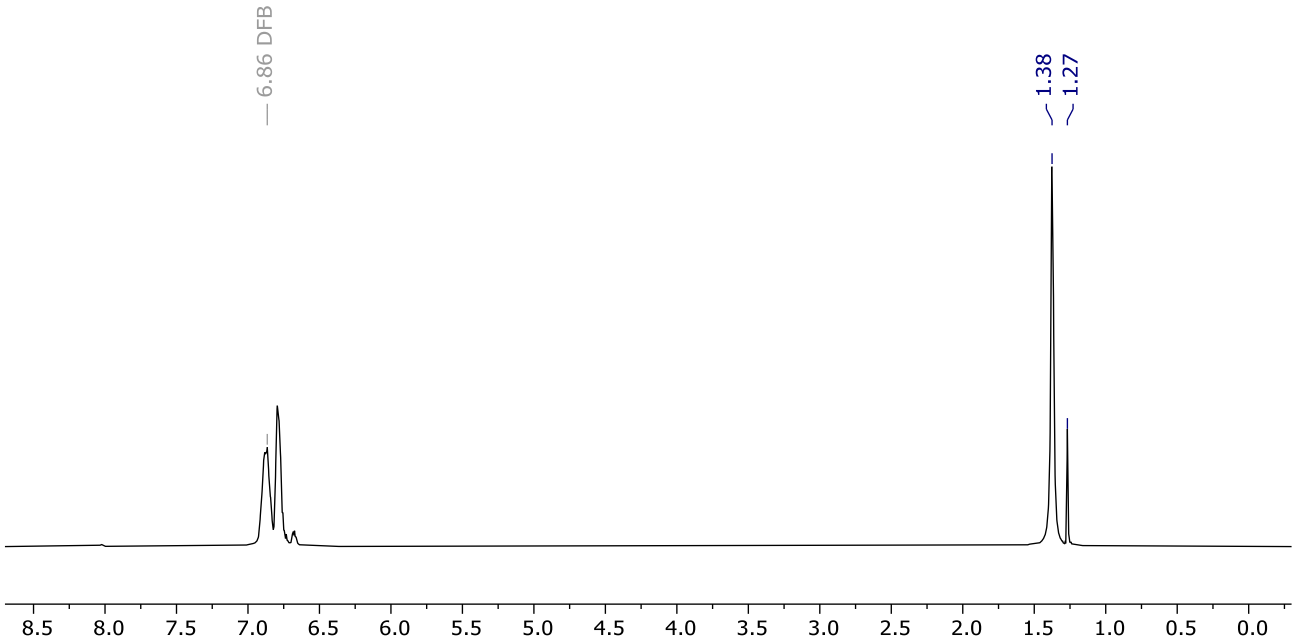

Figure S18. ^1H NMR spectrum of **1** (7.3 mg, 5.06 μmol) in a 3:1:1 v/v solution of DFB/C_6D_6/CyH (400 μL) with an internal capillary of DFB/C_6D_6/CyH (400 MHz). CyH paramagnetic shift = 43 Hz.

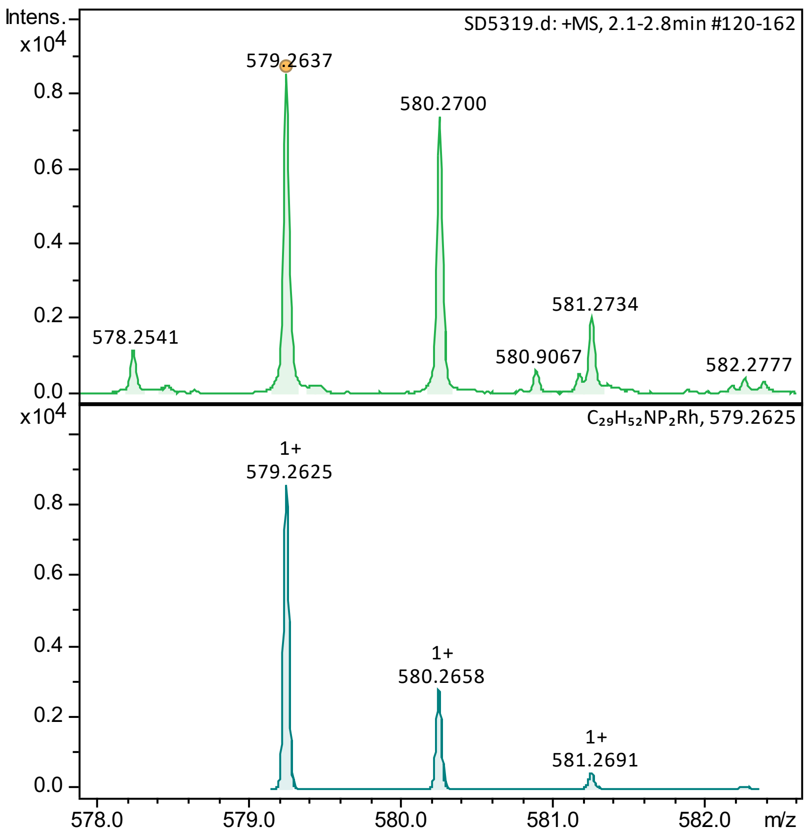

Figure S19. HR ESI-MS of **1**.

6. Characterisation of [Rh(PNP-*t*Bu)(C≡C*t*Bu)][BAr$^F{}_4$] 1 by EPR spectroscopy

6.1. Solid state

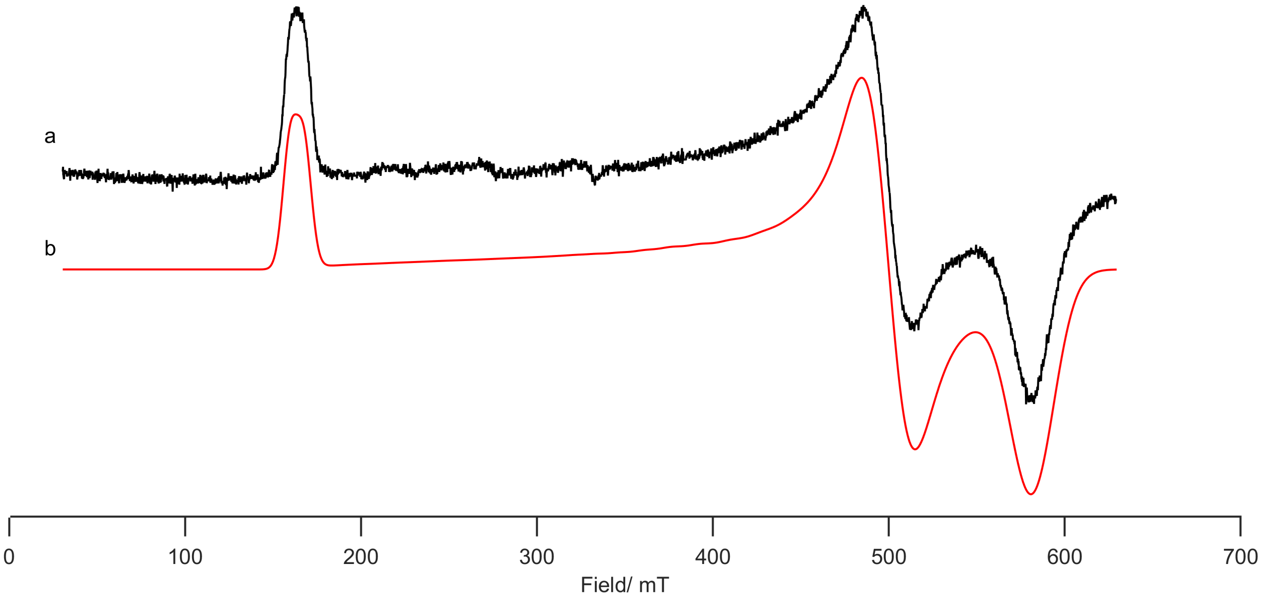

Figure S20. Experimental (a) and simulated (b) CW X-band EPR spectra of **1** recorded in the solid state at 115 K (1:10 w/w dispersion of powdered sample in [*n*Bu$_4$N][BAr$^F{}_4$]).

6.2. DFB glass

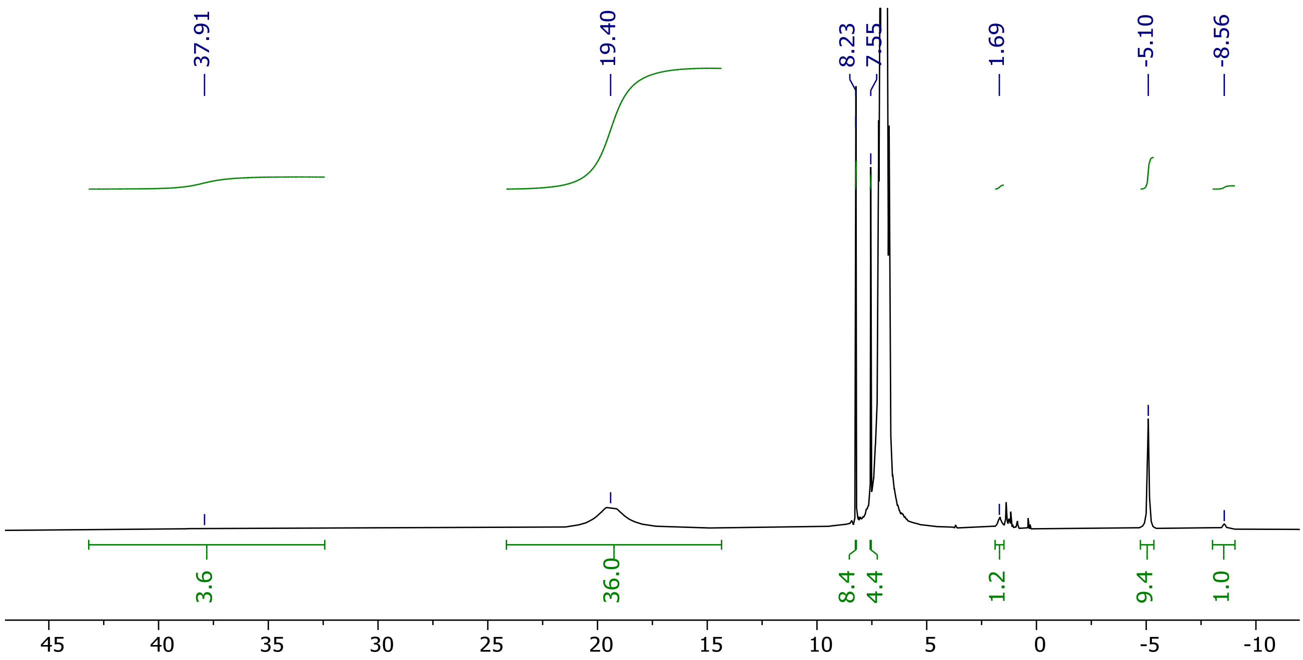

Figure S21. ^{1}H NMR spectrum of **1** (10 mM) recorded in DFB after 24 h at RT (400 MHz).

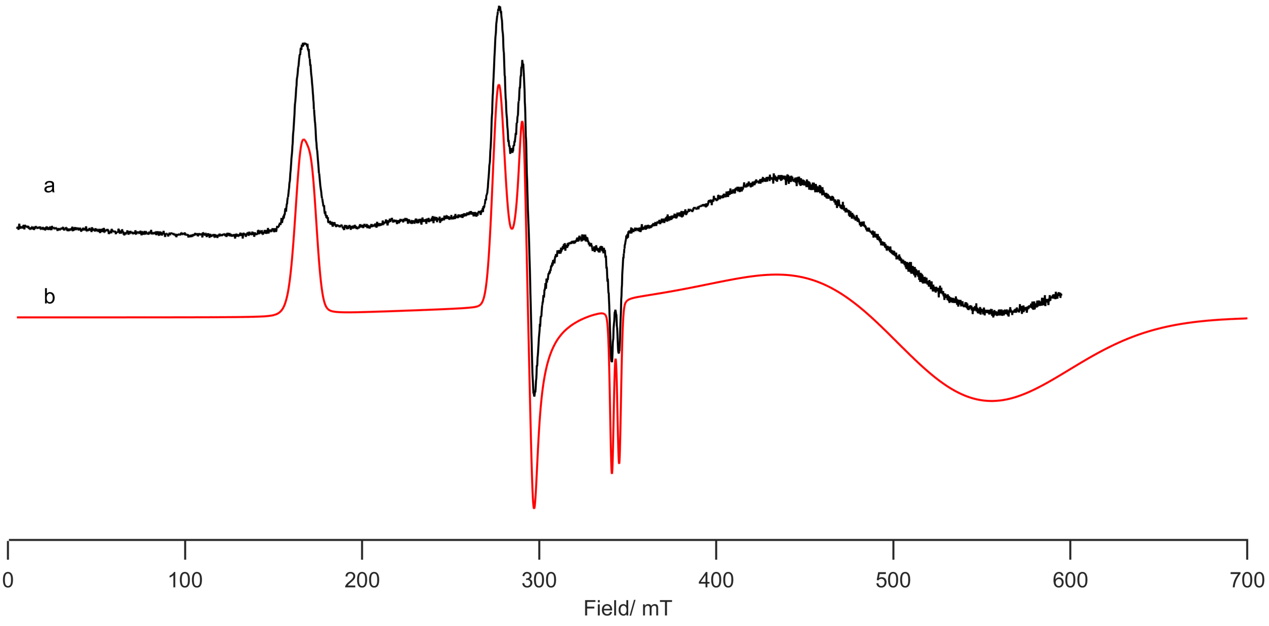

Figure S22. Experimental (a) and simulated (b) CW X-band EPR spectra of **1** recorded in 4:1 v/v DFB/cyclohexane frozen glass at 115 K. **1**:**1**·DFB ratio = 1:1.25.

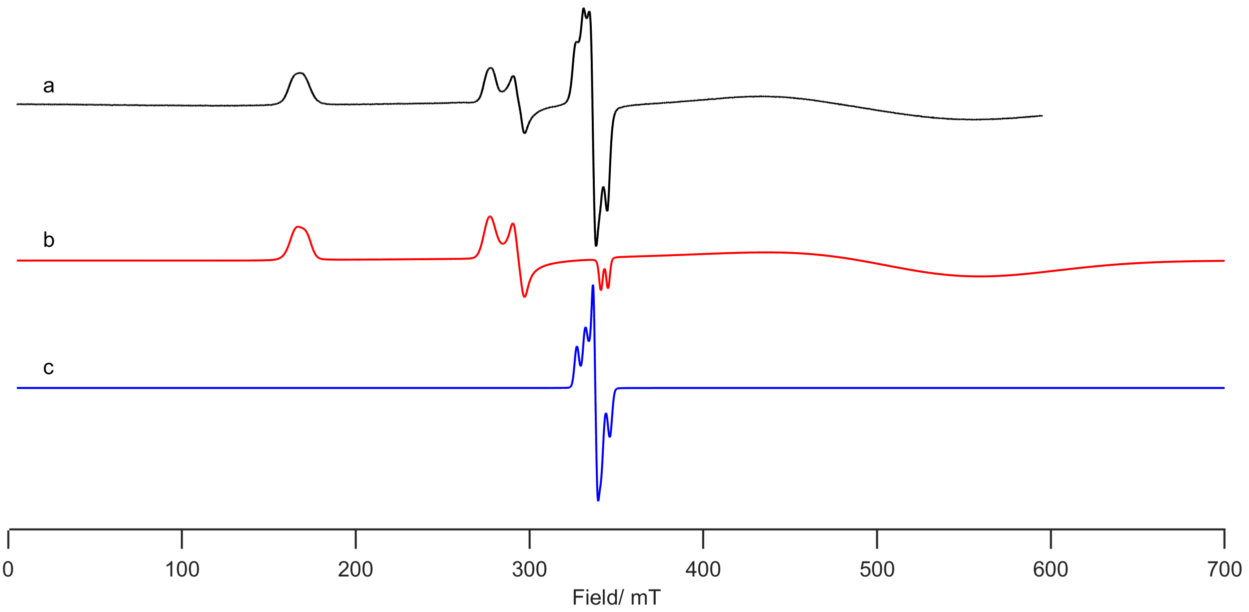

Figure S23. CW X-band EPR spectrum of **1** recorded in 4:1 v/v DFB/cyclohexane frozen glass at 115 K after being exposed to air. (a) experimental, (b) simulated mixture of **1** and **1**·DFB, and (c) simulated spectrum of superoxide derivative using spin Hamiltonian parameters: **g** = [2.051 2.000 1.966], **A**(^{103}Rh) = [137 28 137] MHz.

6.3. FB glass

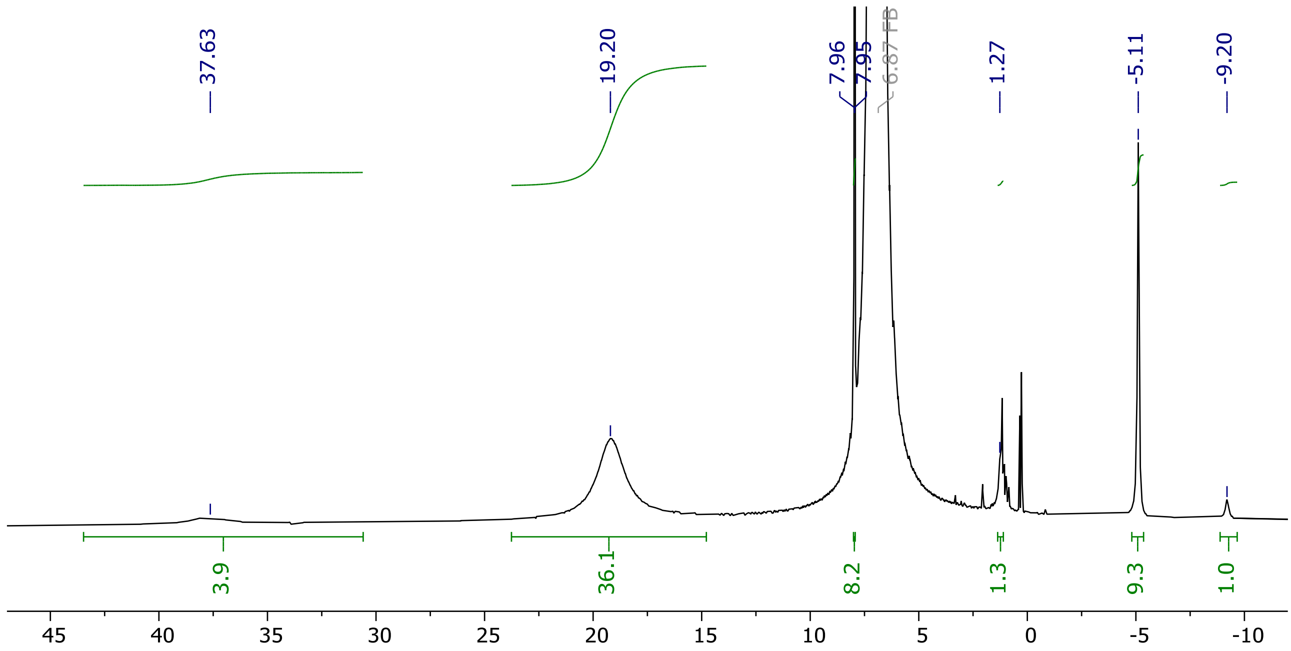

Figure S24. ^{1}H NMR spectrum of **1** (10 mM) recorded in FB after 24 h at RT (400 MHz).

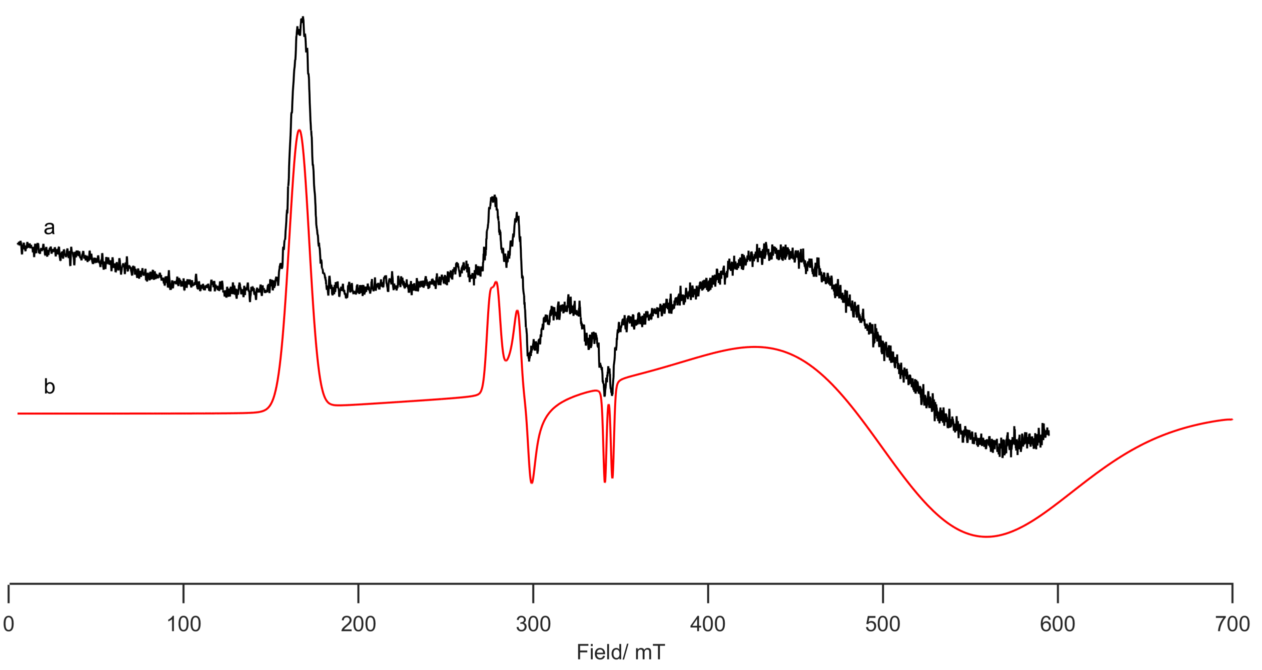

Figure S25. Experimental (a) and simulated (b) CW X-band EPR spectra of **1** recorded in 4:1 v/v FB/cyclohexane frozen glass at 115 K. **1**:**1**·FB ratio = 1:0.4.

6.4. TFT glass

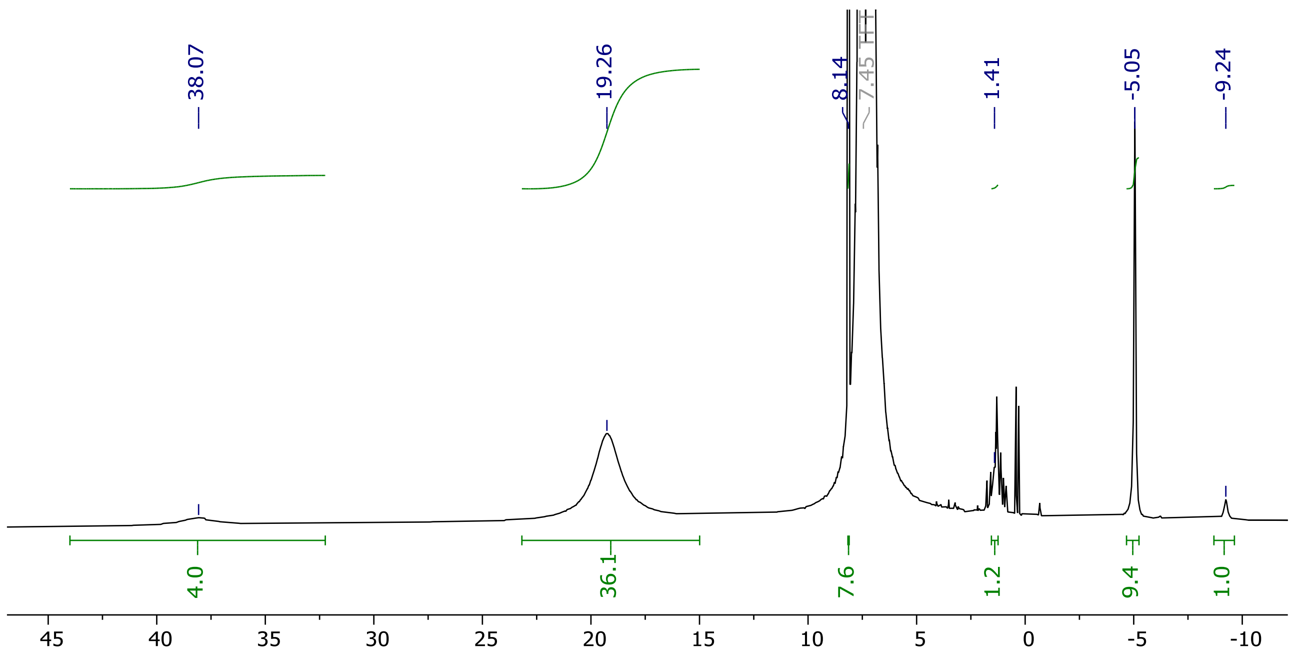

Figure S26. ^{1}H NMR spectrum of **1** (10 mM) recorded in TFT after 24 h at RT (400 MHz).

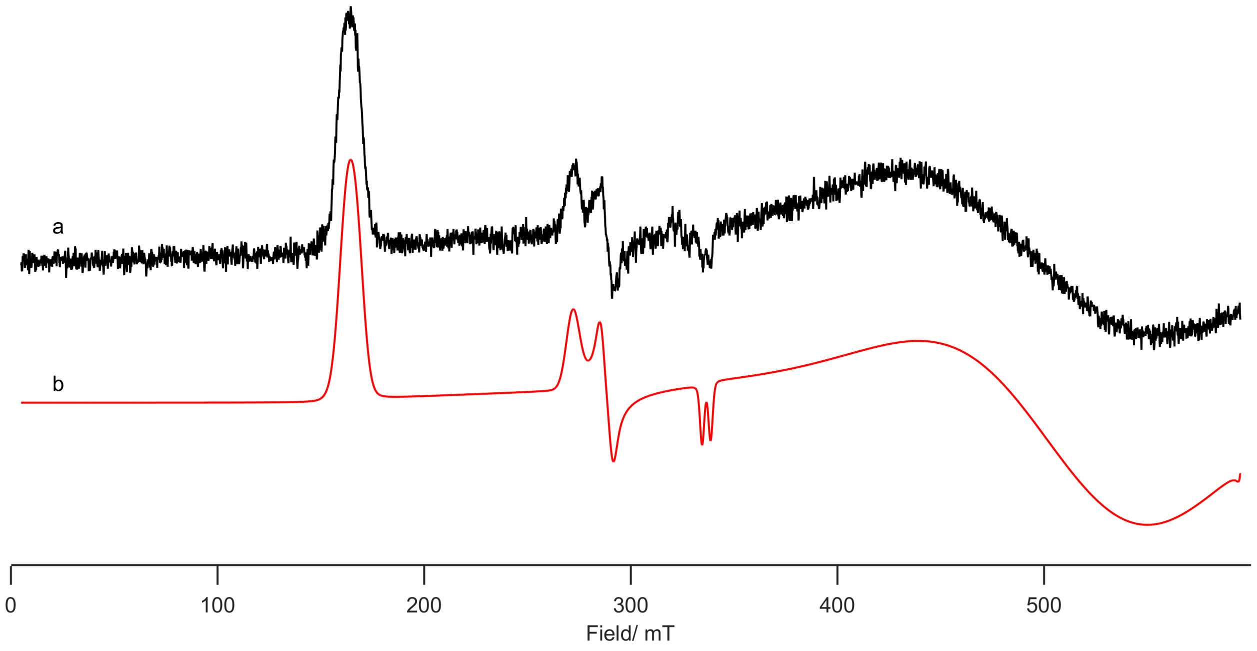

Figure S27. Experimental (a) and simulated (b) CW X-band EPR spectra of **1** recorded in 4:1 v/v TFT/cyclohexane frozen glass at 115 K. **1**:**1**·TFT ratio = 1:0.33.

6.5. THF glass

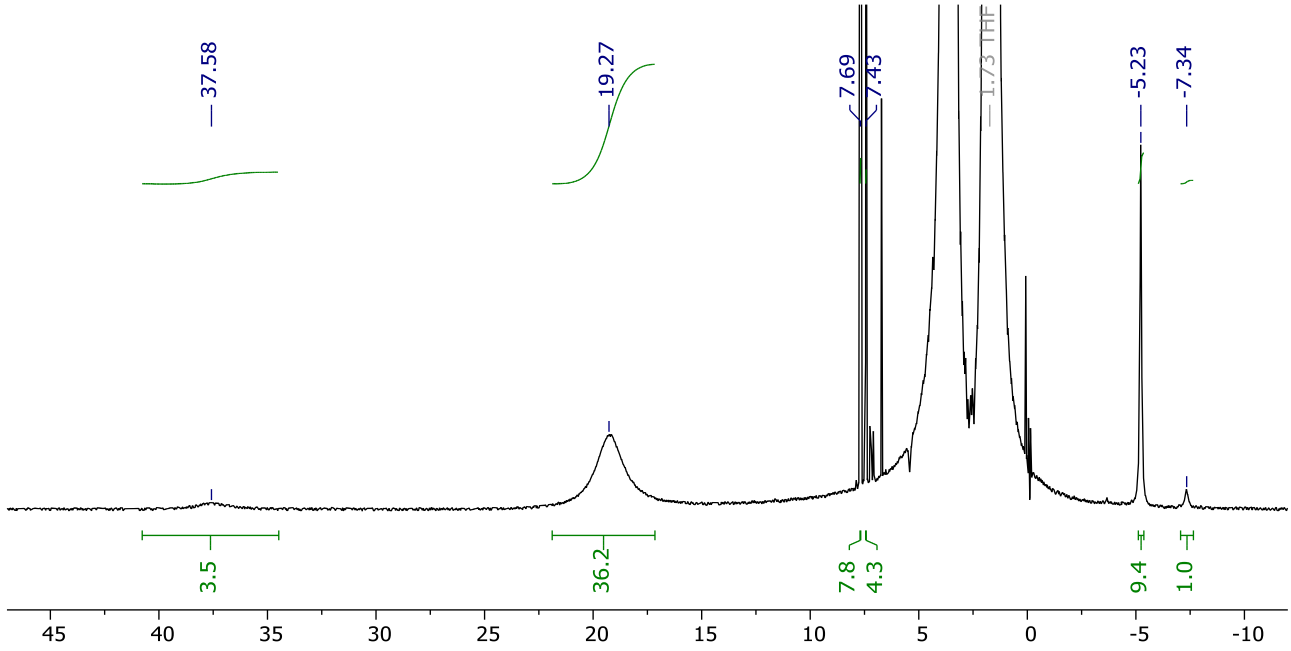

Figure S28. ^{1}H NMR spectrum of **1** (10 mM) recorded in THF after 24 h at RT (400 MHz).

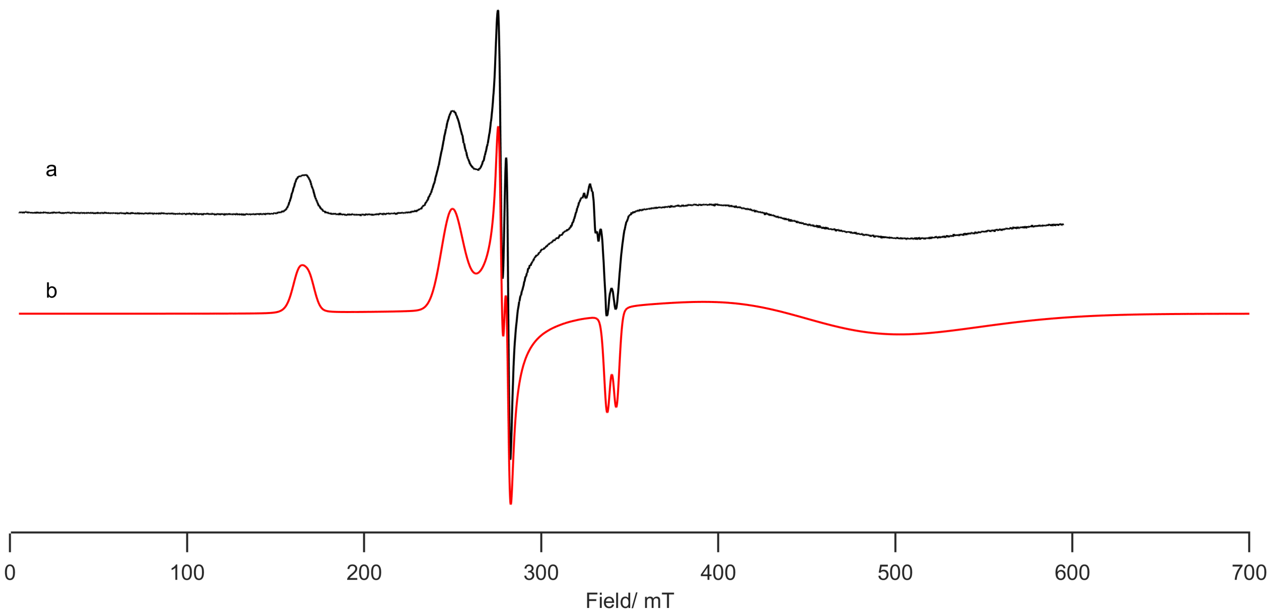

Figure S29. Experimental (a) and simulated (b) CW X-band EPR spectra of **1** recorded in 4:1 v/v THF/cyclohexane frozen glass at 115 K. **1**:**1**·THF ratio = 1:4 ratio and a trace quantity of the superoxide derivative can be observed at ~ 330 mT.

6.6. MeTHF glass

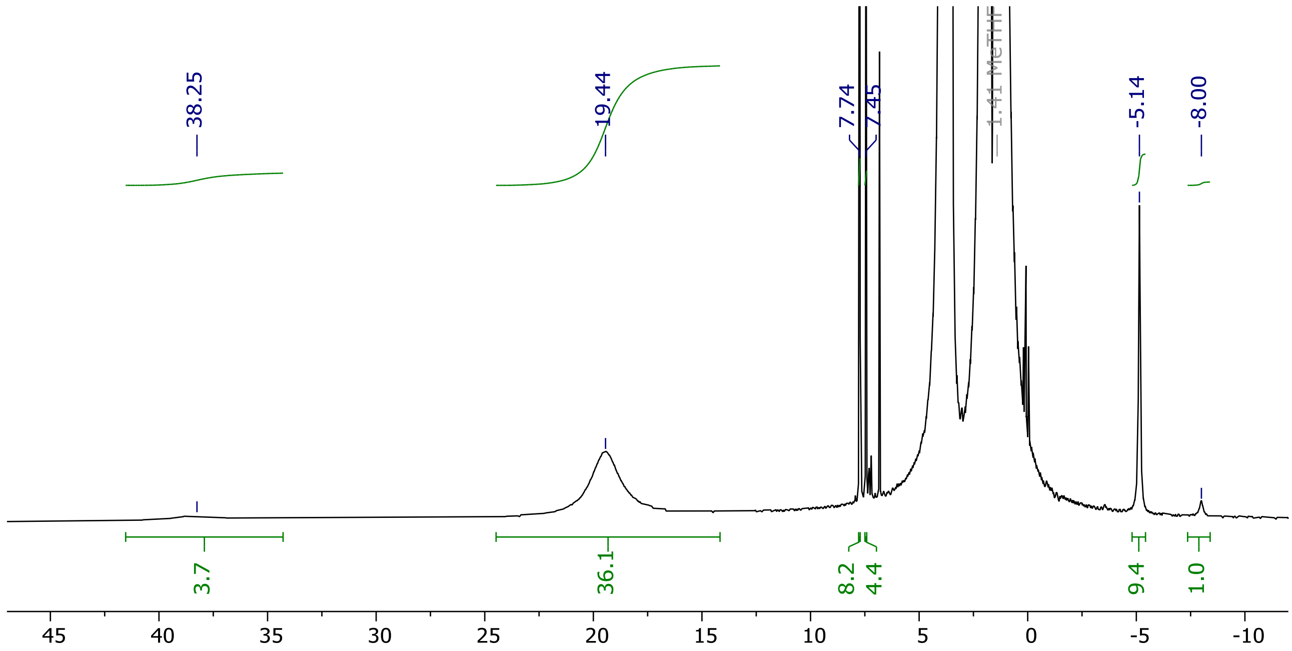

Figure S30. ^{1}H NMR spectrum of **1** (10 mM) recorded in MeTHF after 24 h at RT (400 MHz).

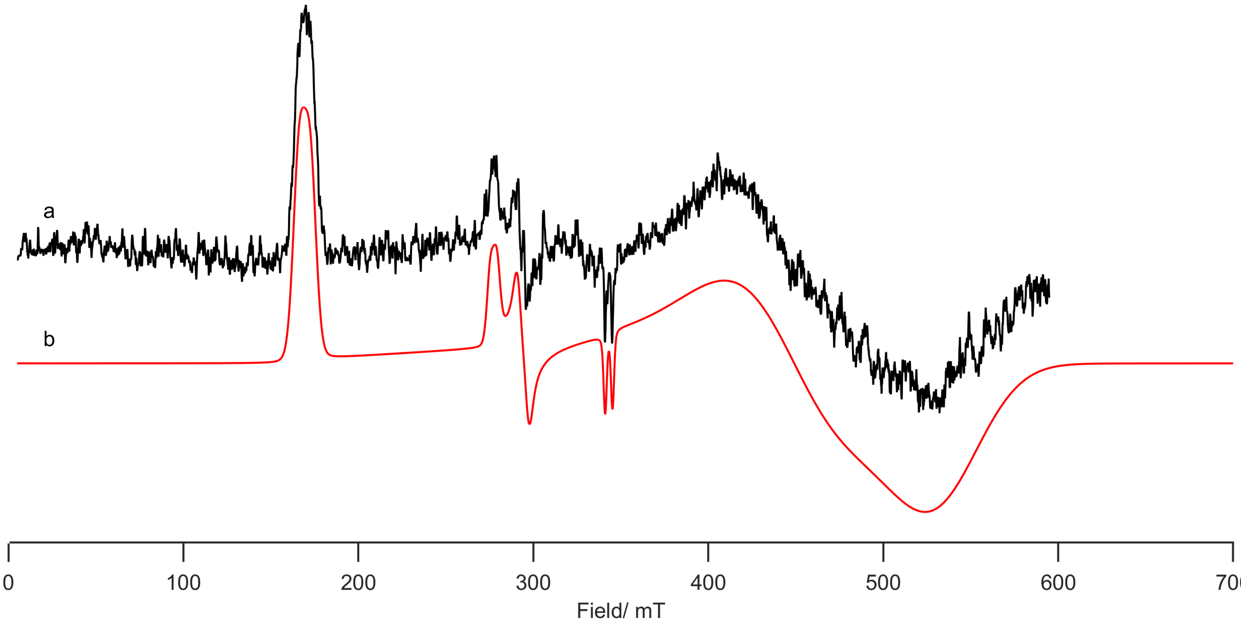

Figure S31. Experimental (a) and simulated (b) CW X-band EPR spectra of 1 recorded in 4:1 v/v MeTHF/cyclohexane frozen glass at 115 K. **1**:**1**·MeTHF ratio = 1:0.4.

7. Reaction of [Rh(PNP-*t*Bu)(C≡C*t*Bu)][BAr$^F_4$] 1 with 9,10-dihydroanthracene

7.1. Reaction in DFB

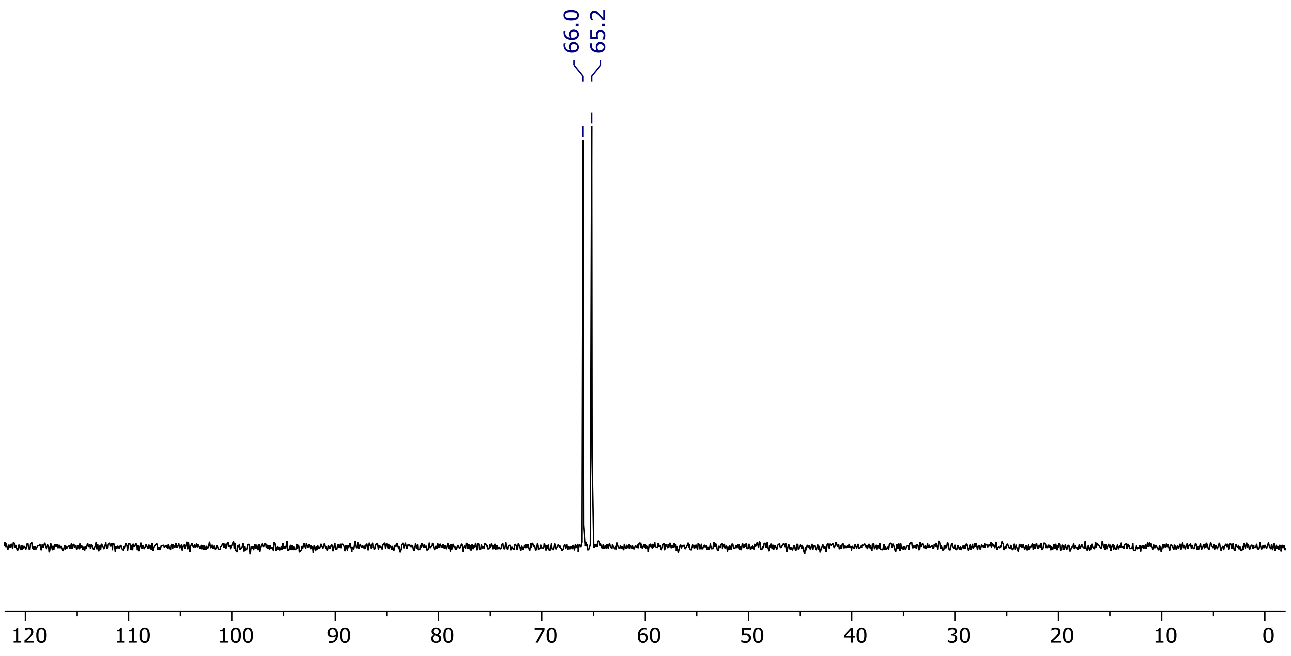

Figure S32. ^{31}P{^{1}H} NMR spectrum of **1** + 9,10-dihydroanthracene in DFB recorded after 14 days at 60 °C (162 MHz).

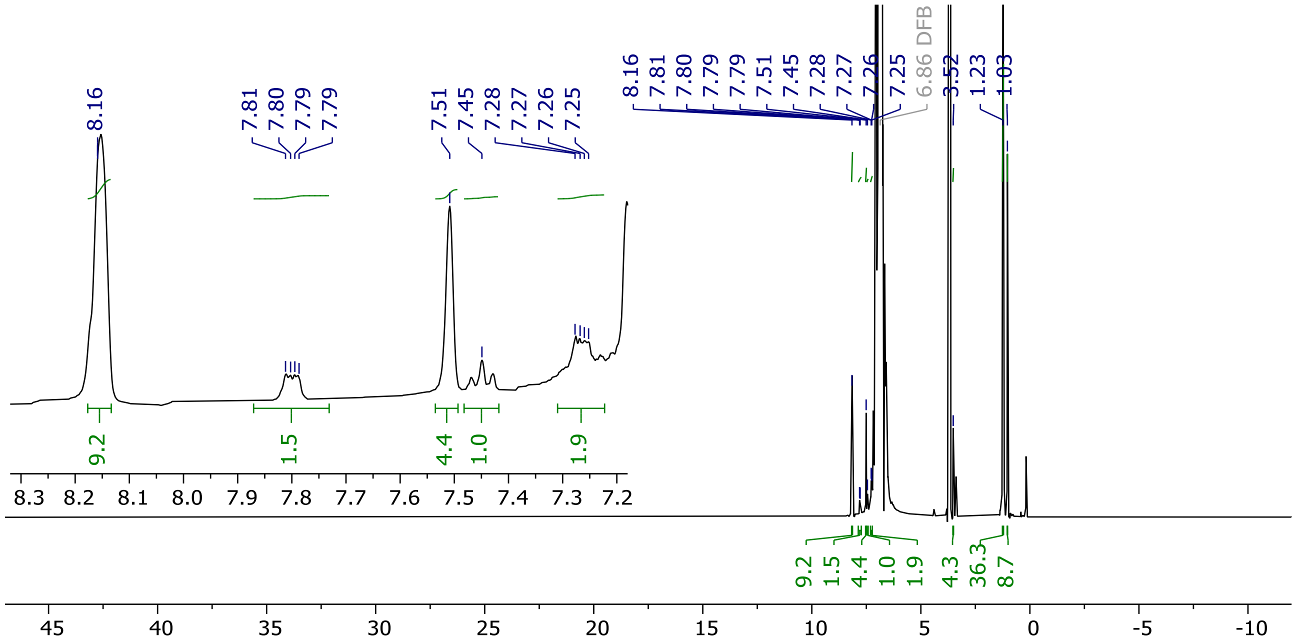

Figure S33. ^{1}H NMR spectrum of **1** + 9,10-dihydroanthracene in DFB recorded after 14 days at 60 °C (400 MHz).

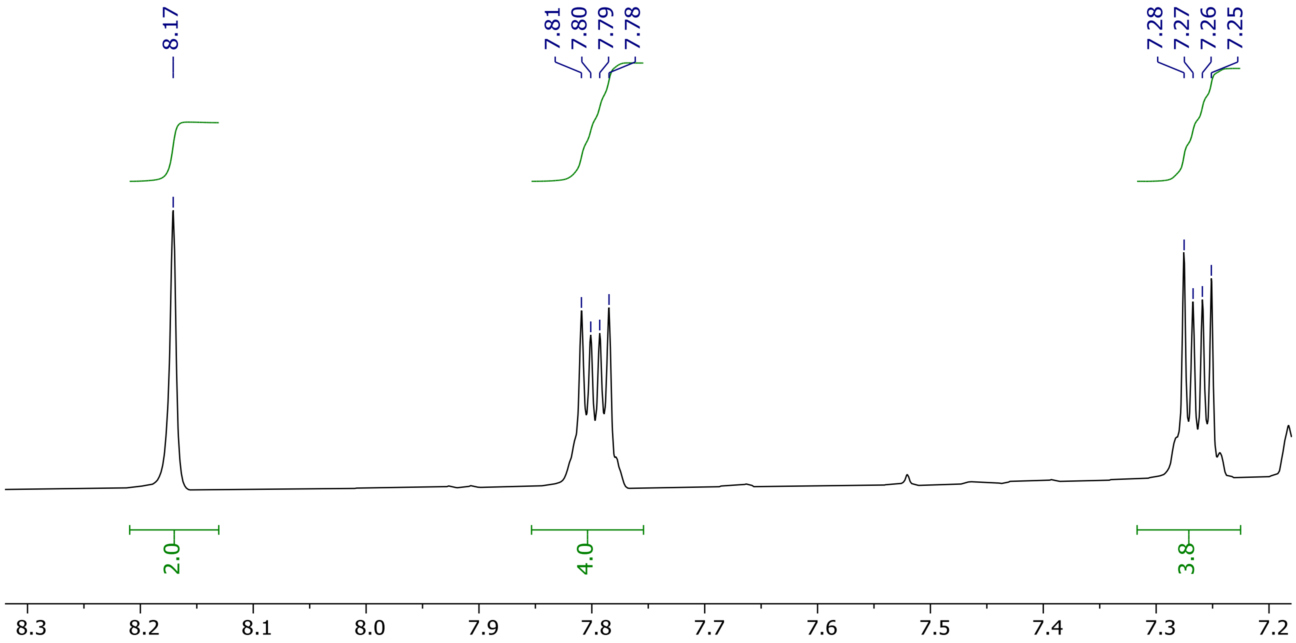

Figure S34. ^{1}H NMR spectrum of anthracene (400 MHz, DFB).

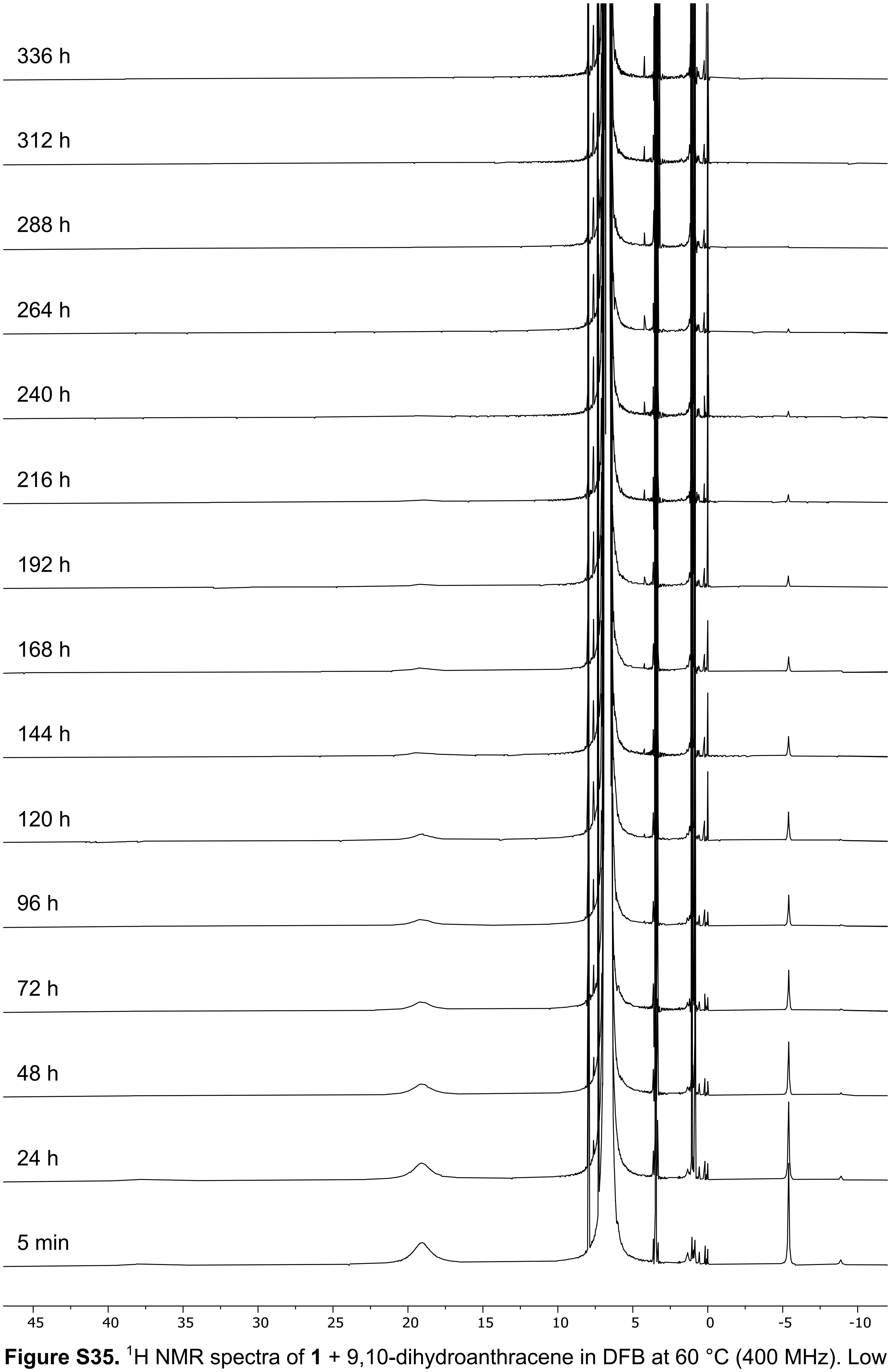

Figure S35. ^{1}H NMR spectra of **1** + 9,10-dihydroanthracene in DFB at 60 °C (400 MHz). Low intensity peak at δ 4.40 is attributed to the presence of a small quantity of H_2.

7.2. Reaction in FB

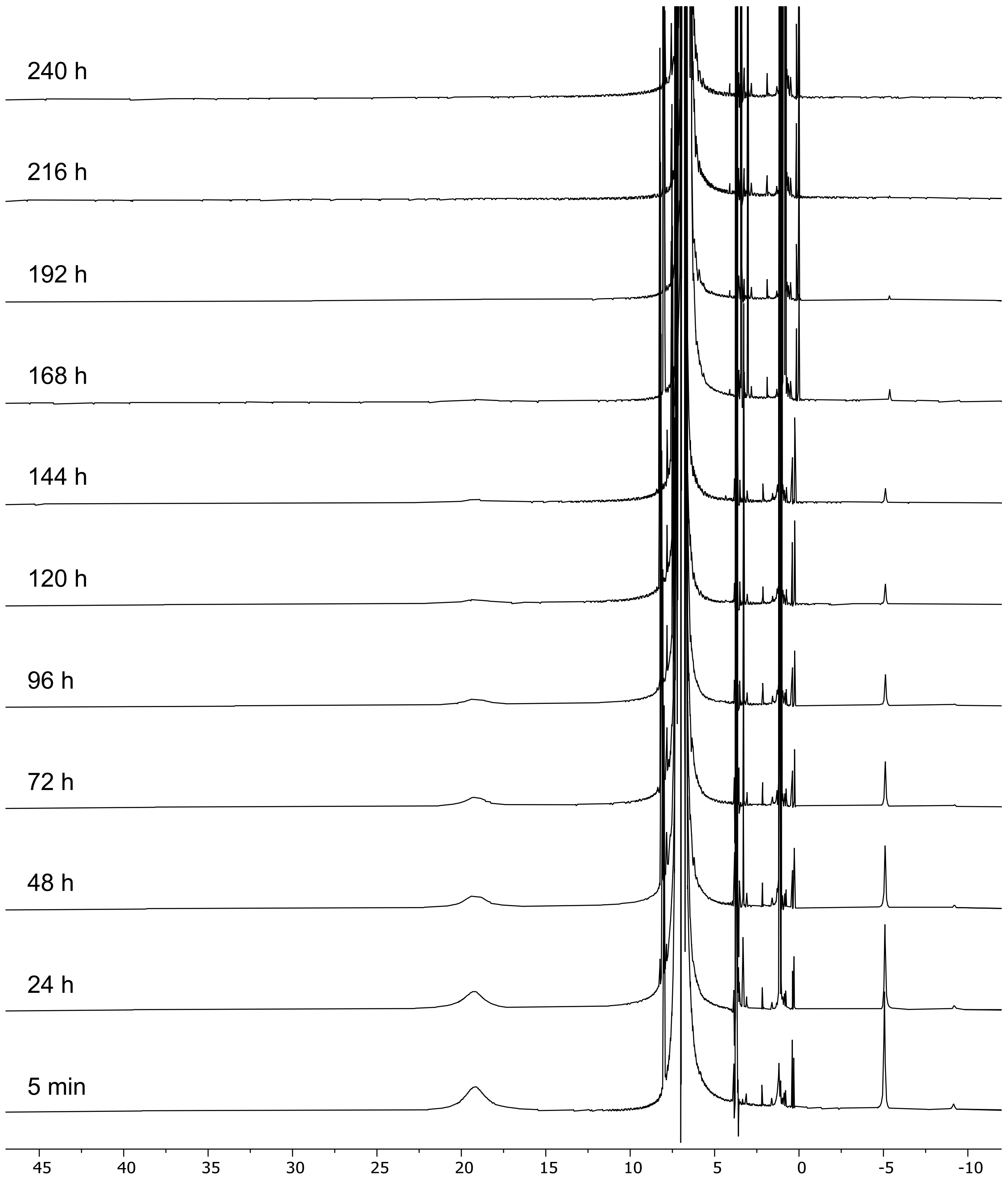

Figure S36. ^{1}H NMR spectra of **1** + 9,10-dihydroanthracene in FB at 60 °C (400 MHz). Very low intensity peak at δ 4.37 is attributed to the presence of a trace quantity of H_2.

7.3. Reaction in TFT

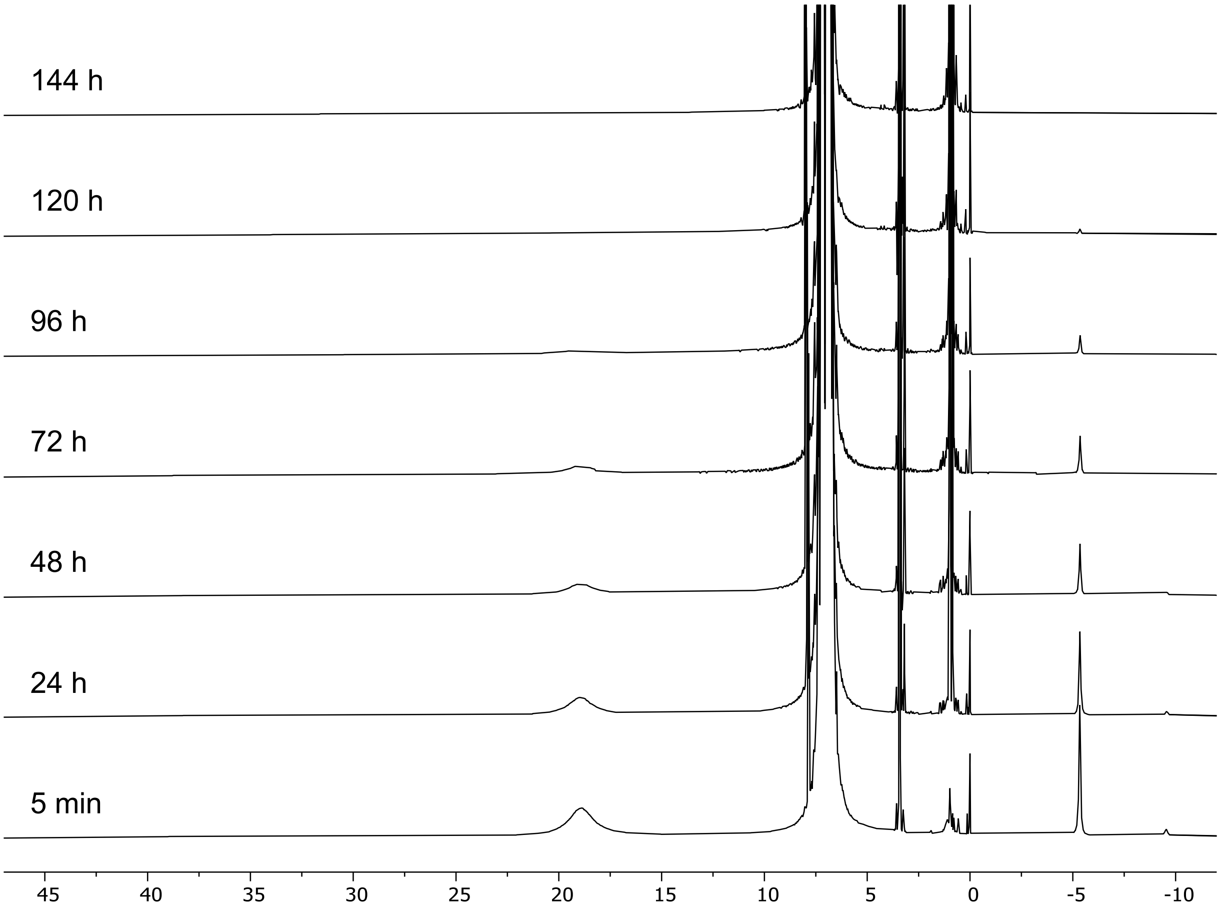

Figure S37. ^1H NMR spectra of **1** + 9,10-dihydroanthracene in TFT at 60 °C (400 MHz).

7.4. Reaction in THF

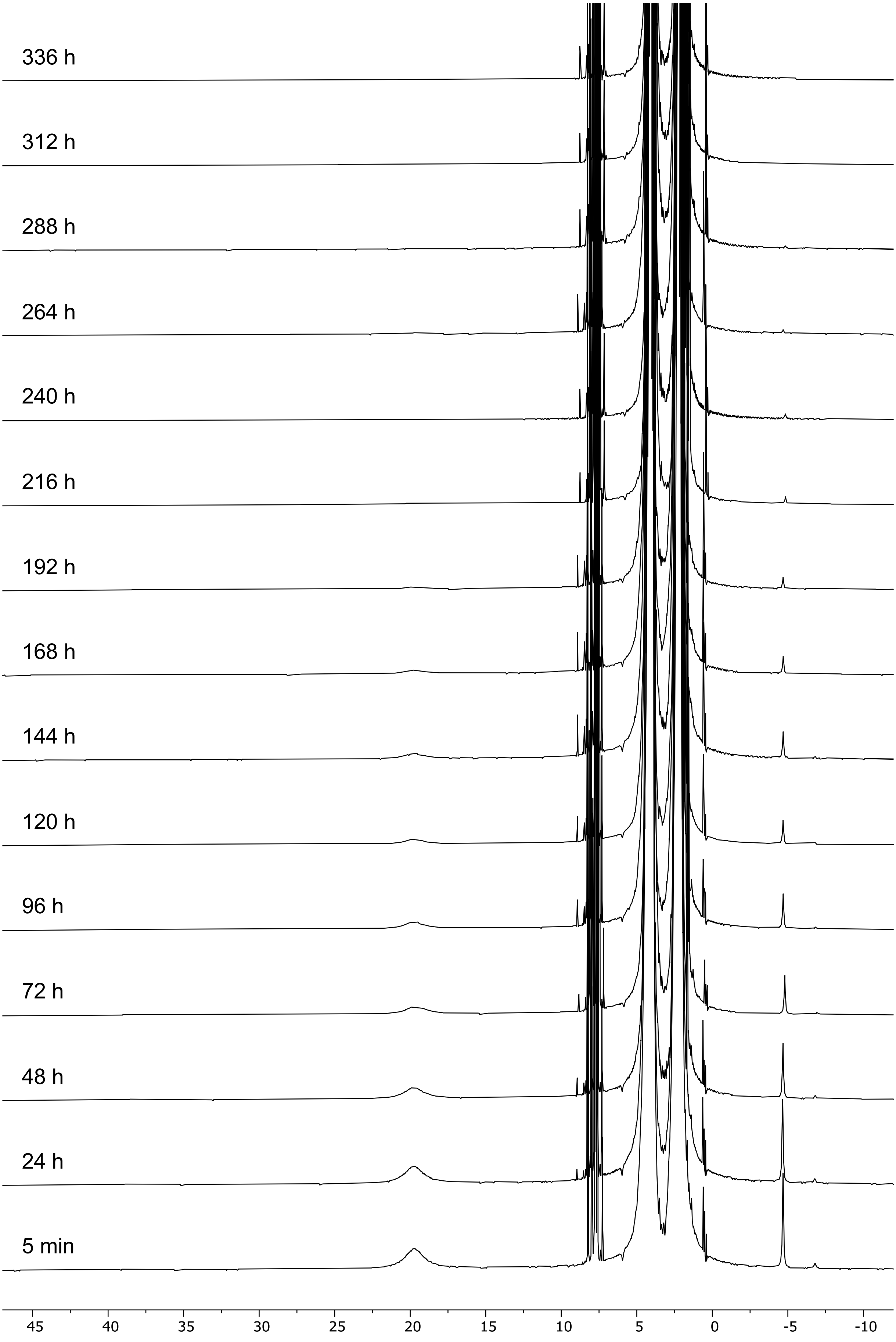

Figure S38. ^{1}H NMR spectra of **1** + 9,10-dihydroanthracene in THF at 60 °C (400 MHz).

7.5. Reaction in MeTHF

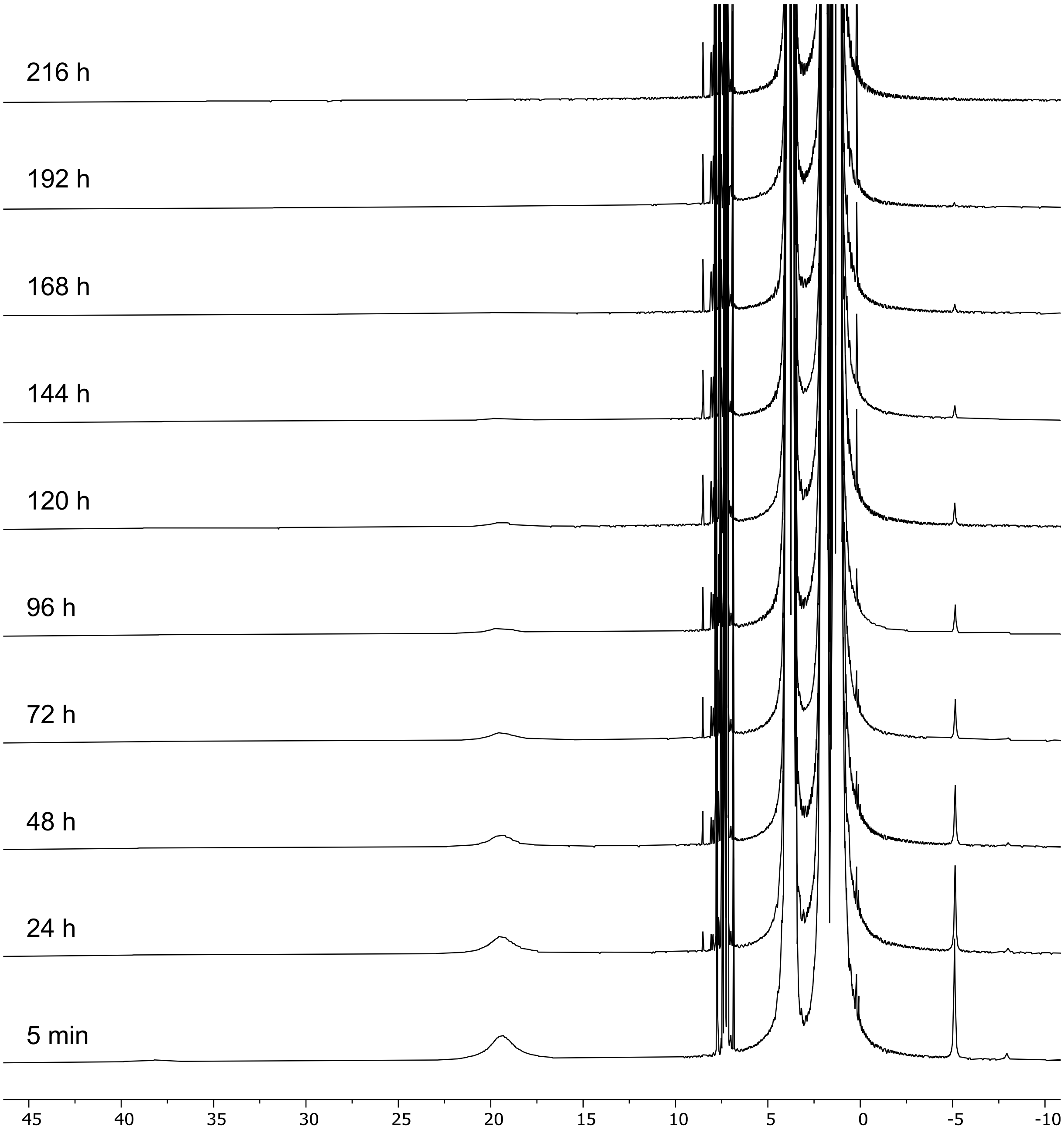

Figure S39. ^{1}H NMR spectra of **1** + 9,10-dihydroanthracene in MeTHF at 60 °C (400 MHz).

7.6. Isolation of [Rh(PNP-*t*Bu)(C=CH*t*Bu)][BAr$^F_4$] 2

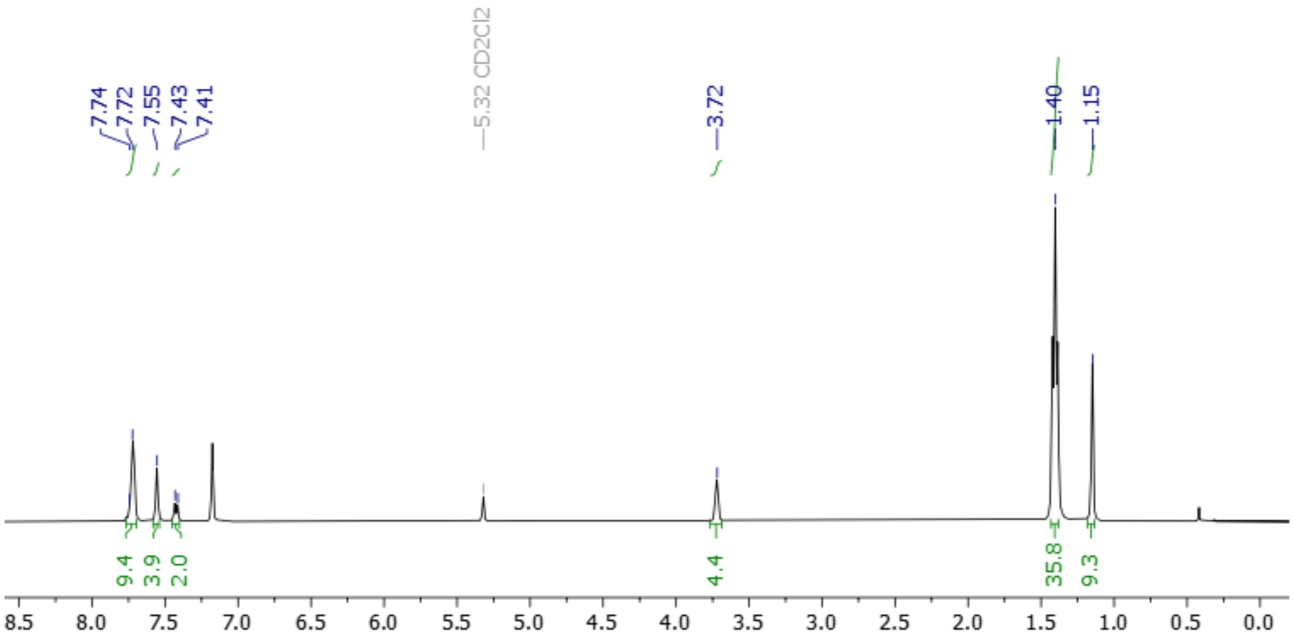

Figure S40. ^1H NMR spectrum of **2** (400 MHz, CD_2Cl_2).

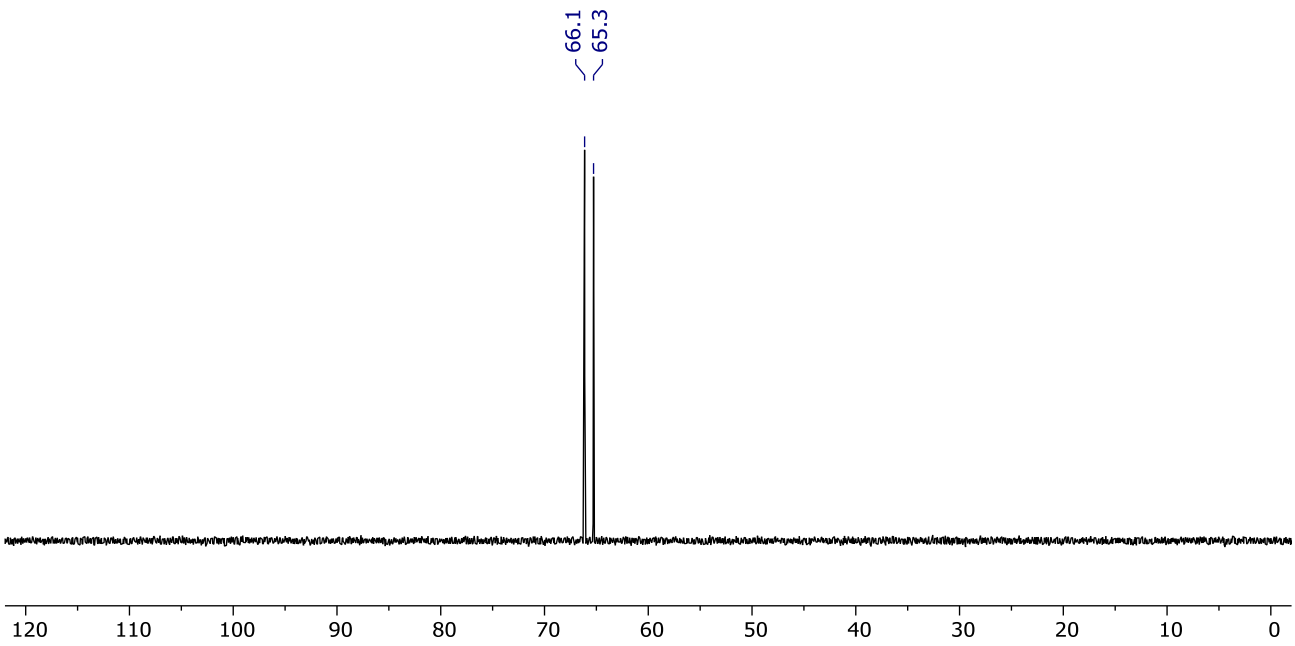

Figure S41. $^{31}P\{^1H\}$ NMR spectrum of **2** (162 MHz, CD_2Cl_2).

8. Reaction of [Rh(PNP-*t*Bu)(C=CH*t*Bu)][BAr$^F_4$] 2 with CD_3CN

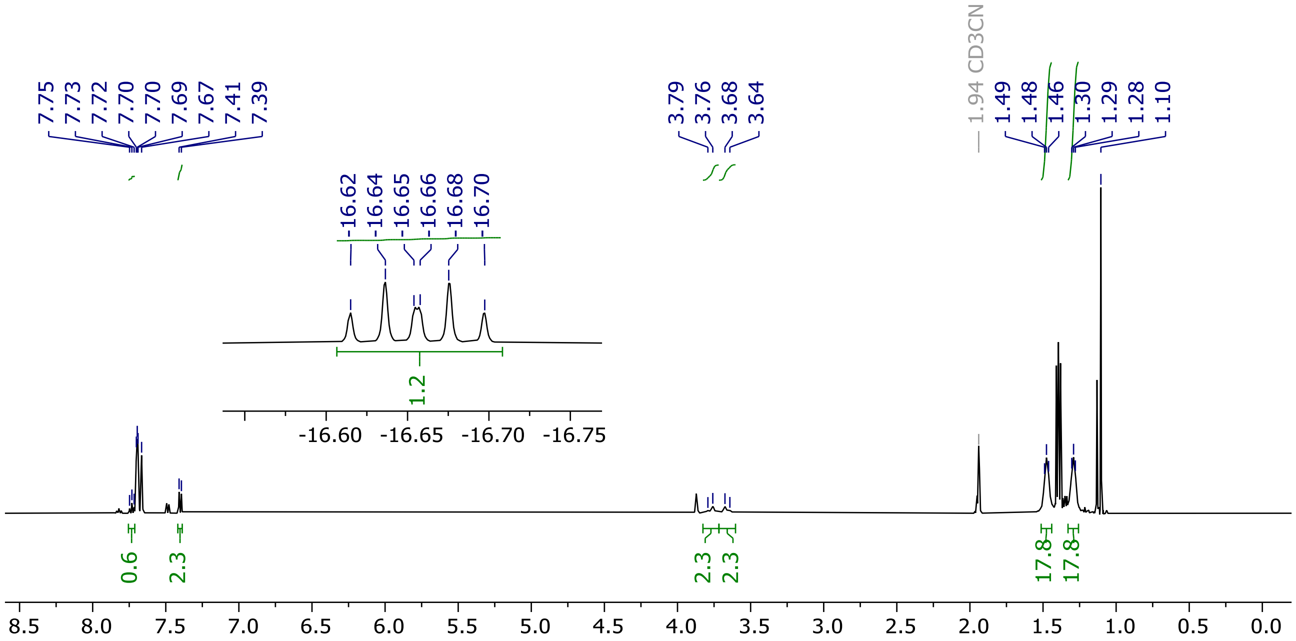

Figure S42. ^{1}H NMR spectrum of an equilibrium mixture of **2** and **3**·ACN (500 MHz, CD_3CN).

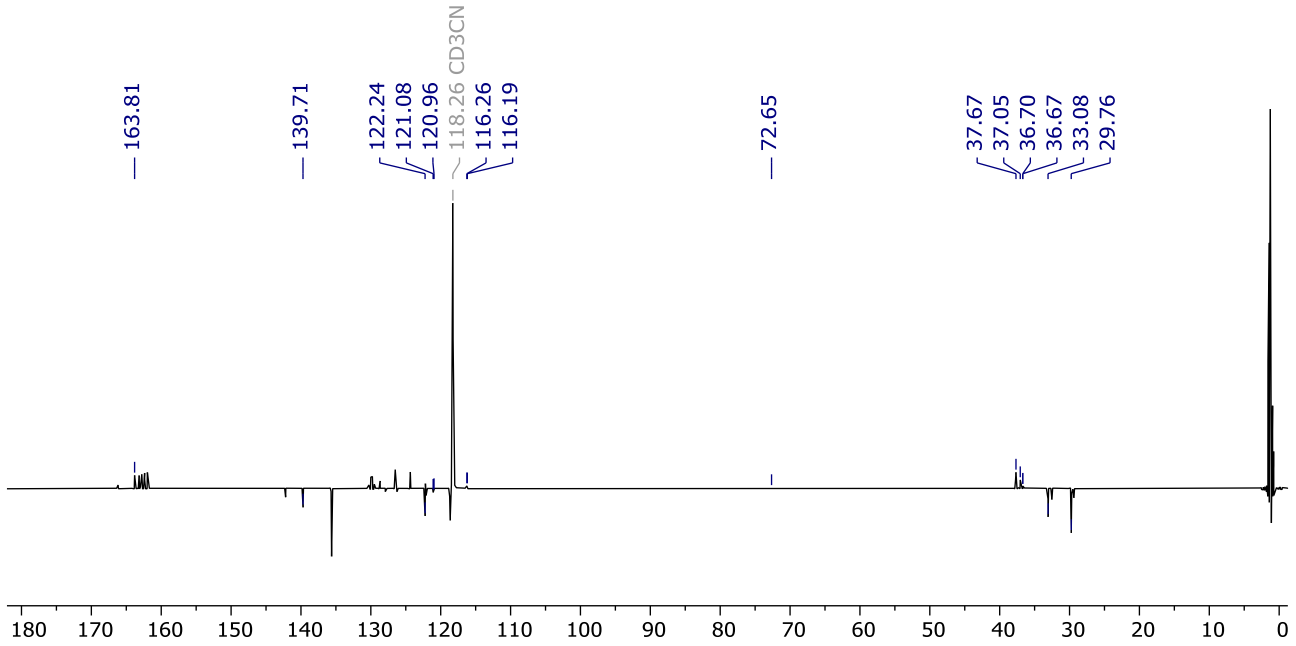

Figure S43. $^{13}C\{^1H\}$ APT NMR spectrum of an equilibrium mixture of **2** and **3**·ACN (126 MHz, CD_3CN).

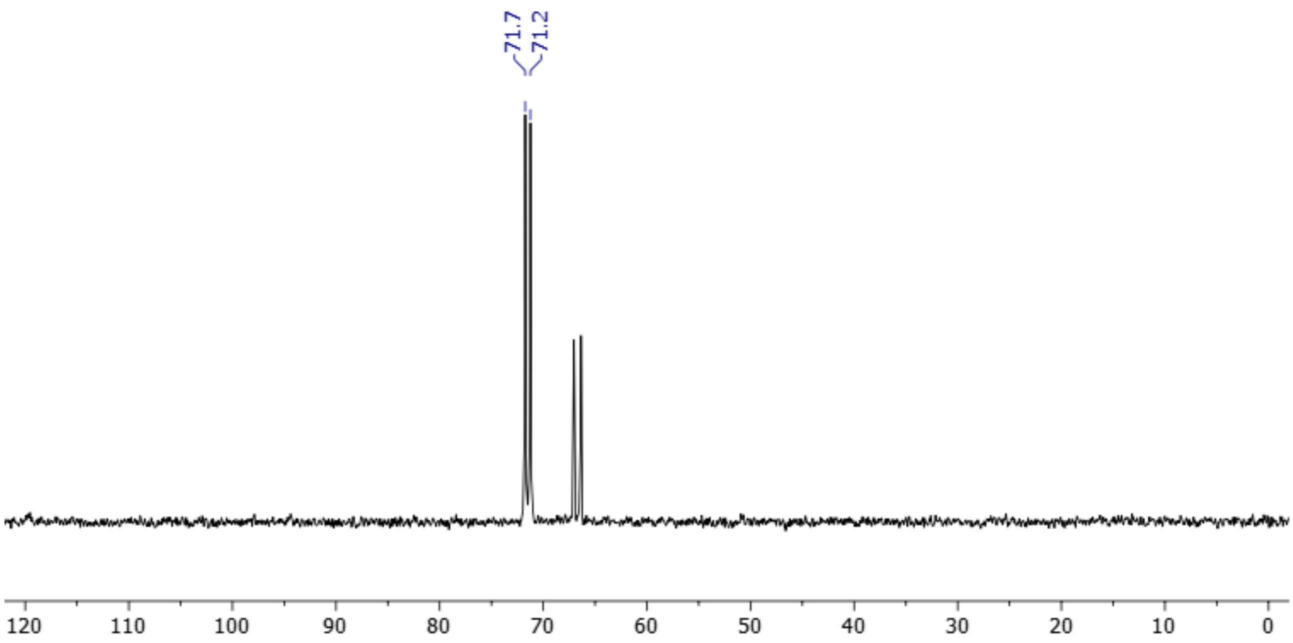

Figure S44. $^{31}P\{^1H\}$ NMR spectrum of an equilibrium mixture of **2** and **3**·ACN (202 MHz, CD_3CN).

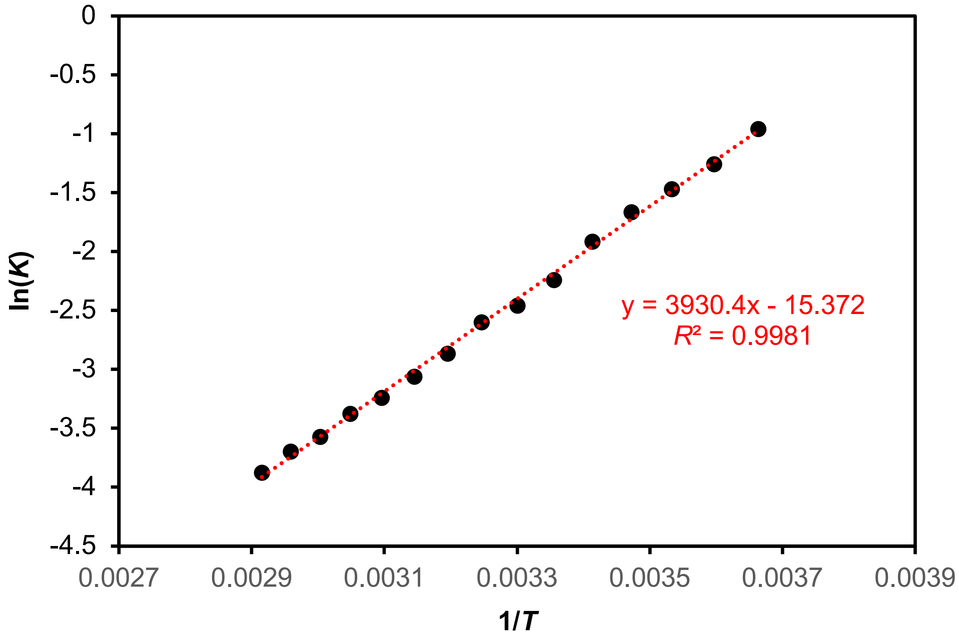

Figure S45. Van't Hoff plot for the variable temperature ^1H NMR data collected for the equilibrium mixture of **2** and **3**·ACN.

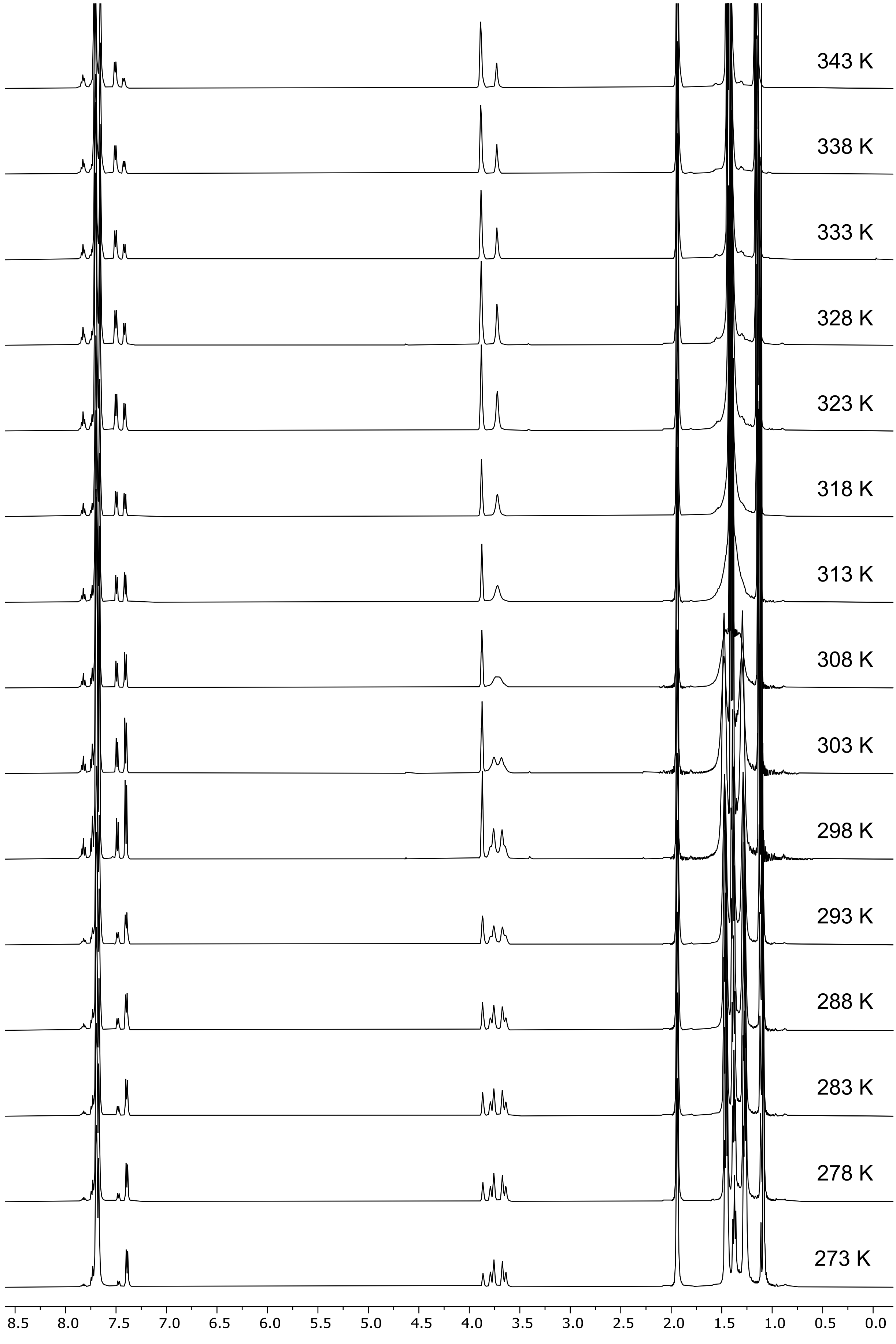

Figure S46. Variable temperature ^1H NMR spectra of an equilibrium mixture of **2** and **3**·ACN (500 MHz, CD_3CN).

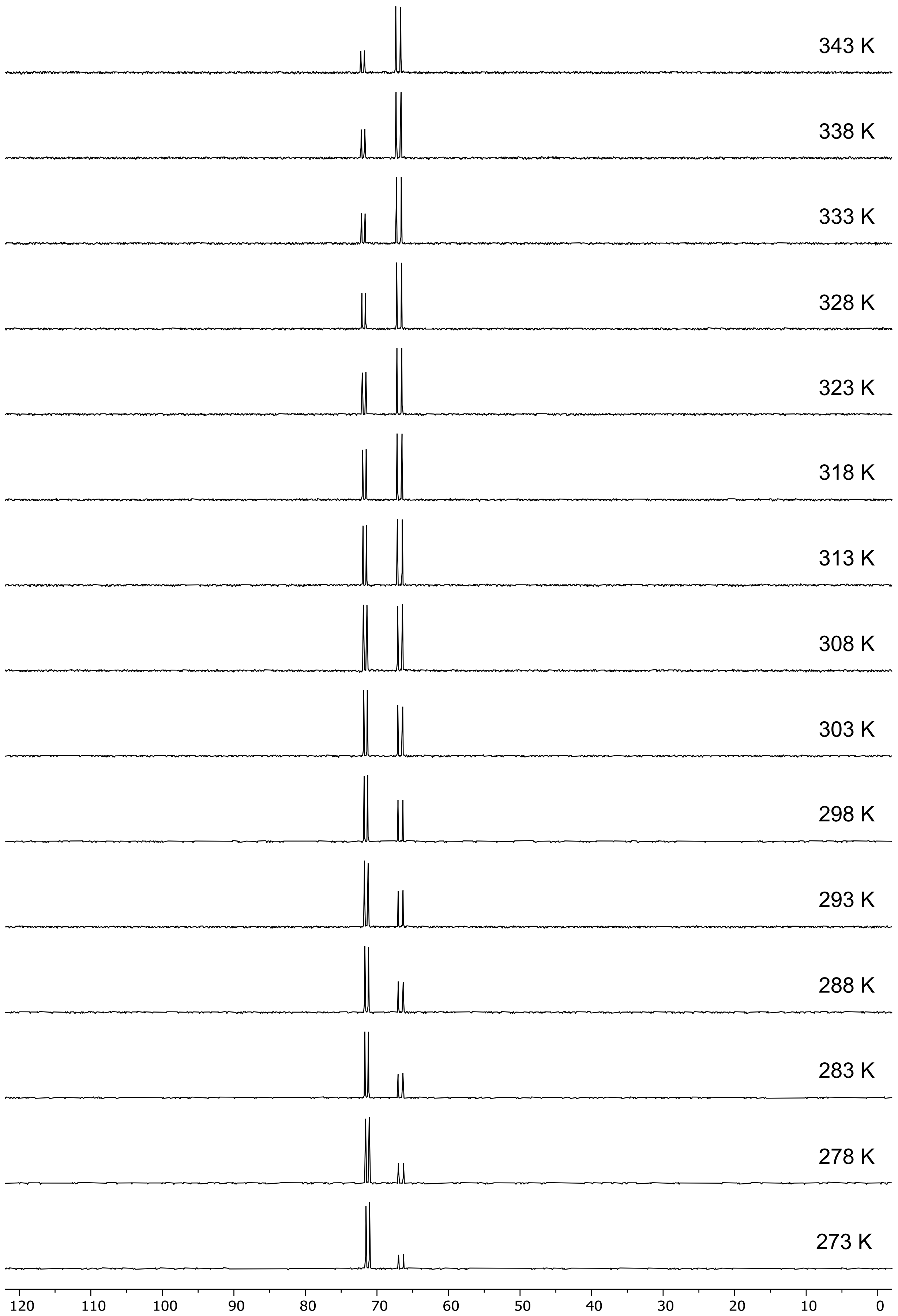

Figure S47. Variable temperature $^{31}P\{^{1}H\}$ NMR spectra of an equilibrium mixture of **2** and **3**·ACN (202 MHz, CD_3CN).

9. Computational details

9.1. Molecular orbital diagram of 1

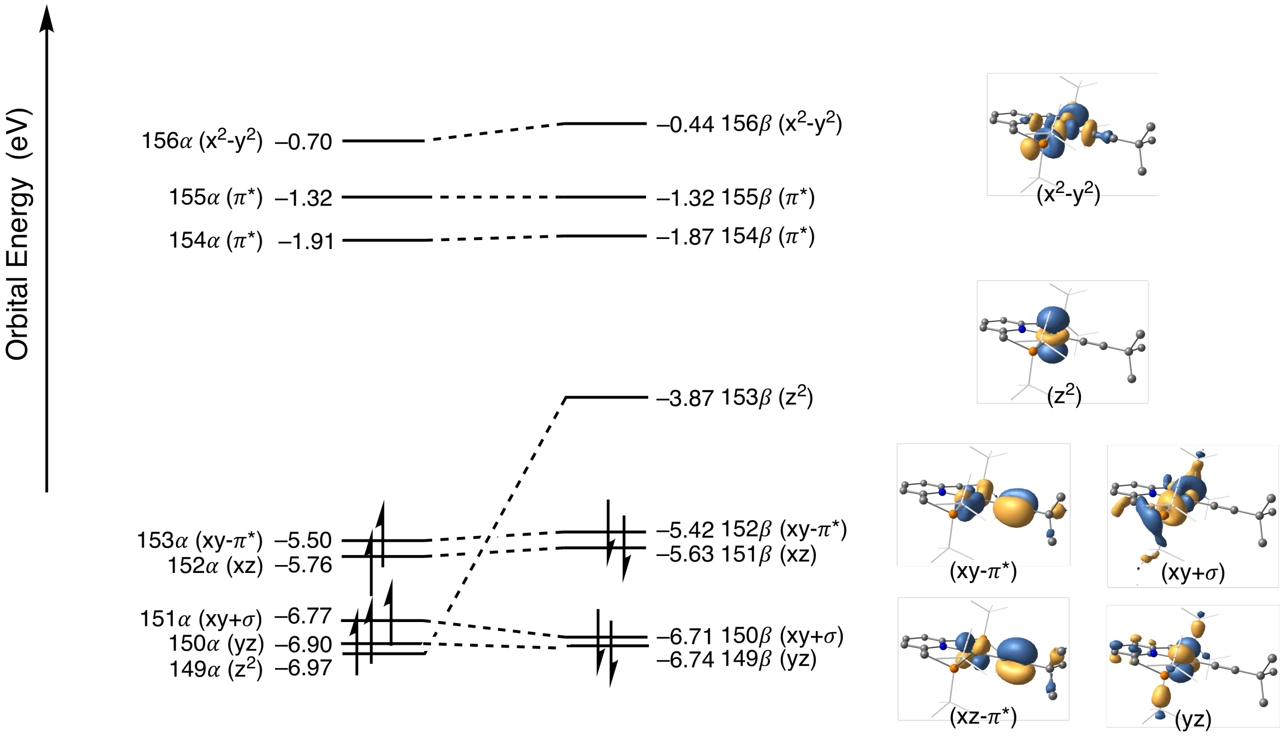

Figure S48. Molecular orbital diagram of **1** (d_{z2} ground state) at the M06-D3/SDD/6-311++G(2d,2p)//M06L-D3/SDD/6-31G(d,p) level of theory.

9.2. Reaction profiles

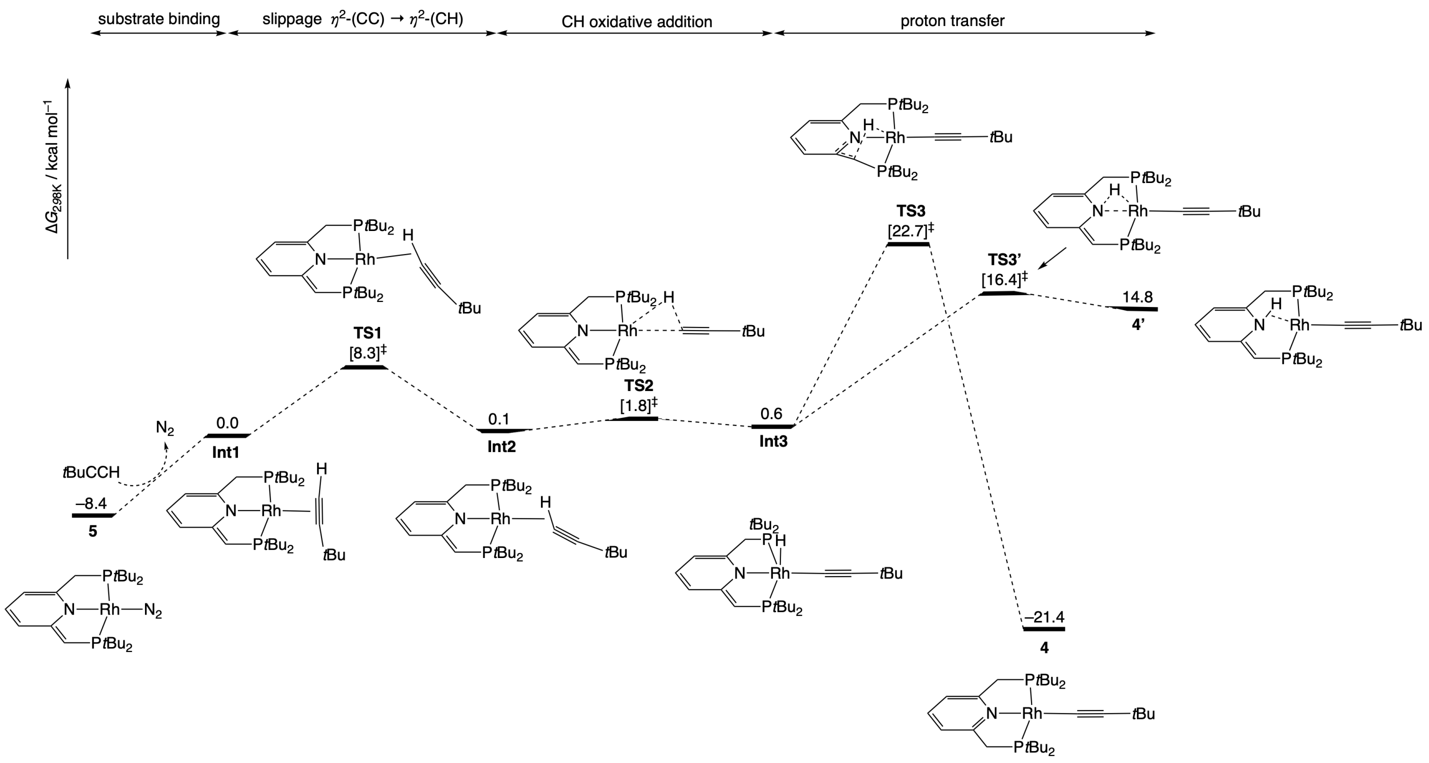

Figure S49. Computed Free Energy Profile (M06-D3/SDD/6-311++G(2d,2p)//M06L-D3/SDD/6-31G(d,p) corrected for cyclohexane solvent) for the formation of rhodium(I) alkynyl complex **4**. Relative Gibbs Energies (298 K, 1 atm) are given in kcal·mol^{-1}.

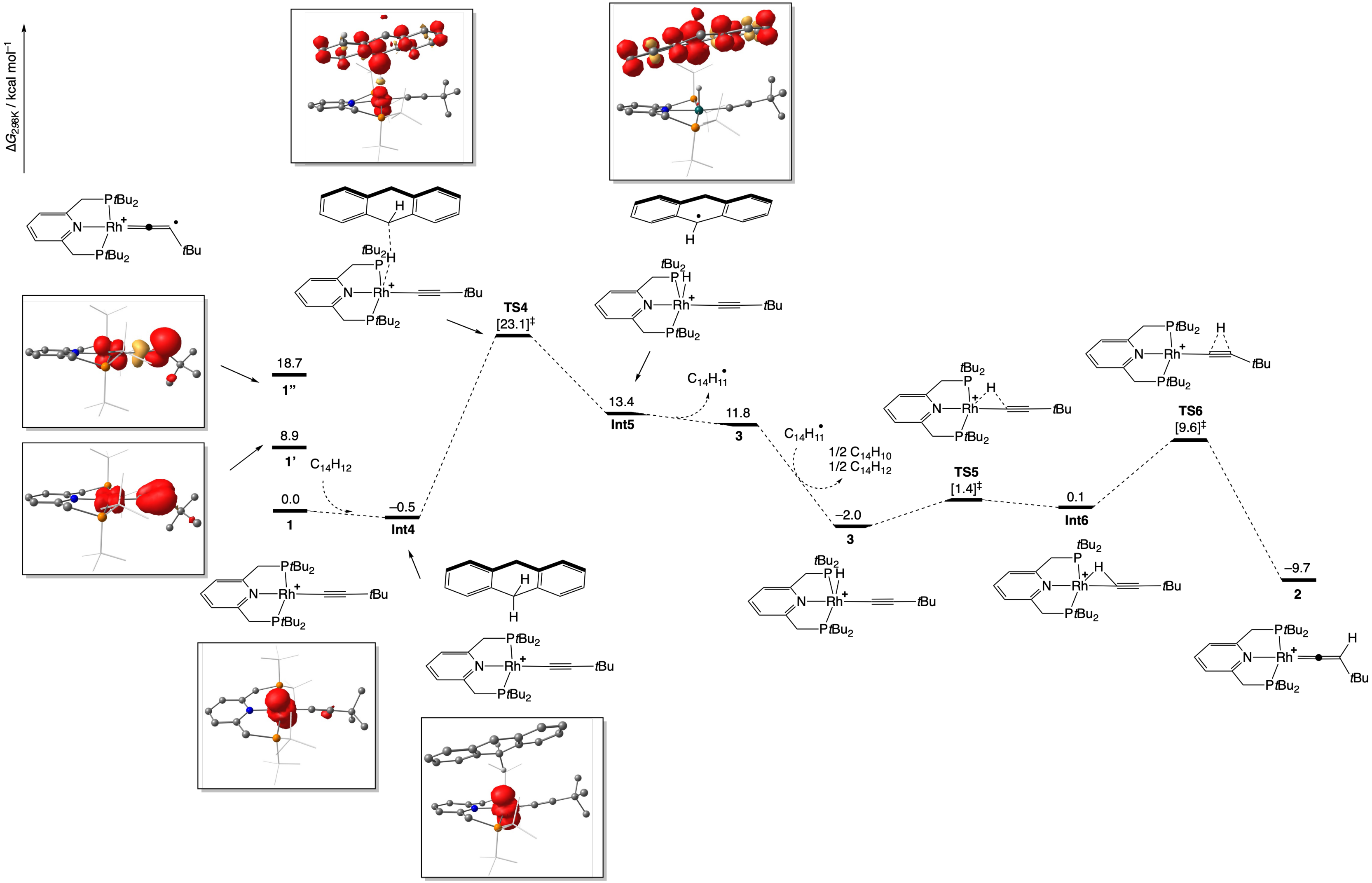

Figure S50. Computed Free Energy Profile (M06-D3/SDD/6-311++G(2d,2p)//M06L-D3/SDD/6-31G(d,p) corrected for DFB solvent) for the formation of **2** from **1** by carbon-to-metal hydrogen atom transfer from 9,10-dihydroanthracene. Depicted with 1,3-hydride migration occurring <u>after</u> termination of the hydroanthracenyl radical into anthracene. Insets show α-spin density plots. Relative Gibbs Energies (298 K, 1 atm) are given in kcal·mol^{-1}.

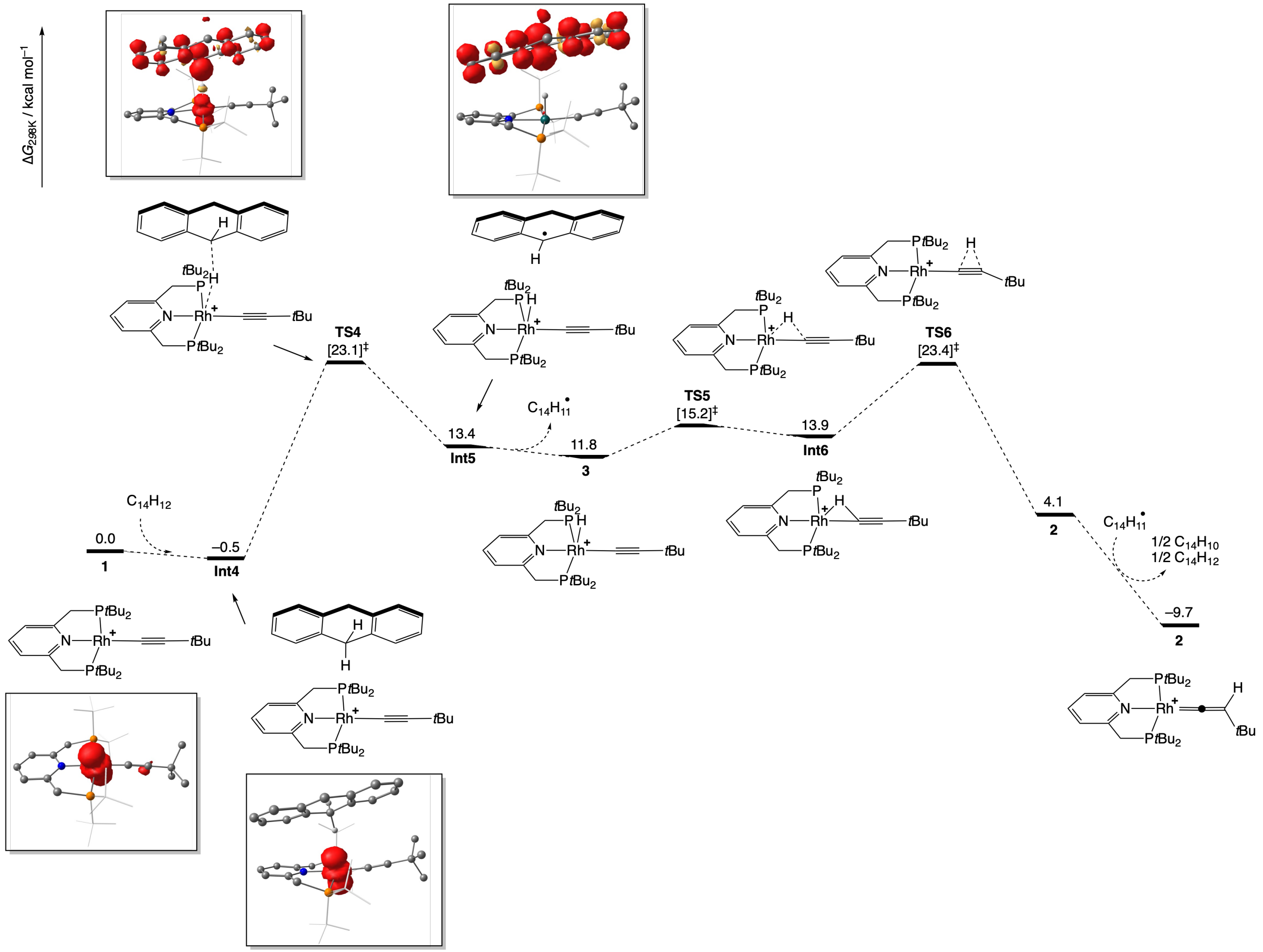

Figure S51. Computed Free Energy Profile (M06-D3/SDD/6-311++G(2d,2p)//M06L-D3/SDD/6-31G(d,p) corrected for DFB solvent) for the formation of **2** from **1** by carbon-to-metal hydrogen atom transfer from 9,10-dihydroanthracene. Depicted with 1,3-hydride migration occurring before termination of the hydroanthracenyl radical into anthracene. Insets show α-spin density plots. Relative Gibbs Energies (298 K, 1 atm) are given in kcal·mol^{-1}.

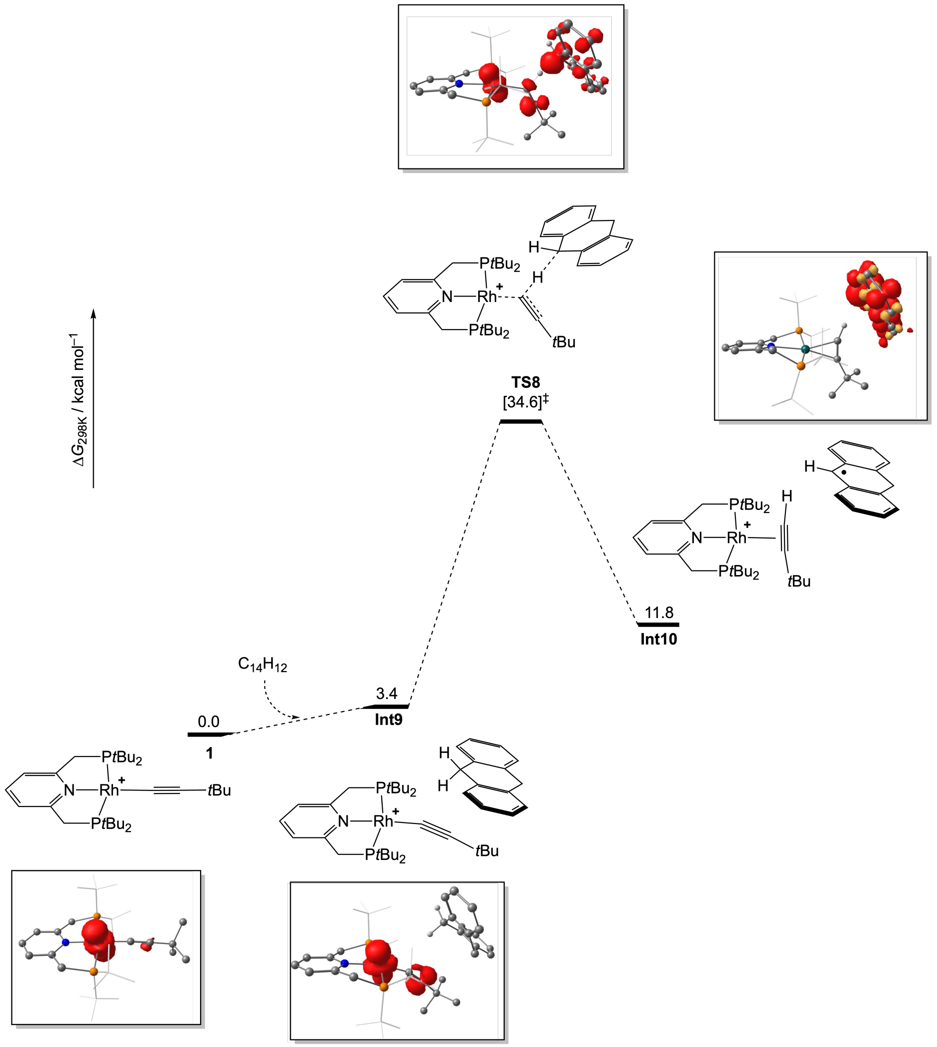

Figure S52. Computed Free Energy Profile (M06-D3/SDD/6-311++G(2d,2p)//M06L-D3/SDD/6-31G(d,p) corrected for DFB solvent) for the formation of **2** from **1** by carbon-to-β-carbon hydrogen atom transfer from 9,10-dihydroanthracene. Insets show α-spin density plots. Relative Gibbs Energies (298 K, 1 atm) are given in kcal·mol^{-1}.

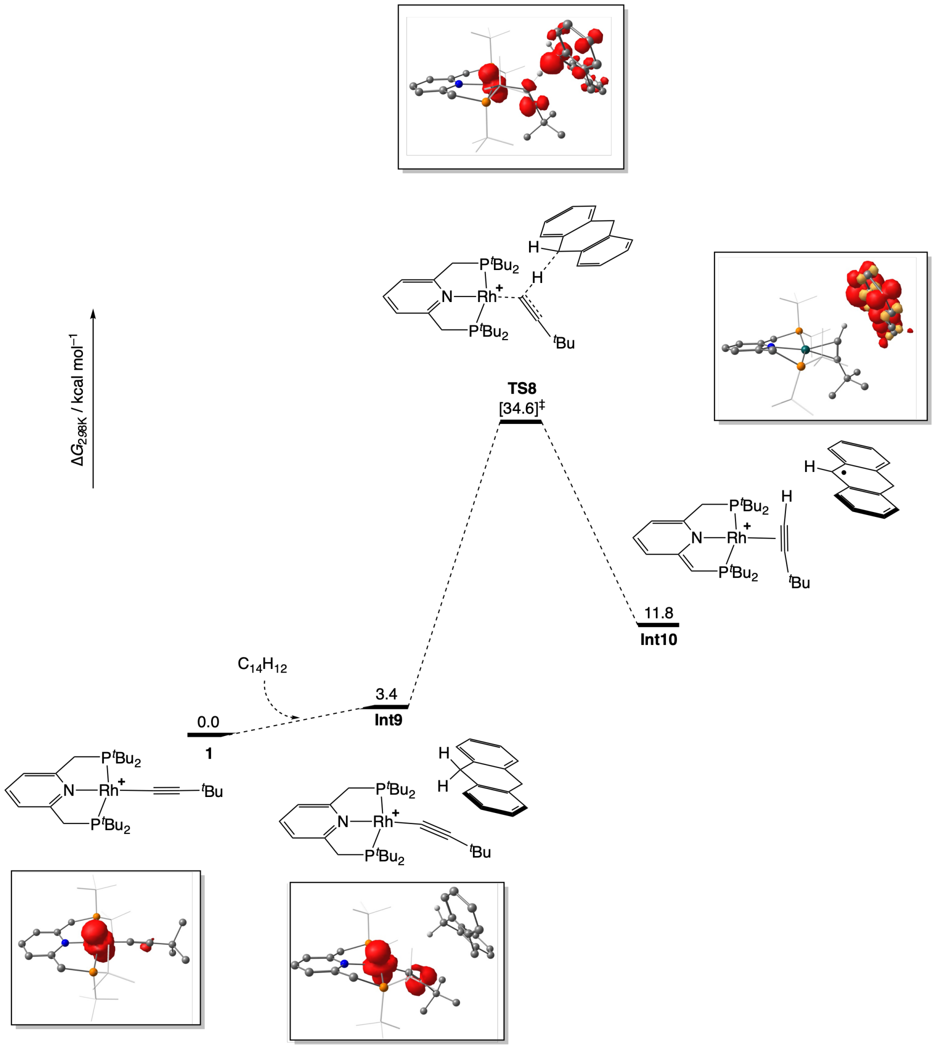

Figure S53. Computed Free Energy Profile (M06-D3/SDD/6-311++G(2d,2p)//M06L-D3/SDD/6-31G(d,p) corrected for DFB solvent) for the formation of **2** from **1** via carbon-to-α-carbon hydrogen atom transfer from 9,10-dihydroanthracene. Insets show α-spin density plots. Relative Gibbs Energies (298 K, 1 atm) are given in kcal·mol^{-1}.

9.3. Structures and energetics of 1·Solvent and 3·ACN

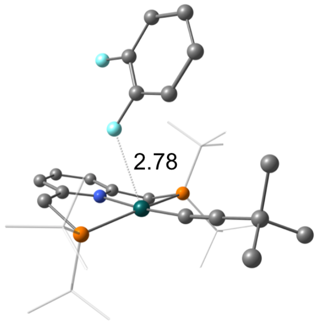

1·DFB

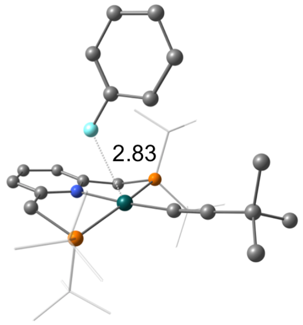

1·FB

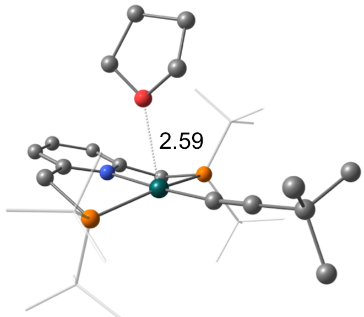

1·THF

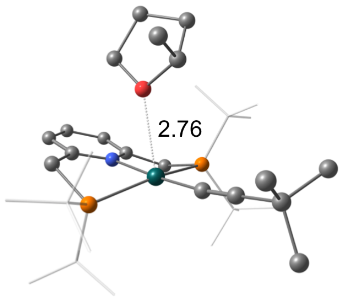

1·MeTHF

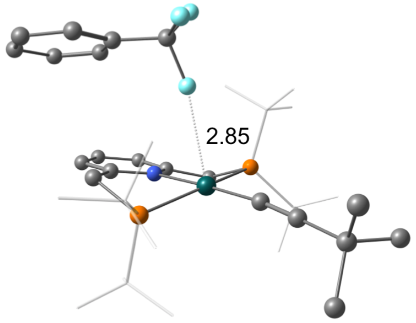

1·TFT

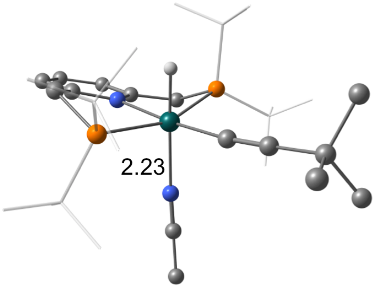

3·ACN

Figure S54. Optimised geometries (M06L-D3/SDD/6-31G**) of solvent coordinated complexes.

Table S1. Calculated enthalpies (Δ*H*) and Free energies (Δ*G*) for solvent binding to **1** (M06-D3/SDD/6-311++G(2d,2p)//M06L-D3/SDD/6-31G**).

	**Gas phase**		**Solvent corrected**	
**Reaction**	ΔH_{298K} / ΔG_{298K}	ΔH_{115K} / ΔG_{115K}	ΔH_{298K} / ΔG_{298K}	ΔH_{115K} / ΔG_{115K}
**1** + DFB → **1**·DFB	–14.4 / –2.3	–14.8 / –8.2	–7.5 / 4.6	–8.0 / –1.4
**1** + FB → **1**·FB	–13.9 / –3.2	–14.4 / –8.5	–7.9 / 2.9	–8.4 / –2.4
**1** + THF → **1**·THF	–19.4 / –5.0	–19.7 / –12.0	–11.5 / 2.8	–11.8 / –4.2
**1** + MeTHF → **1**·MeTHF	–17.1 / –2.3	–17.4 / –9.5	–9.3 / 5.5	–9.6 / –1.7
**1** + TFT → **1**·TFT	–14.6 / – 0.7	–18.6 / –3.6	–8.7 / 5.2	–12.7 / 2.2

9.4. Multireference CAS(9,6)/NEVPT2 calculations

Table S2. Dominant configurations contributing to the CAS(9,6) space for **1** (@M06L-D3/SDD/6-31G**).

CAS configuration	Energy / cm^{-1}	Weights
Root 0		
[222210]	0.00	0.96817
[222120]		0.01656
[022212]		0.00804
[122211]		0.00314
Root 1		
[222120]	987.8	0.95852
[222210]		0.01632
[022122]		0.00844
[222111]		0.00825
[122121]		0.00287
[211211]		0.00267

(a)

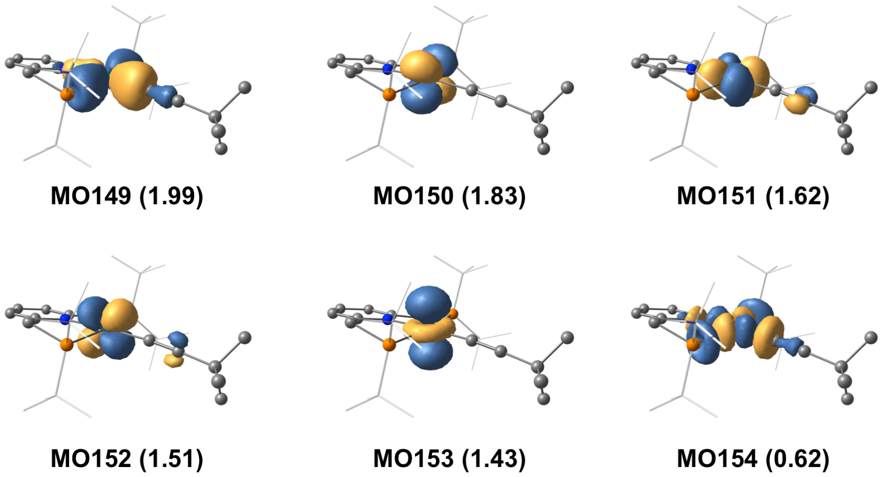

(b)

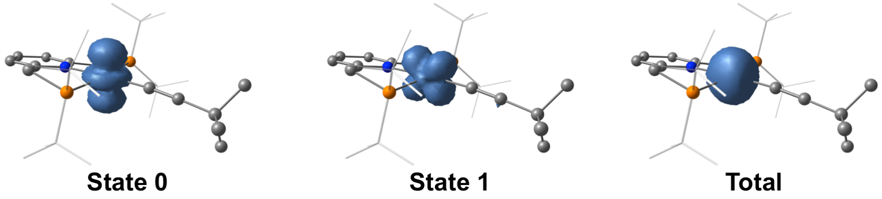

Figure S55. (a) Natural active orbitals (isosurface 0.05 au) and their occupation numbers from CAS(9,6)/NEVPT for **1**. (b) Spin densities (isosurface 0.005 au).

Table S3. Dominant configurations contributing to the CAS(9,6) space for **1** (@PBE0-D4/def2-TZVP).

CAS configuration	Energy / cm^{-1}	Weights
	Root 0	
[222210]		0.98547
[022212]	0.00	0.00787
[122211]		0.00326
	Root 1	
[222120]		0.97686
[022122]	1101.2	0.00831
[222111]		0.00704
[122121]		0.00290

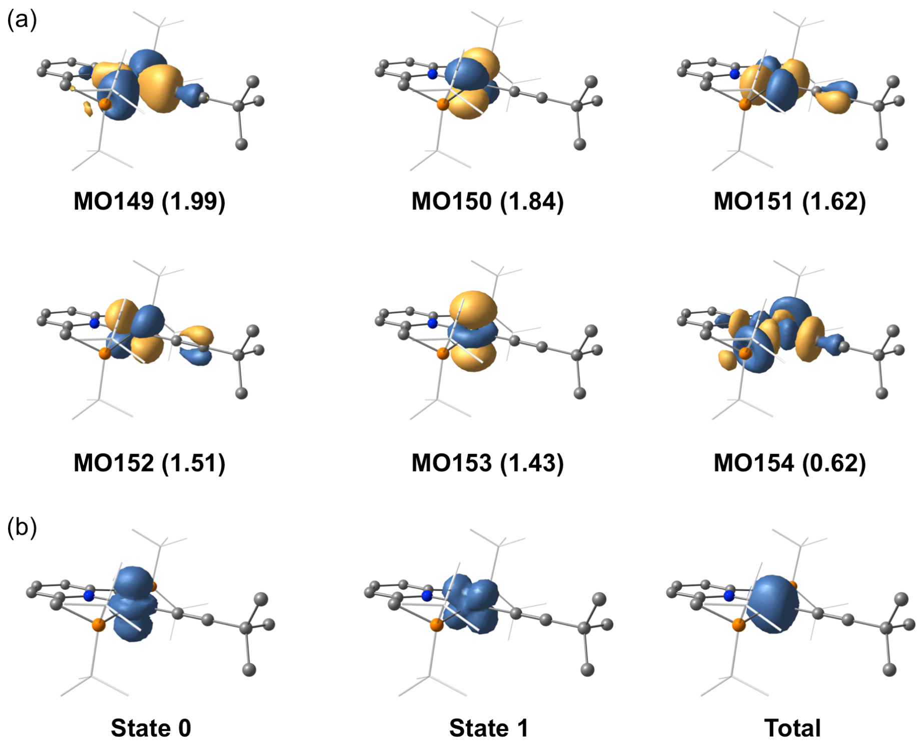

Figure S56. (a) Natural active orbitals (isosurface 0.05 au) and their occupation numbers from CAS(9,6)/NEVPT for **1**. (b) Spin densities (isosurface 0.005 au).

Table S4. Dominant configurations contributing to the CAS(9,6) space for **1**·DFB (@M06L-D3/SDD/6-31G**).

CAS configuration	Energy / cm^{-1}	Weights
Root 0		
[222210]	0.00	0.96588
[222120]		0.01858
[022212]		0.00794
[122211]		0.00299
Root 1		
[222120]	2984.8	0.94460
[222210]		0.01827
[222111]		0.01060
[221220]		0.00874
[022122]		0.00827
[211221]		0.00297
[122121]		0.00278

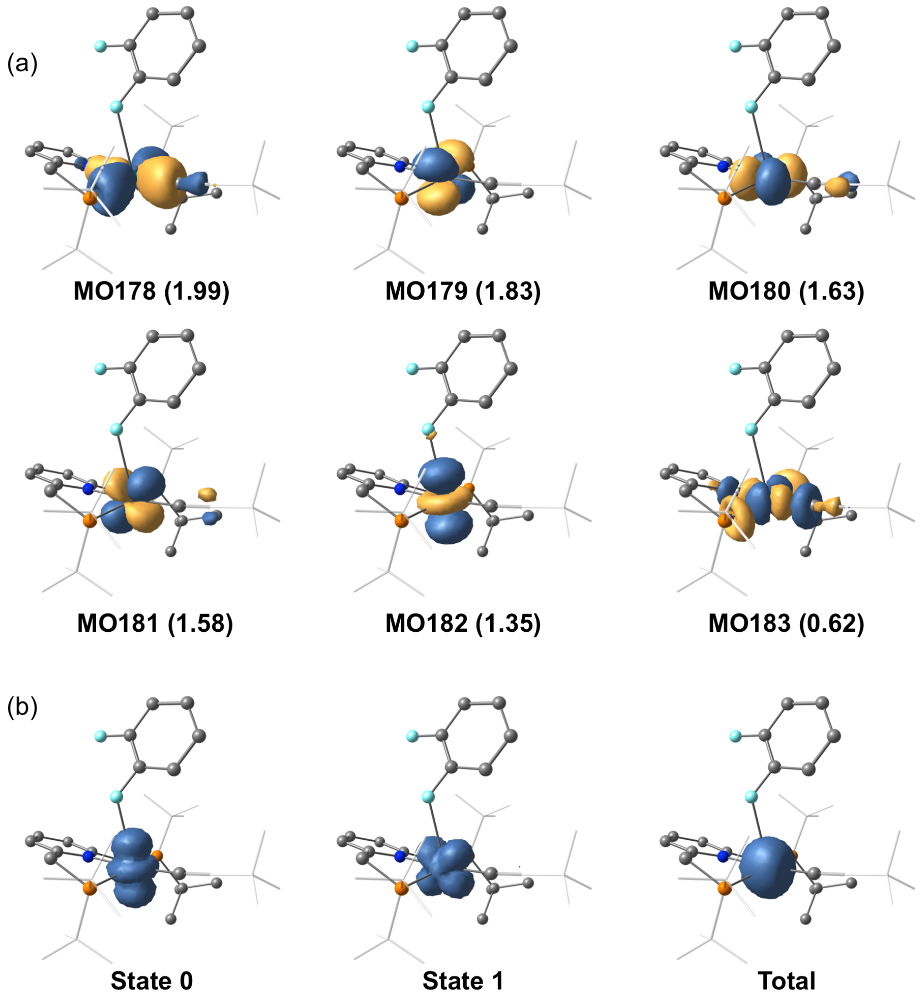

Figure S57. (a) Natural active orbitals (isosurface 0.05 au) and their occupation numbers from CAS(9,6)/NEVPT for **1**·DFB. (b) Spin densities (isosurface 0.005 au).

Table S5. Dominant configurations contributing to the CAS(9,6) space for **1**·DFB (@PBE0-D4/def2-TZVP).

**CAS configuration**	**Energy / cm^{-1}**	**Weights**
	Root 0	
[222210]	0.0	0.97794
[022212]		0.00770
[222120]		0.00747
[122211]		0.00298
	Root 1	
[222120]	4183.6	0.95823
[222111]		0.00934
[221220]		0.00835
[022122]		0.00804
[222210]		0.00732
[122121]		0.00276
[112221]		0.00267

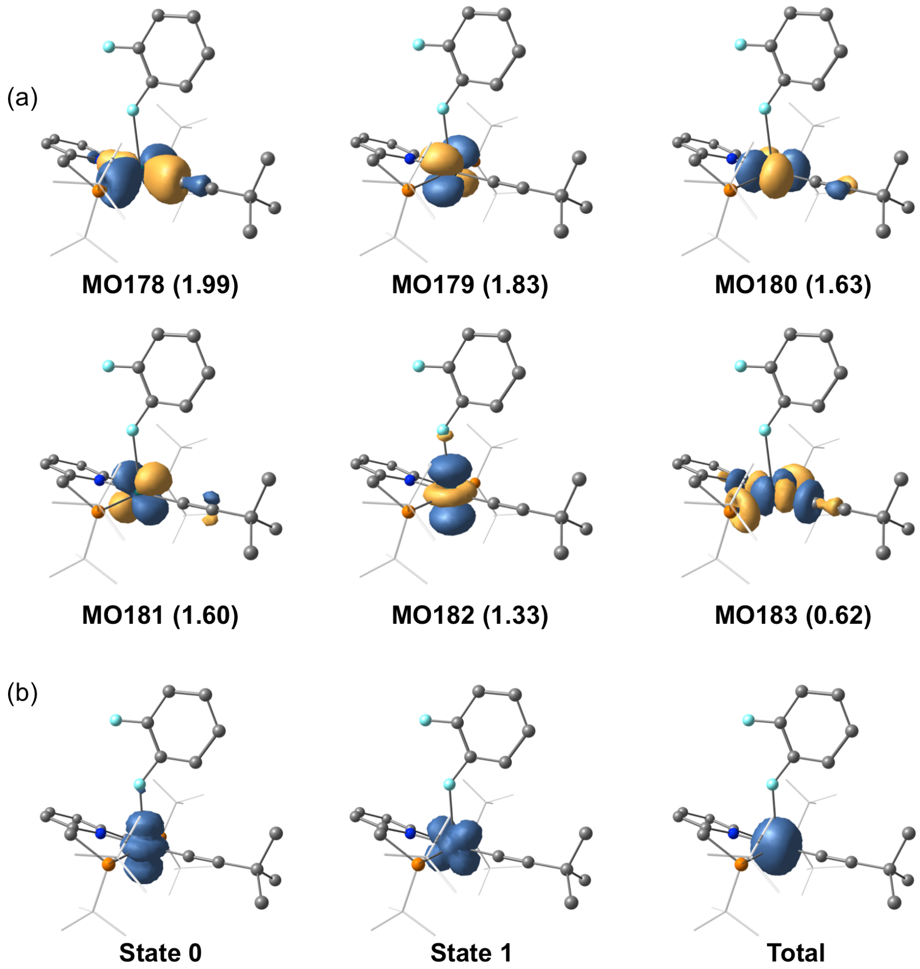

Figure S58. (a) Natural active orbitals (isosurface 0.05 au) and their occupation numbers from CAS(9,6)/NEVPT for **1**·DFB. (b) Spin densities (isosurface 0.005 au).

Table S6. Comparison of **g**-values calculated using M06L-D3/SDD/6-31G** geometries.

Complex (phase)	g_1	g_2	g_3	g_{iso}
**1** (gas)	4.547	1.361	1.167	2.358
Exp. (solid)	4.051	1.328	1.140	2.173
Exp. (DFB glass)	3.982	1.286	1.274	2.181
**1**·DFB (gas)	3.461	2.314	1.816	2.530
Exp. (DFB glass)	2.441	2.301	1.970	2.237

Table S7. Comparison of **g**-values calculated using PBE0-D4/def2-TZVP geometries.

Complex (phase)	g_1	g_2	g_3	g_{iso}
**1** (gas)	4.466	1.440	1.233	2.380
Exp. (solid)	4.051	1.328	1.140	2.173
Exp. (DFB glass)	3.982	1.286	1.274	2.181
**1**·DFB (gas)	3.111	2.388	1.900	2.466
Exp. (DFB glass)	2.441	2.301	1.970	2.237
